# Entry to new spiroheterocycles via tandem Rh(II)-catalyzed O–H insertion/base-promoted cyclization involving diazoarylidene succinimides

**DOI:** 10.3762/bjoc.20.48

**Published:** 2024-03-11

**Authors:** Alexander Yanovich, Anastasia Vepreva, Ksenia Malkova, Grigory Kantin, Dmitry Dar’in

**Affiliations:** 1 Institute of Chemistry, Saint Petersburg State University, 26 Universitetskiy pr., Peterhof, Saint Petersburg 198504, Russian Federation; 2 Saint Petersburg Research Institute of Phthisiopulmonology, 2-4 Ligovsky pr., Saint Petersburg 191036, Russian Federation; 3 Department of Medicinal Chemistry, Institute of Chemistry, Saint Petersburg State University, 26 Universitetskiy pr., Peterhof 198504, Russian Federation

**Keywords:** diazoarylidene succinimides, intramolecular cyclization, rhodium(II) carbene O–H insertion, spirocycles

## Abstract

A facile approach to novel medicinally relevant spiro heterocyclic scaffolds (namely furan-2(5*H*)-ones, tetrahydrofurans and pyrans spiro-conjugated with the succinimide ring) has been developed. The protocol consists of Rh(II)-catalyzed insertion of heterocyclic carbenes derived from diazoarylidene succinimides (DAS) into the O–H bond of propiolic/allenic acids or brominated alcohols, followed by base-promoted cyclization to afford the target spirocyclic compounds in good to high yields.

## Introduction

Spirocyclic motifs have emerged as auspicious frameworks for modern drug design [1–2]. They are known to promote higher success rates, when targeting three-dimensional protein molecules [3–4]. Furthermore, a wide variety of spirocyclic fragments can be spotted in natural products [5]. The aspects mentioned unveil the development of synthetic methodologies towards spirocyclic scaffolds as a goal of great value [6–9].

**Scheme 1:**
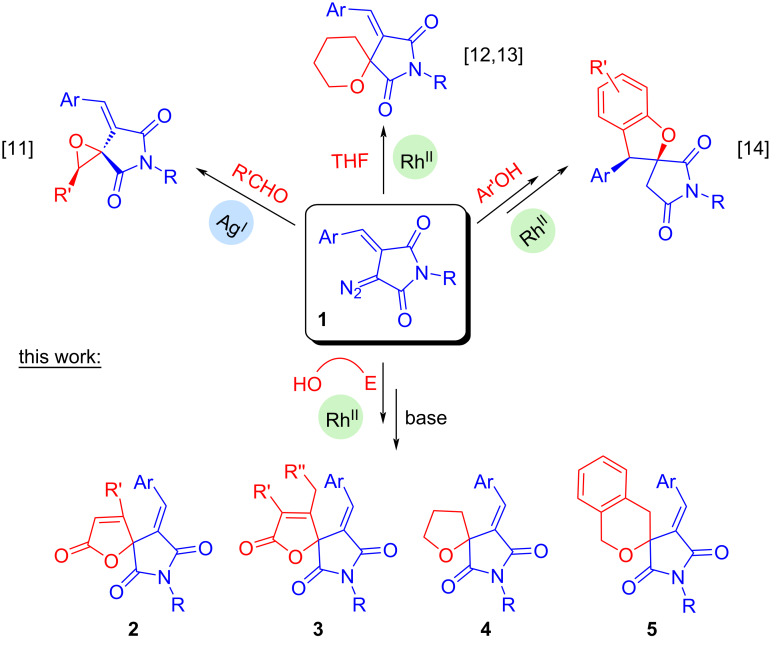
DAS spirocyclizations reported earlier and the synthetic methodology investigated in this work.

A rich synthetic platform for the design of various types of spiroheterocycles is provided by cyclic diazo compounds [10]. Recently, we and others have demonstrated the efficient use of diazoarylidene succinimides (DAS, **1**) in the synthesis of spiro-annulated pyrrolidine-2,5-diones by catalyzed spirocyclizations involving aldehydes [11], tetrahydrofuran [12–13], and in the O–H insertion/Claisen rearrangement/intramolecular oxa-Michael addition cascade [14] (Scheme 1).

Herein, we report our findings obtained, while investigating the extension of this methodology. This study is aimed at the development of convenient protocols for the synthesis of new spiroheterocycles via tandem Rh(II)-catalyzed OH insertion/base-promoted cyclization using DAS and various OH substrates containing an activated multiple bond (propiolic and allenic acids) or a bromine atom. These transformations yield spiro-annulated O-heterocycles with succinimide ring, namely spiro-Δ^α,β^-butenolides **2** and **3**, tetrahydrofurans **4** and benzopyrans **5** (Scheme 1).

Fragments of these oxygen-containing spiro-conjugated heterocycles are part of many important drugs and natural products. For example, spirocyclic Δ^α,β^-butenolides (furan-2(5*H*)-ones) represent a valuable class of molecular frameworks for drug design and are abundant in nature [15]. Bioactive naturally occurring spiro Δ^α,β^-butenolides include spirofragilide (with anti-inflammatory, antibiotic, antitumor, anti-HIV activity) [16], ramariolide A (antitubercular) [17], (+)-massarinolin A (antibacterial) [18], anemonin (antiparasitic) [19], (+)-pyrenolide D (cytotoxic) [20], and (+)-crassalactone D (antitumor) [21]. Synthetic or semisynthetic spiro Δ^α,β^-butenolides have also shown a range of biological properties including aldosterone receptor antagonistic [22], anti-inflammatory [23], and anti-HIV [24] activity.

**Figure 1:**
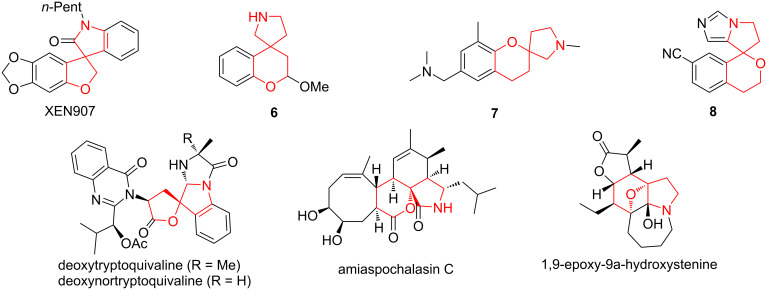
Examples of biologically active compounds and natural products based on THF/THP spiro-conjugates with pyrrolidine rings.

Substantial drugs based on spirocyclic tetrahydrofuran and pyran moieties include spironolactone (a multi-target drug that is primarily used to treat high blood pressure and heart failure) [25], drospirenone (exhibits high affinity to progesterone receptors and is used as a birth control medication) [26–27], griseofulvin (an antifungal agent used to treat fungal infections of the fingernails and toes) [28], as well as oliceridine (a selective G protein-biased μ-opioid receptor agonist used for treatment of acute severe pain) [29] and an investigational drug NOP-1A (a ligand for the nociceptin/orphanin FQ peptide (NOP) receptor which is thought to be involved in several central nervous system disorders such as anxiety, depression, drug abuse, and seizures) [30]. A wide range of biological properties are exhibited by compounds based on a THF and THP core spiro-conjugated with the pyrrolidine ring. These frameworks are present in a number of synthetic biologically active compounds (such as NaV1.7 blocker XEN907 for the treatment of pain [31], σ1 receptor ligand **6** [32], histamine-3 receptor antagonist **7** [33], and aldosterone synthase inhibitor **8** [34]) as well as natural products (e.g., new alkaloids deoxytryptoquivaline and deoxynortryptoquivaline from fungus *Aspergillus clavatonanicus* identified as natural cardiomyocyte-protective agents against cold ischemic injury [35] and possible natural multitarget drugs against COVID-19 [36], and amiaspochalasin C isolated from the solid culture of *Aspergillus micronesiensis* [37] and 1,9-epoxy-9a-hydroxystenine from the roots of *Stemona tuberosa* [38]) (Figure 1).

Hence, the development of novel synthetic methods to construct spiro *O*-heterocycles constitutes a distinctly worthy undertaking which may as well influence the outlook of the novel medicines discovered and developed in the future.

## Results and Discussion

**Scheme 2:**
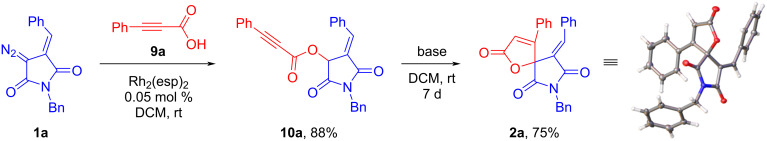
An initial example on Rh(II)-catalyzed O–H insertion/base-promoted cyclization involving diazo compound **1a**.

As a first step, we turned to studying the possibility of obtaining spirocyclic butenolides from DAS **1**, based on our previously proposed approach using propiolic acids [39]. The diazo reagent **1a** was introduced in the Rh_2_(esp)_2_-catalyzed insertion into the O–H bond of phenylpropropiolic acid (**9а**) to form the intermediate compound **10a** (Scheme 2). The cyclization of the latter was carried out in DCM solution under the action of DIPEA (30 mol %). Under these conditions, the 5-*endo-dig* cyclization leading to the target spirobutenolide **2a** proceeded rather slowly (about 25% conversion per day). However, an attempt to accelerate the reaction by using a stronger base (DBU) resulted in side processes with the formation of unwanted impurities, whereas the reaction in the presence of DIPEA proceeded selectively, albeit more slowly. By increasing the DIPEA loading to 50 mol %, the product **2a** was isolated in 75% yield after incubation for 7 days at room temperature. The structure of the obtained spirobutenolide was confirmed by single crystal X-ray data.

**Scheme 3:**
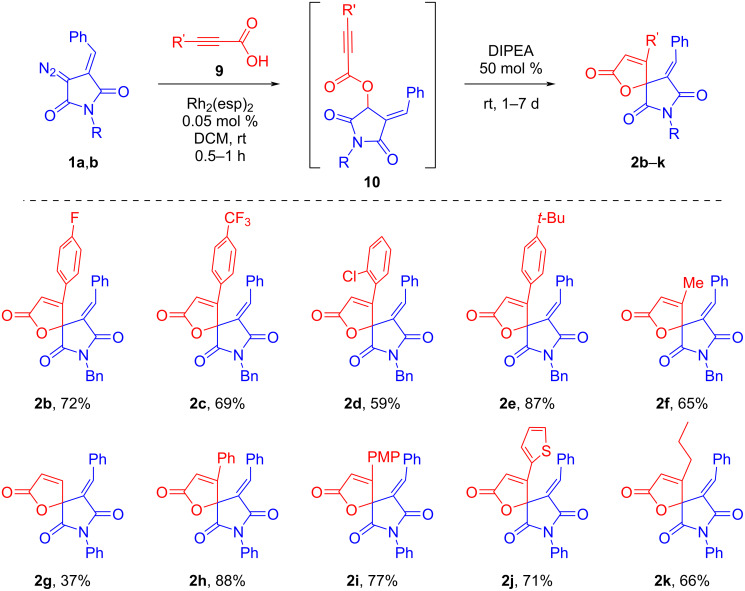
Tandem Rh_2_(esp)_2_-catalyzed O–H insertion/base-promoted cyclization involving DAS **1** and various propiolic acids; PMP = 4-methoxyphenyl.

Further syntheses of spirobutenolides **2** were performed under the one-pot conditions: after completion of the first O–H insertion step, a base was added to the reaction mixture and kept at room temperature until completion of the cyclization step, controlled by TLC. The results of the syntheses carried out with different substituted propiolic acids **9** and DAS **1** are shown in Scheme 3. It can be noted that in the case of arylpropiolic acids, no significant influence of electronic effects of substituents in the aromatic ring was observed. In the case of the *o*-chloro derivative **2d** the yield was slightly reduced, which can be attributed to the influence of the steric factor. The transition to alkyl-substituted (Me and *n-*Pr) propiolic acids did not significantly affect the yields of the final products **2f** and **2k**, which were isolated in 65% and 66% yields, respectively. A moderate yield (37%) was obtained when unsubstituted propiolic acid was used as OH-substrate (**2g**). The reasons for this result may be due to the increased reactivity of the terminal triple bond of the propiolic moiety, which favors the participation of the OH-insertion intermediate in side processes. However, we were unable to isolate or otherwise identify any byproducts in this case.

**Scheme 4:**
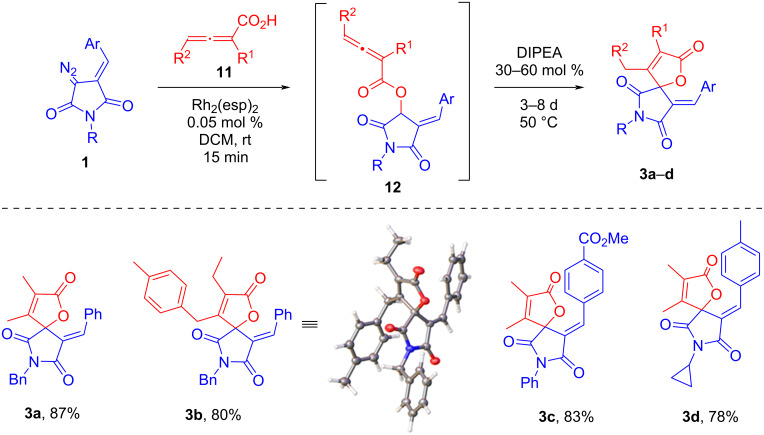
Tandem Rh_2_(esp)_2_-catalyzed O–H insertion/base-promoted cyclization involving DAS **1** and allenic acids.

Recently, we have shown that this approach to the synthesis of spirocyclic butenolides can also be realized using allenic acids [40]. This opens up the possibility of obtaining target spiroheterocycles with substituents not only in the beta but also in the alpha position of the furanone ring. Reactions with allenic acids **11** were carried out according to a similar scheme, in the one-pot mode, without isolation of OH-insertion intermediates **12** (Scheme 4). In order to accelerate the cyclization step in this case, moderate heating was used after the addition of base (DIPEA).

The target α,β-disubstituted spirobutenolides **3a–d** were isolated in high yields irrespective of the change in the type of substitution in the initial DAS. However, in the case of **3d**, the cyclization stage proceeded slower (8 days instead of 3) and under an elevated amount of DIPEA (60 mol %), which can be explained by the presence of EDG in the corresponding DAS. In general, the second step of the process appears to occur as *endo*-cyclization onto an activated multiple bond followed by migration of the remaining endocyclic double bond into the furanone ring. The structure of product **3b** has been confirmed by crystallographic data.

An approach to the construction of the THF cycle using a diazo reagent and 3-bromopropan-1-ol (**13**) [41] or similar halogenated OH substrates [42] has already been demonstrated in the literature using selected examples. We first validate this protocol for spirocyclization and spiroheterocycle formation.

**Scheme 5:**
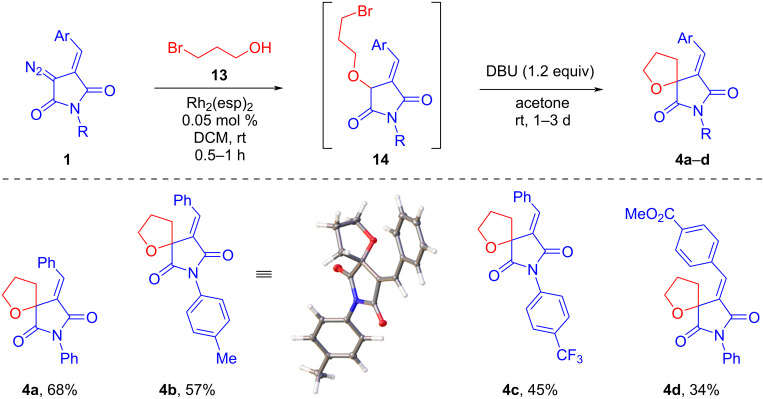
Tandem Rh_2_(esp)_2_-catalyzed O–H insertion/base-promoted cyclization involving various DAS **1** and 3-bromopropanol.

The first step of the synthesis, the insertion of rhodium carbene into the O–H bond of 3-bromopropanol, was carried out under standard conditions in the presence of 0.05 mol % Rh_2_(esp)_2_ in dry DCM. ^1^H NMR spectroscopy was used to monitor the progress of the reaction and the formation of the OH-insertion product **14**. An attempt to carry out the second step in a one-pot format with the addition of 1.2 equiv of base (DIPEA or DBU) was unsuccessful and the formation of the spirocyclic product was not observed. Replacing DCM with a more polar solvent, acetone, significantly accelerated the cyclization process. Thus, one to three days were required to complete the 5-*exo-tet* cyclization process in the acetone/DBU system. The results of the syntheses carried out with the participation of various DAS **1** to obtain spirocyclic THFs are presented in Scheme 5.

As can be seen, the yields of the target compounds **4** vary from good to moderate per two steps of synthesis. The introduction of acceptor substituents in both the aniline and arylidene moieties of the DAS molecule leads to a decrease in the yield of the final spirocycle. The structure of compound **4b** has been confirmed by single crystal X-ray data.

**Scheme 6:**
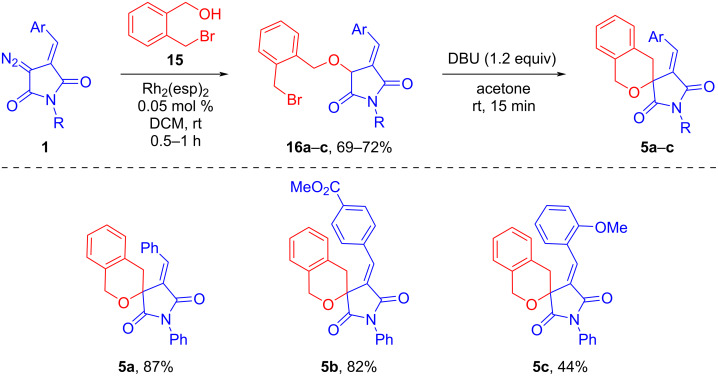
Tandem Rh_2_(esp)_2_-catalyzed O–H insertion/base-promoted cyclization involving DAS **1** and 2-(bromomethyl)benzyl alcohol.

The next step was to investigate the possibility of obtaining six-membered oxygen-containing spiroheterocycles by interaction of DAS **1** with 2-(bromomethyl)benzyl alcohol (**15**) (Scheme 6). The synthesis was carried out under the conditions previously tested using 3-bromopropanol. When the reaction was carried out in a one-pot format, with the replacement of DCM by acetone, the desired product **5a** could only be isolated in moderate yield (52%). In the case of preliminary isolation of the OH-insertion product **16a** (flash chromatography), the cyclization step was more selective and the total yield of the desired product was higher.

The 6-*exo-tet* cyclization of intermediate compounds **16** under the action of DBU in acetone at room temperature proceeded within a few minutes, which is much faster than in the case of compounds **14**. This can be explained by the higher reactivity of benzyl bromide and the lower conformational mobility of the side chain with the *ortho*-phenylene link. As a result, new spirocyclic compounds **5** were obtained in high (**5a**,**b**) or moderate (**5c**) yields.

**Scheme 7:**
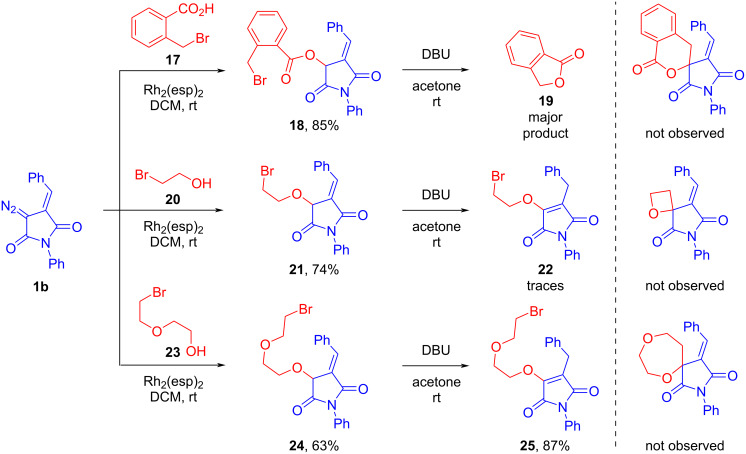
Examples where a target spirocyclic product was not observed.

With some other bromo-substituted OH substrates, we obtained O–H insertion reaction products using diazo reagent **1b** as an example, but we failed to observe the formation of spirocyclic cyclization products as a result of intramolecular substitution of the bromine atom (Scheme 7). For example, during the attempted cyclization of compound **18**, obtained from 2-(bromomethyl)benzoic acid (**17**), the formation of a complex mixture was observed, the main component of which turned out to be phthalide (**19**). The use of 2-bromoethanol (**20**) gave a similar result – compound **21** was transformed under the action of DBU into a multicomponent mixture of unidentifiable compounds. Judging from the ^1^H NMR spectroscopy data, one of its components was the product of the migration of the double bond of the benzylidene fragment into the succinimide cycle – compound **22**. Finally, a compound with a longer side chain **24**, obtained from 2-(2-bromoethoxy)ethanol (**23**), underwent exclusively isomerization under basic conditions resulting in achiral product **25**. No proton signals related to the expected cyclization product were detected in the proton NMR spectrum.

**Scheme 8:**
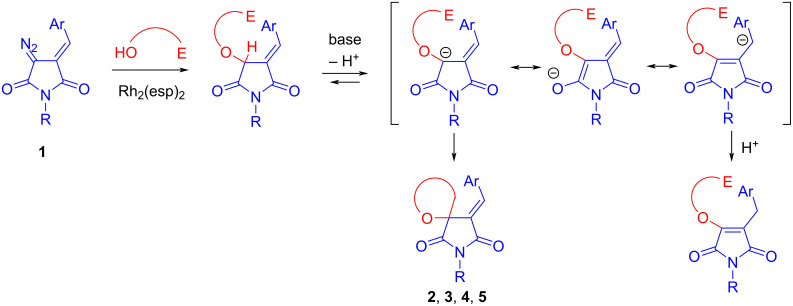
Plausible mechanism of the transformations studied.

The formation of phthalide from compound **18** under the action of base is difficult to explain. In this case, for some reason, the nucleophilic attack of the oxygen atom of the ester group on the benzyl bromide residue prevails, with the cleavage of the arylidene succinimide fragment involved in further non-selective processes. The causes of the failed cyclizations in the last two cases can be summarized as follows. The intermediates obtained from each of the bromo-substituted alcohols used by us have two main pathways of transformation under the action of base: 1) *exo-tet* cyclization with substitution of the bromine atom and formation of the spirocycle, and 2) migration of the exocyclic double C=C bond into the imide cycle (the process is summarized in Scheme 8). The first pathway is realized in the formation of five-membered (in the case of 3-bromopropanol) and six-membered (in the case of 2-(bromomethyl)benzyl alcohol) cycles – the cyclization of the anion is faster than its reverse protonation. The same applies to substrates with activated multiple bonds (**10** and **12**). However, in the case of shorter (from 2-bromoethanol) and longer (compound **24**) chain intermediates, the cyclization is retarded due to the disadvantage of forming a strained four-membered ring in the former case and a significant loss of entropy during the formation of a seven-membered cycle in the latter case. The main direction of the reaction in these examples becomes isomerization (migration of a proton when it is captured by an intermediate anion) or other side processes.

In the conjugated anion formed as a result of deprotonation (Scheme 8), one would also expect cyclization involving the γ-position, but we did not observe the formation of such products. This is probably due to the less favorable formation of cycles of larger size and also in view of the significantly greater contribution of the resonance structure with negative charge on the oxygen atom and, as a consequence, the significantly greater nucleophilicity of the nearest α-carbon atom. When an attempt was made to generate an anion from compound **25** under the action of a stronger base (*t-*BuOK/THF, 0 °C) in order to effect spirocyclization, only the formation of a complex multicomponent mixture was observed.

## Conclusion

We have devised a straightforward access to novel spiro-annulated *O*-heterocyclic frameworks based on Rh_2_(esp)_2_-catalyzed insertion of carbenes derived from diazoarylidene succinimides (DAS) into the O–H bond of propiolic or allenic acids, as well as 3-bromopropan-1-ol and 2-(bromomethyl)benzyl alcohol followed by base-promoted cyclization. The procedures developed allow to obtain derivatives of such sought-after scaffolds in the field of medicinal chemistry as Δ^α,β^-butenolides, tetrahydrofurans, and pyrans spiro-conjugated with a pyrrolidine ring. The tandem approach proposed is characterized by mild synthetic conditions and high or good yields of the target compounds after two steps. The limitations of the method were demonstrated by unsuccessful attempts to carry out the cyclization of ОН-insertion products derived from 2-(bromomethyl)benzoic acid, 2-bromoethanol, and 2-(2-bromoethoxy)ethanol. In the latter case, the predominant process was found to be the base-promoted migration of the C=C bond of the arylidene fragment into the cycle.

## Supporting Information

Deposition numbers CCDC 2295111 (for **2a**), 2308315 (for **3b**), and 2305370 (for **4b**) contain the supplementary crystallographic data for this paper. These data are provided free of charge by the joint Cambridge Crystallographic Data Centre and Fachinformationszentrum Karlsruhe Access Structures service https://www.ccdc.cam.ac.uk/structures.

File 1 General experimental information, X-ray crystallographic data, synthetic procedures, analytical data and NMR spectra for the reported compounds.

## Data Availability

All data that supports the findings of this study is available in the published article and/or the supporting information to this article.
