# Supplementary material for: Entry to new spiroheterocycles via tandem Rh(II)-catalyzed O–H insertion/base-promoted cyclization involving diazoarylidene succinimides
Source: Beilstein J Org Chem. 2024 Mar 11;20:561–9. doi: 10.3762/bjoc.20.48 (PMC10949003; doi:10.3762/bjoc.20.48)
Supplement: File 1 — General experimental information, X-ray crystallographic data, synthetic procedures, analytical data and NMR spectra for the reported compounds. [file Beilstein_J_Org_Chem-20-561-s001.pdf]

BEILSTEIN JOURNAL OF ORGANIC CHEMISTRY

Supporting Information

for

Entry to new spiroheterocycles via tandem Rh(II)-catalyzed O–H insertion/base-promoted cyclization involving diazoarylidene succinimides

Alexander Yanovich, Anastasia Vepreva, Ksenia Malkova, Grigory Kantin and Dmitry Dar’in

Beilstein J. Org. Chem. **2024,** *20,* 561–569. doi:10.3762/bjoc.20.48

General experimental information, X-ray crystallographic data, synthetic procedures, analytical data and NMR spectra for the reported compounds

License and Terms: This is a supporting information file under the terms of the Creative Commons Attribution License (https://creativecommons.org/licenses/by/4.0). Please note that the reuse, redistribution and reproduction in particular requires that the author(s) and source are credited and that individual graphics may be subject to special legal provisions.
The license is subject to the *Beilstein Journal of Organic Chemistry* terms and conditions: (https://www.beilstein-journals.org/bjoc/terms)

Table of contents

I. Materials and methods..S2

II. Experimental procedures for the synthesis of target compounds S3

III. NMR spectra S12

IV. X-ray crystallographic data...S40

V. References S43

I. Materials and methods

Commercial grade reagents and solvents were used without further purification except as indicated below. Dichloromethane (DCM) was distilled from phosphorus pentoxide.

NMR spectra were recorded using a Bruker Avance III spectrometer in $CDCl_3$ (^1H: 400.13 MHz, ^{13}C: 100.61 MHz; ^{19}F: 376.50 MHz). All chemical shifts are reported in parts per million (ppm). The residual solvent peak was used as internal standard: $CDCl_3$ (7.26 for ^1H and 77.16 ppm for ^{13}C). Standard abbreviations were used in the description of resonances. Coupling constants (*J*) are quoted to the nearest 0.1 Hz. Mass spectra were recorded with a HRMS-ESI-qTOF spectrometer (electrospray ionization mode, positive ion detection). Melting points were determined with a melting point apparatus Stuart SMP 10 in open capillary tubes. Single crystal X-ray data were obtained using SuperNova, Single source at offset/far, HyPix3000 and SuperNova, Dual, Cu at home/near, Atlas diffractometers at a temperature of 100 K.

Analytical thin layer chromatography was carried out on UV-254 silica gel plates using appropriate eluents. Compounds were visualized with short wavelength UV light. Column chromatography was performed using silica gel Merck grade 60 (0.040–0.063 mm) 230–400 mesh.

Starting diazo compounds **1** (Figure S1) were obtained according to the literature procedure [1].

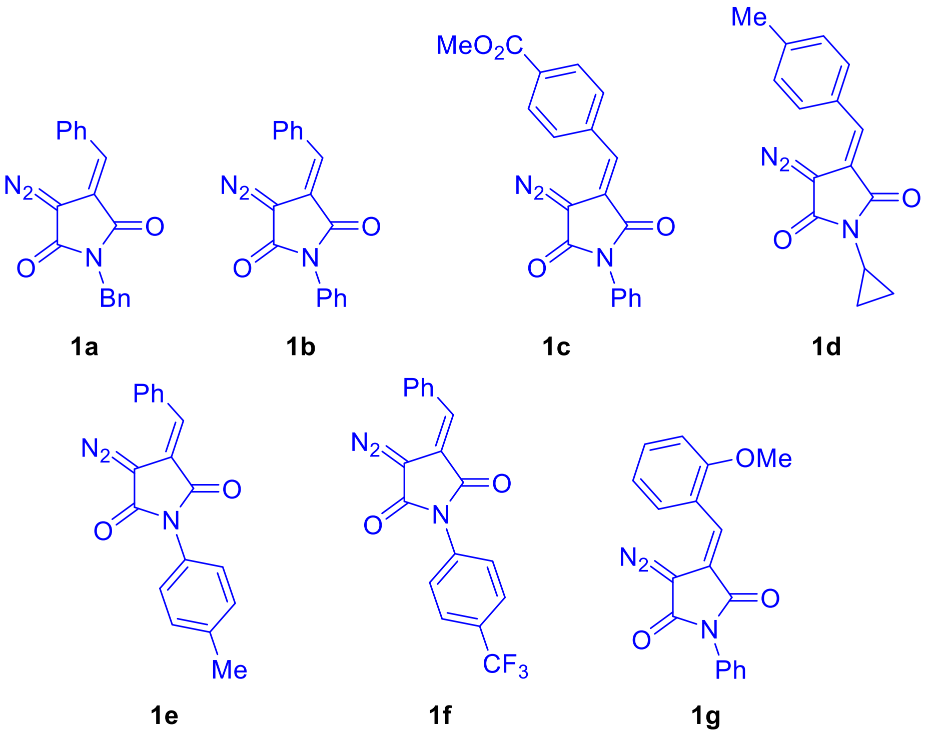

Figure S1. Diazo compounds **1a–g** used in this work.

II. Experimental procedures for the synthesis of target compounds

Preparation of compound 2a

(E)-7-Benzyl-9-benzylidene-4-phenyl-1-oxa-7-azaspiro[4.4]non-3-ene-2,6,8-trione (2a)

To a stirred solution of 3-phenylpropiolic acid (161 mg, 1.1 mmol, 1.1 equiv) in dry DCM (1.6 mL) was added the catalyst solution (400 µL of 2.5 mM $Rh_2(esp)_2$ in DCM, 0.05 mol %) followed by dropwise addition of the solution of diazo compound **1a** (303 mg, 1.0 mmol) in dry DCM (2 mL). The reaction mixture was stirred at ambient temperature for 1 h (controlled by TLC), diluted with *n*-hexane (2 mL) and the resulting solution was subjected to column chromatography on silica gel (eluent – *n*-hexane/DCM, from 20 to 70% of DCM) to afford 371 mg (88%) of compound **10a** as a white amorphous solid. **^{1}H NMR (400 MHz, $CDCl_3$):** δ 7.83 (d, J = 2.2 Hz, 1H), 7.61 – 7.56 (m, 2H), 7.56 – 7.52 (m, 2H), 7.53 – 7.43 (m, 7H), 7.42 – 7.29 (m, 5H), 6.25 (d, J = 2.2 Hz, 1H), 4.89 (d, J = 14.2 Hz, 1H), 4.84 (d, J = 14.2 Hz, 1H).

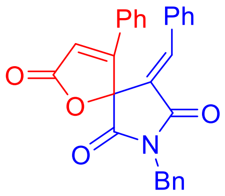

The product of the previous step (211 mg, 0.5 mmol) was dissolved in dry DCM (2 mL) followed by addition of DIPEA (32 mg 0.25 mmol, 50 mol %) and left stirring at room temperature for 7 d (controlled by TLC). The reaction mixture was diluted with *n*-hexane (2 mL) and the resulting solution was subjected to column chromatography on silica gel (eluent – *n*-hexane/DCM, from 60 to 100% of DCM) to afford 90 mg (75%) of the title compound **2a**. White solid, mp 171.1–171.4 °C. **^{1}H NMR (400 MHz, $CDCl_3$)**: δ 8.07 (s, 1H), 7.43 m, 2H), 7.42 – 7.35 (m, 1H), 7.33 (m, 4H), 7.17 (d, J = 7.5 Hz, 2H), 7.06 (t, J = 7.5 Hz, 2H), 6.73 – 6.69 (m, 2H), 6.37 (s, 1H), 4.97 (d, J = 13.9 Hz, 1H), 4.89 (d, J = 13.9 Hz, 1H). **^{13}C{^{1}H} NMR (101 MHz, $CDCl_3$)**: δ 170.1, 169.3, 167.7, 162.6, 144.9, 134.5, 132.1, 131.3, 130.9, 129.6, 129.1, 128.9, 128.90, 128.5, 128.5, 126.6, 124.0, 118.9, 84.4, 43.7. **HRMS (ESI-TOF)**, *m/z* calcd for $C_{27}H_{20}NO_4$ $[M+H]^+$ 422.1387 found 422.1392.

General procedure (GP1) for the synthesis of compounds 2

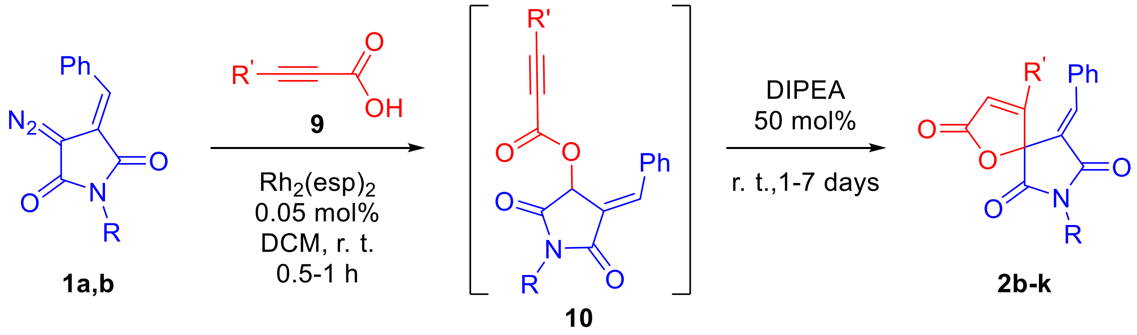

To a stirred solution of the corresponding propiolic acid (0.55 mmol, 1.1 equiv) in dry DCM (0.8 mL) was added the catalyst solution (200 µL of 2.5 mM $Rh_2(esp)_2$ in DCM, 0.05 mol %) followed by dropwise addition of the solution of diazo compound **1** (1.0 mmol, 1.0 equiv) in dry DCM (1 mL). The reaction mixture was stirred at ambient temperature for 0.5–1 h (controlled by TLC). Upon completion of reaction, a solution of DIPEA (32 mg, 0.25 mmol, 50 mol %) in dry DCM (0.5 mL) was added and the reaction mixture was stirred at room temperature for 1–7 d (controlled by TLC). The reaction mixture was diluted with *n*-hexane (2 mL) and the resulting solution was subjected to column chromatography on silica gel to afford pure compounds **2**.

(E)-7-Benzyl-9-benzylidene-4-(4-fluorophenyl)-1-oxa-7-azaspiro[4.4]non-3-ene-2,6,8-trione (2b)

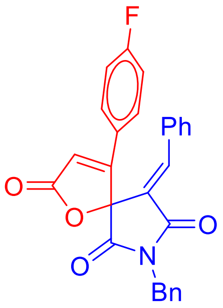

Prepared according to the general procedure **GP1** from diazo compound **1a** and 3-(4-fluorophenyl)propiolic acid (scale – 1.0 mmol). Yield: 315 mg (72%). Eluent – *n*-hexane/ethyl acetate (from 0 to 20% of ethyl acetate). White solid, mp 188.1–188.4 °C. **^{1}H NMR (400 MHz, CDCl$_3$)**: δ 8.08 (s, 1H), 7.43 (m, 2H), 7.41 – 7.37 (m, 1H), 7.37 – 7.33 (m, 4H), 7.33 – 7.30 (m, 1H), 7.19 – 7.15 (m, 2H), 6.76 – 6.70 (m, 2H), 6.70 – 6.65 (m, 2H), 6.33 (s, 1H), 4.97 (d, *J* = 13.8 Hz, 1H), 4.87 (d, *J* = 13.8 Hz, 1H). **^{13}C{^{1}H} NMR (101 MHz, CDCl$_3$)**: δ 169.9, 169.2, 167.5, 164.2 (d, $J_{C\text{-}F}$ = 254.3 Hz), 161.4, 145.0, 134.5, 132.1, 131.0, 129.6, 129.0 (d, $J_{C\text{-}F}$ = 19.8 Hz), 128.8, 128.8, 128.6, 124.7 (d, $J_{C\text{-}F}$ = 3.5 Hz), 123.8, 118.9 (d, $J_{C\text{-}F}$ = 1.5 Hz), 116.4, 116.2, 84.3, 43.7. **^{19}F{^{1}H} NMR (376 MHz, CDCl$_3$)**: δ -106.8. **HRMS (ESI-TOF)**, *m/z* calcd for $C_{27}H_{19}FNO_4$ [M+H]$^+$ 440.1293 found 440.1296.

(E)-7-Benzyl-9-benzylidene-4-(4-(trifluoromethyl)phenyl)-1-oxa-7-azaspiro[4.4]non-3-ene-2,6,8-trione (2c)

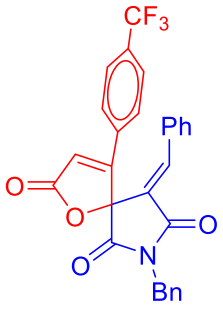

Prepared according to the general procedure **GP1** from diazo compound **1a** and 3-(4-(trifluoromethyl)phenyl)propiolic acid (scale – 1.0 mmol). Yield: 339 mg (69%). Eluent – *n*-hexane/acetone (from 10 to 50% of DCM). White solid, mp 165.1–165.2 °C. **^{1}H NMR (400 MHz, CDCl$_3$):** δ 8.09 (s, 1H), 7.42 (m, 3H), 7.38 – 7.32 (m, 5H), 7.29 (s, 1H), 7.27 (s, 1H), 7.22 – 7.14 (m, 2H), 6.75 (d, *J* = 8.1 Hz, 2H), 6.42 (s, 1H), 4.99 (d, *J* = 13.8 Hz, 1H), 4.87 (d, *J* = 13.8 Hz, 1H). **^{13}C{^{1}H} NMR (101 MHz, CDCl$_3$)**: δ 169.4, 168.9, 167.1, 160.9, 145.3, 134.5, 132.8 (q, $J_{C\text{-}F}$ = 33.1 Hz), 131.9, 131.8, 131.2, 129.6, 129.1, 128.9, 128.7, 128.6, 126.9, 125.9 (q, $J_{C\text{-}F}$ = 3.7 Hz), 123.31, 122.8 (q, $J_{C\text{-}F}$ = 273.7 Hz), 121.0, 84.3, 43.7. **^{19}F{^{1}H} NMR (376 MHz, CDCl$_3$)**: δ -63.3. **HRMS (ESI-TOF)**, *m/z* calcd for **HRMS (ESI-TOF)**, *m/z* calcd for $C_{28}H_{19}F_3NO_4$ [M+H]$^+$ 490.1261 found 490.1270.

(E)-7-Benzyl-9-benzylidene-4-(2-chlorophenyl)-1-oxa-7-azaspiro[4.4]non-3-ene-2,6,8-trione (2d)

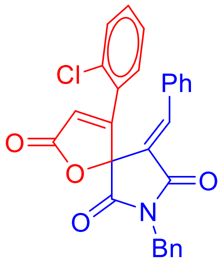

Prepared according to the general procedure **GP1** from diazo compound **1a** and 3-(2-chlorophenyl)propiolic acid (scale – 1.0 mmol). Yield: 268 mg (59%). Eluent – *n*-hexane/acetone (from 10 to 40% of acetone). White solid, mp 179.4–179.6 °C. **^{1}H NMR (400 MHz, CDCl$_3$)**: δ 8.03 (s, 1H), 7.49 – 7.32 (m, 9H), 7.25 (d, *J* = 8.1 Hz, 1H), 7.16 (t, *J* = 7.7 Hz, 1H), 6.68 (m, 2H), 6.38 (d, *J* = 7.9 Hz, 1H), 4.97 (d, *J* = 13.9 Hz, 1H), 4.83 (d, *J* = 13.9 Hz, 1H). **^{13}C{^{1}H} NMR (101 MHz, CDCl$_3$)**: δ 169.9, 169.3, 167.6, 158.4, 145.0, 134.4, 133.4, 132.1, 132.1, 131.3, 131.0, 130.3, 129.3, 128.9, 128.9, 128.5, 127.8, 127.3, 126.8, 124.4, 122.3, 85.5, 43.7. **HRMS (ESI-TOF)**, *m/z* calcd for $C_{27}H_{19}ClNO_4$ [M+H]$^+$ 456.0997 found 456.0992.

(E)-7-Benzyl-9-benzylidene-4-(4-(tert-butyl)phenyl)-1-oxa-7-azaspiro[4.4]non-3-ene-2,6,8-trione (2e)

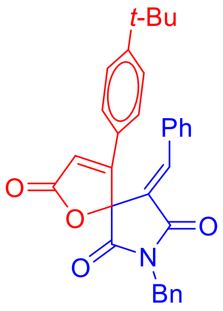

Prepared according to the general procedure **GP1** from diazo compound **1a** and 3-(4-(*tert*-butyl)phenyl)propiolic acid (scale – 0.5 mmol). Yield: 135 mg (87%). Eluent – *n*-hexane/acetone (from 10 to 40% of acetone). White solid, mp 176.1–177.7 °C. **^{1}H NMR (400 MHz, CDCl$_3$)**: δ 8.08 (s, 1H), 7.46 – 7.40 (m, 2H), 7.40 – 7.29 (m, 6H), 7.22 – 7.17 (m, 2H), 7.11 – 7.04 (m, 2H), 6.77 – 6.72 (m, 2H), 6.37 (s, 1H), 4.97 (d, *J* = 13.9 Hz, 1H), 4.90 (d, *J* = 13.9 Hz, 1H), 1.25 (s, 9H). **^{13}C{^{1}H} NMR (101 MHz, CDCl$_3$)**: δ 170.4, 169.4, 167.8, 162.6, 155.2, 144.7, 134.6, 132.1, 130.8, 129.7, 129.1, 128.9, 128.5, 128.4,

126.5, 126.0, 125.4, 124.1, 117.6, 84.3, 43.6, 34.9, 30.9. **HRMS (ESI-TOF)**, *m/z* calcd for $C_{31}H_{28}NO_3$ $[M+H]^+$ 478.2013 found 478.2021.

(E)-7-Benzyl-9-benzylidene-4-methyl-1-oxa-7-azaspiro[4.4]non-3-ene-2,6,8-trione (2f)

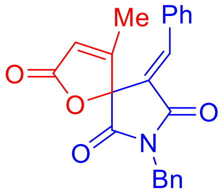

Prepared according to the general procedure **GP1** from diazo compound **1a** and but-2-ynoic acid (scale – 1.0 mmol). Yield: 233 mg (65%). Eluent – *n*-hexane/acetone (from 10 to 40% of acetone). White solid, mp 167.2–168.0 °C. **^{1}H NMR (400 MHz, CDCl$_3$)**: δ 8.15 (s, 1H), 7.46 – 7.28 (m, 9H), 6.00 (q, *J* = 1.5 Hz, 1H), 4.90 (d, *J* = 14.0 Hz, 1H), 4.84 (d, *J* = 14.0 Hz, 1H), 1.61 (d, *J* = 1.5 Hz, 3H). **^{13}C{^{1}H} NMR (101 MHz, CDCl$_3$)**: δ 170.7, 169.3, 167.7, 162.5, 144.5, 134.9, 131.9, 131.2, 129.9, 128.9, 128.8, 128.7, 128.4, 122.5, 120.5, 85.8, 43.6, 12.8. **HRMS (ESI-TOF)**, *m/z* calcd for $C_{22}H_{18}NO_4$ $[M+H]^+$ 360.1230 found 360.1233.

(E)-9-Benzylidene-7-phenyl-1-oxa-7-azaspiro[4.4]non-3-ene-2,6,8-trione (2g)

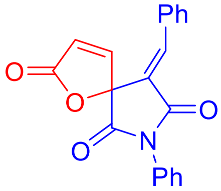

Prepared according to the general procedure **GP1** from diazo compound **1b** and propiolic acid (scale – 1.0 mmol). Yield: 123 mg (37%). Eluent – *n*-hexane/acetone (from 5 to 15% of acetone). White solid, mp 231.4–231.6 °C. **^{1}H NMR (400 MHz, CDCl$_3$)**: δ 8.28 (s, 1H), 7.55 (m, 2H), 7.51 – 7.35 (m, 8H), 7.16 (d, *J* = 5.6 Hz, 1H), 6.38 (d, *J* = 5.6 Hz, 1H). **^{13}C{^{1}H} NMR (101 MHz, CDCl$_3$)**: δ 170.8, 168.1, 166.7, 149.8, 145.9, 132.0, 131.3, 131.2, 130.0, 129.4, 129.3, 128.9, 126.1, 125.4, 122.3, 84.2. **HRMS (ESI-TOF)**, *m/z* calcd for $C_{20}H_{14}NO_4$ $[M+H]^+$ 332.0917 found 332.0921.

(E)-9-Benzylidene-4,7-diphenyl-1-oxa-7-azaspiro[4.4]non-3-ene-2,6,8-trione (2h)

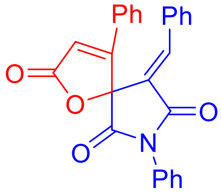

Prepared according to the general procedure **GP1** from diazo compound **1b** and 3-phenylpropiolic acid (scale – 0.5 mmol). Yield: 164 mg (88%). Eluent – *n*-hexane/acetone (from 10 to 50% of acetone). White solid, mp 182.2–183.4 °C. **^{1}H NMR (400 MHz, CDCl$_3$)**: δ 8.22 (s, 1H), 7.54 (m, 2H), 7.48 (m, 1H), 7.43 (m, 4H), 7.35 (m, 4H), 7.26 (d, *J* = 7.1 Hz, 2H), 7.12 (d, *J* = 7.1 Hz, 2H), 6.49 (s, 1H). **^{13}C{^{1}H} NMR (101 MHz, CDCl$_3$)**: δ 170.0, 168.8, 167.1, 162.5, 145.8, 132.1, 131.7, 131.3, 131.1, 129.8, 129.4, 129.31, 129.3, 128.7, 128.6, 126.8, 126.0, 123.6, 119.2, 84.5. **HRMS (ESI-TOF)**, *m/z* calcd for $C_{26}H_{18}NO_4$ $[M+H]^+$ 408.1230 found 408.1234.

(E)-9-Benzylidene-4-(4-methoxyphenyl)-7-phenyl-1-oxa-7-azaspiro[4.4]non-3-ene-2,6,8-trione (2i)

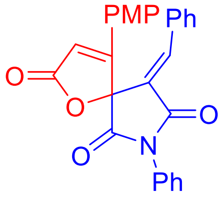

Prepared according to the general procedure **GP1** from diazo compound **1b** and 3-(4-methoxyphenyl)propiolic acid (scale – 0.5 mmol). Yield: 162 mg (77%). Eluent – *n*-hexane/acetone (from 10 to 40% of acetone). White solid, mp 188.6–189.9 °C. **^{1}H NMR (400 MHz, CDCl$_3$)**: δ 7.94 (d, *J* = 2.2 Hz, 1H), 7.74 – 7.67 (m, 2H), 7.53 (m, 7H), 7.46 (m, 3H), 6.96 – 6.83 (m, 2H), 6.26 (d, *J* = 2.2 Hz, 1H), 3.85 (s, 3H). **^{13}C{^{1}H} NMR (101 MHz, CDCl$_3$)**: δ 170.0, 167.9, 161.9, 152.7, 140.5, 135.3, 132.4, 131.7, 131.4, 131.0, 129.3, 129.2, 128.9, 126.5, 122.1, 114.4, 110.6, 90.6, 78.6, 68.9, 55.5. **HRMS (ESI-TOF)**, *m/z* calcd for $C_{27}H_{19}NNaO_5$ $[M+Na]^+$ 460.1155 found 460.1151.

(E)-9-Benzylidene-7-phenyl-4-(thiophen-2-yl)-1-oxa-7-azaspiro[4.4]non-3-ene-2,6,8-trione (2j)

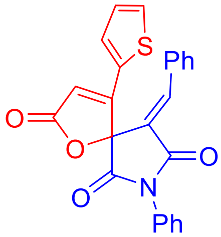

Prepared according to the general procedure **GP1** from corresponding diazo compound **1b** and 3-(thiophen-2-yl)propiolic acid (scale – 0.5 mmol). Yield: 138 mg (71%). Eluent – *n*-hexane/acetone (from 10 to 50% of acetone). White solid, mp 186.5 186.9 °C. **^{1}H**

NMR (400 MHz, $CDCl_3$): δ 8.31 (s, 1H), 7.58 – 7.51 (m, 3H), 7.50 – 7.45 (m, 1H), 7.46 – 7.40 (m, 3H), 7.35 (m, 4H), 7.24 (dd, J = 3.8, 1.1 Hz, 1H), 7.09 (dd, J = 5.1, 3.8 Hz, 1H), 6.42 (s, 1H). **$^{13}C\{^1H\}$ NMR (101 MHz, $CDCl_3$)**: δ 170.0, 168.5, 167.1, 155.5, 146.5, 132.1, 131.4, 131.3, 131.2, 130.5, 129.9, 129.4, 129.3, 128.8, 128.7, 126.0, 123.4, 115.6, 83.7. **HRMS (ESI-TOF)**, *m/z* calcd for $C_{24}H_{15}NNaO_4S$ $[M+Na]^+$ 436.0614 found 436.0613.

(E)-9-Benzylidene-7-phenyl-4-propyl-1-oxa-7-azaspiro[4.4]non-3-ene-2,6,8-trione (2k)

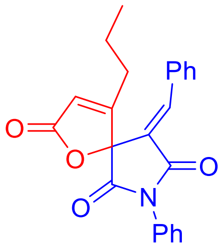

Prepared according to the general procedure **GP1** from diazo compound **1b** and hex-2-ynoic acid (scale – 1.0 mmol). Yield: 344 mg (66%). Eluent – *n*-hexane/acetone (from 0 to 30% of acetone). White amorphous solid. **^1H NMR (400 MHz, $CDCl_3$)**: δ 8.27 (s, 1H), 7.54 (t, J = 7.6 Hz, 2H), 7.43 (m, 8H), 6.08 (s, 1H), 2.01 (t, J = 7.5 Hz, 2H), 1.50 (dq, J = 14.6, 7.5 Hz, 1H), 1.37 (dq, J = 14.6, 7.5 Hz, 1H), 0.80 (t, J = 7.5 Hz, 3H. **$^{13}C\{^1H\}$ NMR (101 MHz, $CDCl_3$)**: δ 170.7, 168.8, 167.2, 166.9, 145.4, 132.0, 131.4, 131.3, 130.1, 129.4, 129.2, 128.9, 126.0, 122.5, 119.6, 85.5, 29.3, 19.7, 13.4. **HRMS (ESI-TOF)**, *m/z* calcd for $C_{23}H_{19}NNaO_4$ $[M+Na]^+$ 396.1206 found 396.1205.

General procedure (GP2) for the synthesis of compounds 3a–d

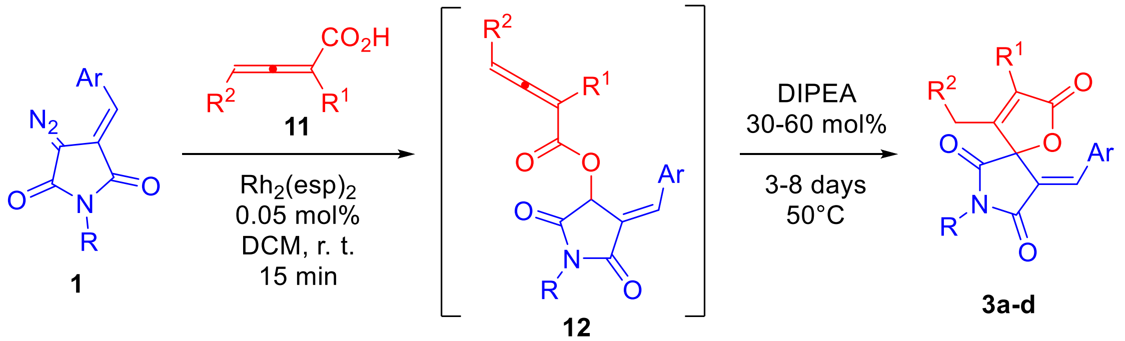

To a stirred solution of diazoarylidene succinimide **1** (0.5 mmol, 1 equiv) and allenic acid **11** (0.55 mmol, 1.1 equiv) in dry DCM (1.9 mL per 0.5 mmol of diazo compound **1**), a $Rh_2(esp)_2$ solution in dry DCM (100 µL per 0.5 mmol of diazo compound **1**, 2 mg/mL, 0.05 mol %) was added at room temperature. After stirring for 15 min, DIPEA (30 mol %) was added. The solution was stirred for 3 d at 50 °C and then directly purified by flash column chromatography on silica gel (*wet-loading* method, *n*-hexane/acetone) to give products **3a–c**.

In the case of **3d**, the solution was stirred with 30 mol % DIPEA for 5 d at 50 °C and then for another 3 d with an additional portion of DIPEA (30 mol %) at 50 °C. Subsequent flash column chromatography on silica gel (*wet-loading* method, *n*-hexane/acetone) afforded product **3d.**

(E)-7-Benzyl-9-benzylidene-3,4-dimethyl-1-oxa-7-azaspiro[4.4]non-3-ene-2,6,8-trione (3a)

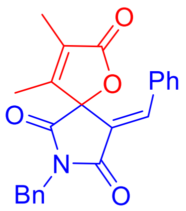

Prepared according to the general procedure **GP2** from diazo compound **1a** and 2-methylbuta-2,3-dienoic acid. Eluent – *n*-hexane/acetone (from 0 to 20% of acetone). Yield: 148 mg (87%). Light yellow oil. **^1H NMR ($CDCl_3$, 400 MHz)**: δ 8.11 (s, 1H), 7.43 – 7.38 (m, 3H), 7.37 – 7.29 (m, 5H), 7.24 – 7.19 (m, 2H), 4.95 – 4.76 (m, 2H), 1.70 (q, J = 1.1 Hz, 3H), 1.45 (q, J = 1.1 Hz, 3H). **$^{13}C\{^1H\}$ NMR ($CDCl_3$, 101 MHz)**: δ 172.4, 170.1, 167.9, 152.7, 144.1, 135.2, 132.1, 131.0, 129.6, 129.0, 128.8, 128.7, 128.5, 128.4, 123.6, 84.5, 43.6, 10.9, 8.9. **HRMS (ESI-TOF)**, *m/z* calcd for $C_{23}H_{20}NO_4$ $[M+H]^+$ 374.1387; found 374.1388.

(E)-7-Benzyl-9-benzylidene-3-ethyl-4-(4-methylbenzyl)-1-oxa-7-azaspiro[4.4]non-3-ene-2,6,8-trione (3b)

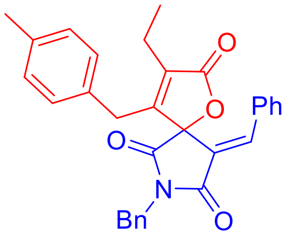

Prepared according to the general procedure **GP2** from diazo compound **1a** and 2-ethyl-4-(*p*-tolyl)buta-2,3-dienoic acid. Eluent – *n*-hexane/acetone (from 0 to 15% of acetone). Yield: 180 mg (80%). White solid; mp 144–146 °C. **^{1}H NMR ($CDCl_3$, 400 MHz)**: δ 7.98 (s, 1H), 7.43 – 7.37 (m, 1H), 7.37 – 7.32 (m, 2H), 7.31 – 7.27 (m, 7H), 6.97 (d, *J* = 7.6 Hz, 2H), 6.69 (d, *J* = 7.8 Hz, 2H), 4.39 (d, *J* = 14.0 Hz, 1H), 4.22 (d, *J* = 14.0 Hz, 1H), 3.47 (d, *J* = 15.8 Hz, 1H), 3.01 (d, *J* = 15.8 Hz, 1H), 2.39 – 2.25 (m, 2H), 2.29 (s, 3H), 0.99 (t, *J* = 7.6 Hz, 3H). **$^{13}C\{^1H\}$ NMR ($CDCl_3$, 101 MHz)**: δ 171.9, 169.9, 167.7, 154.8, 143.6, 137.4, 134.9, 134.3, 132.4, 131.1, 130.6, 130.2, 129.5, 129.5, 128.9, 128.9 (d, *J* = 0.4 Hz), 128.4, 123.5, 83.9, 43.3, 32.5, 21.1, 17.7, 12.5. **HRMS (ESI-TOF)**, *m/z* calcd for $C_{31}H_{27}NO_4Na$ $[M+Na]^+$ 500.1832; found 500.1837.

(E)-Methyl 4-((3,4-dimethyl-2,6,8-trioxo-7-phenyl-1-oxa-7-azaspiro[4.4]non-3-en-9-ylidene)methyl)benzoate (3c)

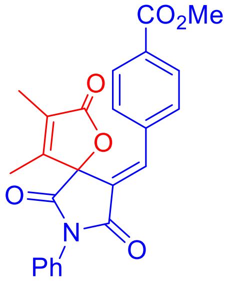

Prepared according to the general procedure **GP2** from diazo compound **1c** and 2-methylbuta-2,3-dienoic acid. Eluent – *n*-hexane/acetone (from 3 to 30% of acetone). Yield: 169 mg (83%). White solid; mp 220–222 °C. **^{1}H NMR ($CDCl_3$, 400 MHz)**: δ 8.25 (s, 1H), 8.03 (d, *J* = 8.3 Hz, 2H), 7.56 – 7.50 (m, 2H), 7.49 – 7.44 (m, 1H), 7.43 – 7.39 (m, 2H), 7.36 (d, *J* = 8.3 Hz, 2H), 3.94 (s, 3H), 1.74 (q, *J* = 1.1 Hz, 3H), 1.68 (q, *J* = 1.1 Hz, 3H). **$^{13}C\{^1H\}$ NMR ($CDCl_3$, 101 MHz)**: δ 172.1, 169.1, 166.9, 166.1, 152.4, 143.4, 136.3, 132.1, 131.3, 129.8, 129.5, 129.4, 129.4, 128.7, 126.1, 125.3, 84.2, 52.6, 11.2, 9.0. **HRMS (ESI-TOF)**, *m/z* calcd for $C_{24}H_{19}NO_6Na$ $[M+Na]^+$ 440.1105; found 440.1109.

(E)-7-cyclopropyl-3,4-dimethyl-9-(4-methylbenzylidene)-1-oxa-7-azaspiro[4.4]non-3-ene-2,6,8-trione (3d)

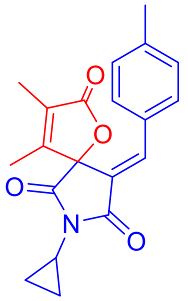

Prepared according to the general procedure **GP2** from diazo compound **1d** and 2-methylbuta-2,3-dienoic acid. Eluent – *n*-hexane/acetone (from 1 to 25% of acetone). Yield: 122 mg (78%). White solid; mp 130–132 °C. **^{1}H NMR ($CDCl_3$, 400 MHz)**: δ 8.04 (s, 1H), 7.16 – 7.10 (m, 4H), 2.89 – 2.82 (m, 1H), 2.35 (s, 3H), 1.77 (q, *J* = 1.1 Hz, 3H), 1.54 (q, *J* = 1.1 Hz, 3H), 1.15 – 0.99 (m, 4H). **$^{13}C\{^1H\}$ NMR ($CDCl_3$, 101 MHz)**: δ 172.5, 170.6, 169.0, 152.9, 143.8, 141.9, 130.2, 129.5, 129.4, 128.5, 121.8, 84.3, 23.6, 21.6, 11.1, 9.0, 5.4, 5.3. **HRMS (ESI-TOF)**, *m/z* calcd for $C_{20}H_{19}NO_4Na$ $[M+Na]^+$ 360.1206; found 360.1202.

General procedure (GP3) for the synthesis of compounds 4

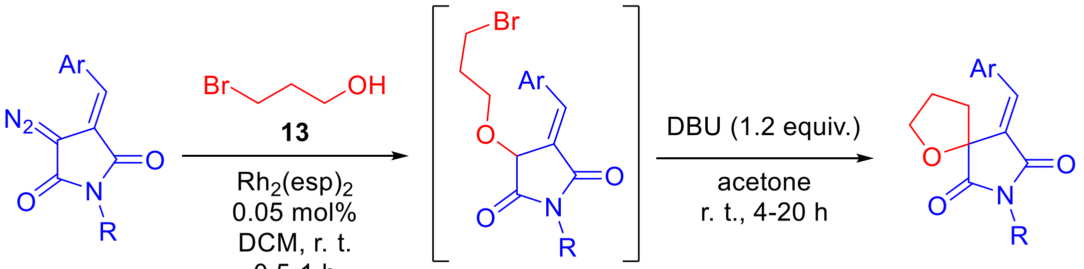

1 **14** **4a-d**

To a stirred solution of 3-bromopropan-1-ol (83 mg, 0.6 mmol, 1.2 equiv) in dry DCM (0.8 mL) was added the catalyst solution (200 µL of 2.5 mM $Rh_2(esp)_2$ in DCM, 0.05 mol %) followed by dropwise addition of the solution of diazo compound **1** (1.0 mmol, 1.0 equiv) in dry DCM (1 mL). The reaction mixture was

stirred at ambient temperature for 0.5–1 h (controlled by TLC). DCM was evaporated and the residue was dissolved in dry acetone (2 mL) followed by the addition of DBU (91 mg, 0.6 mmol, 1.2 equiv). The mixture was stirred at room temperature for 1–3 d (controlled by TLC). The reaction mixture was diluted with *n*-hexane (2 mL) and the resulting solution was subjected to column chromatography on silica gel (eluent – *n*-hexane/acetone, from 5 to 25% of acetone) to afford pure compounds **4**.

(E)-9-Benzylidene-7-phenyl-1-oxa-7-azaspiro[4.4]nonane-6,8-dione (4a)

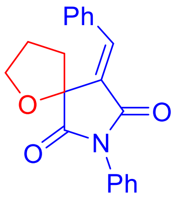

Prepared according to the general procedure **GP3** from diazo compound **1b** and 3-bromopropan-1-ol (scale – 0.5 mmol). Yield: 109 mg (68%) White solid, mp 149.5–150.8 °C. **^{1}H NMR (400 MHz, CDCl$_3$)**: δ 8.01 (s, 1H), 7.82 – 7.71 (m, 2H), 7.56 – 7.49 (m, 2H), 7.49 – 7.36 (m, 6H), 4.54 – 4.32 (m, 2H), 2.67 – 2.42 (m, 1H), 2.30 – 2.05 (m, 3H). **^{13}C{^{1}H} NMR (101 MHz, CDCl$_3$)**: δ 176.6, 168.3, 140.8, 133.4, 131.7, 131.1, 130.4, 129.1, 128.6, 128.5, 128.2, 126.4, 81.8, 70.0, 32.2, 26.1. **HRMS (ESI-TOF)**, *m/z* calcd for $C_{20}H_{17}NNaO_3$ $[M+Na]^+$ 342.1101 found 342.1097.

(E)-9-Benzylidene-7-(p-tolyl)-1-oxa-7-azaspiro[4.4]nonane-6,8-dione (4b)

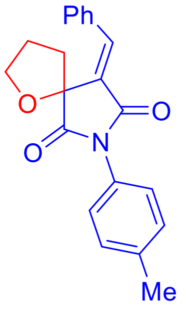

Prepared according to the general procedure **GP3** from diazo compound **1e** and 3-bromopropan-1-ol (scale – 0.5 mmol). Yield: 86 mg (57%) White solid, mp 156.4–157.8 °C. **^{1}H NMR (400 MHz, CDCl$_3$)**: δ 8.00 (s, 1H), 7.83 – 7.70 (m, 2H), 7.52 – 7.40 (m, 3H), 7.31 (s, 4H), 4.56 – 4.30 (m, 2H), 2.50 (m, 1H), 2.42 (s, 3H), 2.31 – 2.04 (m, 3H). **^{13}C{^{1}H} NMR (101 MHz, CDCl$_3$)**: δ 176.7, 168.5, 140.6, 138.6, 133.5, 131.1, 130.4, 129.3, 129.1, 128.6, 128.3, 126.2, 81.8, 70.0, 32.2, 26.1, 21.3. **HRMS (ESI-TOF)**, *m/z* calcd for $C_{21}H_{19}NNaO_3$ $[M+Na]^+$ 356.1257 found 356.1260.

(E)-9-Benzylidene-7-(4-(trifluoromethyl)phenyl)-1-oxa-7-azaspiro[4.4]nonane-6,8-dione (4c)

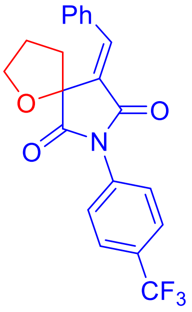

Prepared according to the general procedure **GP3** from diazo compound **1f** and 3-bromopropan-1-ol (scale – 0.5 mmol). Yield: 87 mg (45%) White solid, mp 164.7–164.9 °C. **^{1}H NMR (400 MHz, CDCl$_3$)**: δ 8.03 (s, 1H), 7.83 – 7.70 (m, 4H), 7.63 (d, J = 8.3 Hz, 2H), 7.52 – 7.43 (m, 3H), 4.52 – 4.34 (m, 2H), 2.51 (m, 1H), 2.31 – 2.06 (m, 3H). **^{13}C{^{1}H} NMR (101 MHz, CDCl$_3$)**: δ 176.2, 167.9, 141.6, 134.8, 133.2, 131.2, 130.6, 130.4 (q, $J_{C\text{-}F}$ = 32.9 Hz), 128.7, 127.6, 126.6, 126.2 (q, $J_{C\text{-}F}$ = 3.7 Hz), 123.7 (q, $J_{C\text{-}F}$ = 272.4 Hz), 81.8, 70.1, 32.2, 26.1. **^{19}F{^{1}H} NMR (376 MHz, CDCl$_3$)**: δ -62.69. **HRMS (ESI-TOF)**, *m/z* calcd for $C_{21}H_{17}F_3NO_3$ $[M+H]^+$ 388.1155 found 388.1155.

(E)-Methyl 4-((6,8-dioxo-7-phenyl-1-oxa-7-azaspiro[4.4]nonan-9-ylidene)methyl)benzoate (4d)

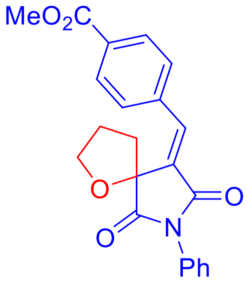

Prepared according to the general procedure **GP3** from diazo compound **1c** and 3-bromopropan-1-ol (scale – 0.7 mmol). Yield: 90 mg (34%) White amorphous solid. **^{1}H NMR (400 MHz, CDCl$_3$)**: δ 8.13 (d, J = 8.4 Hz, 2H), 8.01 (s, 1H), 7.81 (d, J = 8.4 Hz, 2H), 7.55 – 7.51 (m, 2H), 7.48 – 7.40 (m, 3H), 4.54 – 4.29 (m, 2H), 3.98 (s, 3H), 2.61 – 2.42 (m, 1H), 2.37 – 2.20 (m, 1H), 2.19 – 1.98 (m, 2H). **^{13}C{^{1}H} NMR (101 MHz, CDCl$_3$)**: δ 176.3, 167.9, 166.4, 139.3, 137.8, 131.5, 131.3, 130.8, 130.6, 129.7, 129.2, 128.7, 126.3, 81.6, 70.2, 52.4, 32.8, 26.2. **HRMS (ESI-TOF)**, *m/z* calcd for $C_{22}H_{20}NO_5$ $[M+H]^+$ 378.1336 found 378.1338.

General procedure (GP4) for the synthesis of compounds 5, 16, 18, 21, 24, and 25

Step 1: To a stirred solution of the corresponding OH substrate (0.6 mmol, 1.2 equiv) in dry DCM (0.8 mL) was added the catalyst solution (200 µL of 2.5 mM $Rh_2(esp)_2$ in DCM, 0.05 mol %) followed by dropwise addition of the solution of diazo compound **1** (0.5 mmol, 1.0 equiv) in dry DCM (1 mL). The reaction mixture was stirred at ambient temperature for 0.5–1 h (controlled by TLC), diluted with *n*-hexane and subjected to flash column chromatography (Eluent – *n*-hexane/DCM, 1:1) to afford compound **16**, which was pure enough for the next step.

Step 2: The obtained substance (0.2 mmol) was dissolved in dry acetone (1 mL) followed by the addition of DBU (36 mg, 0.24 mmol, 1.2 equiv). The mixture was stirred at room temperature for 15–20 min (controlled by TLC). The reaction mixture was diluted with *n*-hexane (1 mL) and the resulting solution was subjected to column chromatography on silica gel (eluent – *n*-hexane/acetone, from 5 to 25% of acetone) to afford pure title compounds.

(E)-3-Benzylidene-4-(2-(bromomethyl)phenoxy)-1-phenylpyrrolidine-2,5-dione (16a)

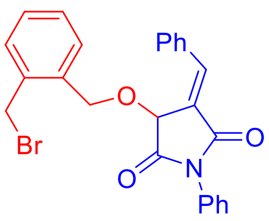

Prepared according to the general procedure **GP4** (Step 1) from diazo compound **1b** and (2-(bromomethyl)phenyl)methanol (scale – 0.5 mmol).[2] Yield: 162 mg (72%). White amorphous solid. **^{1}H NMR (400 MHz, $CDCl_3$)**: δ 8.00 (d, *J* = 2.0 Hz, 1H), 7.72 (dd, *J* = 8.0, 1.7 Hz, 2H), 7.58 – 7.50 (m, 3H), 7.50 – 7.43 (m, 3H), 7.40 (dt, *J* = 8.0, 1.7 Hz, 3H), 7.37 – 7.29 (m, 3H), 5.33 (d, *J* = 2.0 Hz, 1H), 5.19 (d, *J* = 11.0 Hz, 1H), 4.93 (d, *J* = 11.0 Hz, 1H), 4.58 (d, *J* = 10.3 Hz, 1H), 4.56 (d, *J* = 10.3 Hz, 1H).

(E)-4'-Benzylidene-1'-phenylspiro[isochroman-3,3'-pyrrolidine]-2',5'-dione (5a)

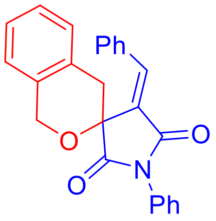

Prepared according to the general procedure **GP4** (Step 2) from compound **16a** (scale – 0.2 mmol). Yield: 80 mg (87%) White solid, mp 156.7–156.9 °C. **^{1}H NMR (400 MHz, $CDCl_3$)**: δ 8.05 (s, 1H), 7.97 – 7.88 (m, 2H), 7.57 – 7.33 (m, 8H), 7.32 – 7.26 (m, 1H), 7.22 (m, 1H), 7.17 (d, *J* = 7.5 Hz, 1H), 7.07 (d, *J* = 7.5 Hz, 1H), 5.53 (d, *J* = 15.1 Hz, 1H), 5.20 (d, *J* = 15.1 Hz, 1H), 3.62 (d, *J* = 17.5 Hz, 1H), 2.99 (d, *J* = 17.5 Hz, 1H). **^{13}C{^{1}H} NMR (101 MHz, $CDCl_3$)**: δ 173.2, 168.1, 141.1, 133.3, 132.7, 132.2, 131.5, 130.8, 129.7, 129.0, 128.8, 128.8, 128.6, 128.6, 126.86, 126.5, 126.4, 124.1, 73.3, 65.8, 29.9. **HRMS (ESI-TOF)**, *m/z* calcd for $C_{25}H_{20}NO_3$ $[M+H]^+$ 382.1438 found 382.1445.

(E)-Methyl 4-((4-((2-(bromomethyl)benzyl)oxy)-2,5-dioxo-1-phenylpyrrolidin-3-ylidene)methyl)benzoate (16b)

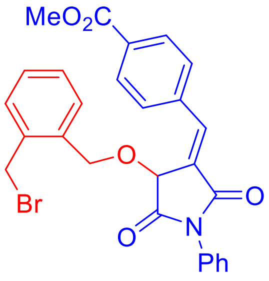

Prepared according to the general procedure **GP4** (Step 1) from diazo compound **1c** and (2-(bromomethyl)phenyl)methanol (scale – 0.5 mmol). Yield: 182 mg (70%) White amorphous solid. **^{1}H NMR (400 MHz, $CDCl_3$)**: δ 8.11 – 8.08 (d, *J* = 8.3 Hz, 2H), 8.00 (d, *J* = 2.0 Hz, 1H), 7.76 (d, *J* = 8.3 Hz, 2H), 7.54 (m, 2H), 7.50 – 7.43 (m, 1H), 7.42 – 7.39 (m, 3H), 7.35 (m, 1H), 7.32 – 7.29 (m, 2H), 5.31 (d, *J* = 2.0 Hz, 1H), 5.22 (d, *J* = 11.1 Hz, 1H), 4.94 (d, *J* = 11.1 Hz, 1H), 4.57 (d, *J* = 10.4 Hz, 1H), 4.53 (d, *J* = 10.4 Hz, 1H), 3.99 (s, 3H).

(E)-Methyl 4-((2',5'-dioxo-1'-phenylspiro[isochroman-3,3'-pyrrolidin]-4'-ylidene)methyl)benzoate (5b)

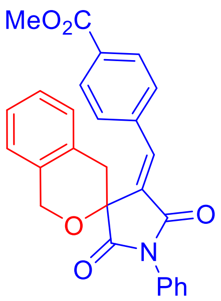

Prepared according to the general procedure **GP4** (Step 2) from compound **16b** (scale – 0.2 mmol). Yield: 83 mg (82%) White solid, mp 189.2–189.9 °C. **^{1}H NMR (400 MHz, $CDCl_3$)**: δ 8.08 – 7.97 (m, 3H), 7.93 (m, 2H), 7.55 – 7.48 (m, 2H), 7.48 – 7.39 (m, 2H),

7.27 (t, *J* = 6.6 Hz, 1H), 7.21 (d, *J* = 7.5 Hz, 1H), 7.16 (t, *J* = 6.6 Hz, 1H), 7.03 (d, *J* = 7.5 Hz, 1H), 5.50 (d, *J* = 15.0 Hz, 1H), 5.16 (d, *J* = 15.0 Hz, 1H), 3.92 (s, 3H), 3.48 (d, *J* = 17.3 Hz, 1H), 3.01 (d, *J* = 17.4 Hz, 1H). **^{13}C{^{1}H} NMR (101 MHz, CDCl$_3$)**: δ 172.9, 167.7, 166.3, 139.5, 136.9, 133.2, 131.7, 131.5, 131.4, 131.1, 129.8, 129.4, 129.1, 128.7, 128.4, 127.0, 126.6, 126.3, 124.1, 73.3, 65.9, 52.4, 30.2. **HRMS (ESI-TOF)**, *m/z* calcd for $C_{27}H_{21}NNaO_5$ [M+Na]$^+$ 462.1312 found 462.1313.

(E)-3-((2-(Bromomethyl)benzyl)oxy)-4-(2-methoxybenzylidene)-1-phenylpyrrolidine-2,5-dione (16c)

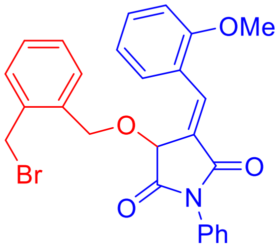

Prepared according to the general procedure **GP4** (Step 1) from diazo compound **1g** and (2-(bromomethyl)phenyl)methanol (scale – 1.0 mmol). Yield: 170 mg (69%). White amorphous solid. **^{1}H NMR (400 MHz, CDCl$_3$)**: δ 8.39 (d, *J* = 2.0 Hz, 1H), 7.69 (dd, *J* = 7.9, 1.7 Hz, 1H), 7.57 – 7.48 (m, 2H), 7.49 – 7.44 (m, 2H), 7.43 – 7.39 (m, 2H), 7.39 – 7.27 (m, 4H), 7.29 – 7.19 (m, 3H), 7.03 – 6.95 (m, 2H), 5.30 (d, *J* = 2.1 Hz, 1H), 5.14 (d, *J* = 10.9 Hz, 1H), 4.84 (d, *J* = 10.9 Hz, 1H), 4.49 (d, *J* = 10.4 Hz, 1H), 4.45 (d, *J* = 10.4 Hz, 1H), 3.93 (s, 3H).

(E)-4'-(2-Methoxybenzylidene)-1'-phenylspiro[isochroman-3,3'-pyrrolidine]-2',5'-dione (5c)

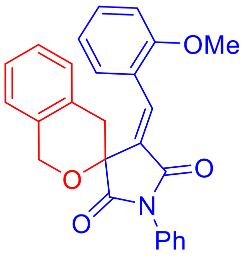

Prepared according to the general procedure **GP4** (Step 2) from compound **16c** (scale – 0.6 mmol). Yield: 100 mg (44%). White solid, mp 161.4–162.7 °C. **^{1}H NMR (400 MHz, CDCl$_3$)**: δ 8.45 (s, 1H), 8.11 (dd, *J* = 7.6, 1.6 Hz, 1H), 7.53 – 7.33 (m, 7H), 7.27 – 7.21 (m, 1H), 7.17 (td, *J* = 7.6, 1.6 Hz, 1H), 7.12 (d, *J* = 7.5 Hz, 1H), 7.01 (d, *J* = 7.5 Hz, 1H), 6.97 – 6.91 (m, 1H), 6.80 (t, *J* = 7.6 Hz, 1H), 5.42 (d, *J* = 15.0 Hz, 1H), 5.13 (d, *J* = 15.0 Hz, 1H), 3.94 (s, 3H), 3.52 (d, *J* = 17.4 Hz, 1H), 2.94 (d, *J* = 17.4 Hz, 1H). **^{13}C{^{1}H} NMR (101 MHz, CDCl$_3$)**: δ 173.2, 168.2, 158.9, 136.5, 133.4, 132.4, 132.5, 130.2, 128.9, 128.5, 128.5, 128.4, 128.1, 126.7, 126.4, 126.2, 123.9, 121.8, 120.6, 110.6, 73.3, 65.8, 55.7, 29.7. **HRMS (ESI-TOF)**, *m/z* calcd for $C_{26}H_{22}NO_4$ [M+H]$^+$ 412.1543 found 412.1555.

(E)-4-Benzylidene-2,5-dioxo-1-phenylpyrrolidin-3-yl 2-(bromomethyl)benzoate (18)

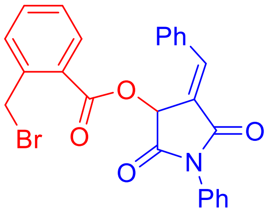

Prepared according to the general procedure **GP4** (Step 1) from diazo compound **1b** and 2-(bromomethyl)benzoic acid (scale – 0.5 mmol).[3] Yield: 202 mg (85%). White amorphous solid. **^{1}H NMR (400 MHz, CDCl$_3$)**: δ 7.95 (d, *J* = 2.2 Hz, 1H), 7.74 (dd, *J* = 7.9, 1.3 Hz, 1H), 7.70 – 7.66 (m, 2H), 7.57 – 7.41 (m, 10H), 7.29 (ddd, *J* = 7.8, 6.9, 1.8 Hz, 2H), 6.46 (d, *J* = 2.2 Hz, 1H), 4.93 (d, *J* = 10.0 Hz, 1H), 4.80 (d, *J* = 10.1 Hz, 1H). **^{13}C{^{1}H} NMR (101 MHz, CDCl$_3$)**: δ 170.5, 167.9, 165.2, 140.0, 139.9, 133.3, 132.5, 131.8, 131.7, 131.3, 131.2, 130.8, 129.23, 129.18, 128.8, 128.5, 127.1, 126.5, 122.8, 68.5, 30.8. **HRMS (ESI-TOF)**, *m/z* calcd for $C_{25}H_{18}BrNNaO_4$ [M+Na]$^+$ 498.0311/500.0291 found 498.0288/500.0287.

(E)-3-Benzylidene-4-(2-bromoethoxy)-1-phenylpyrrolidine-2,5-dione (21)

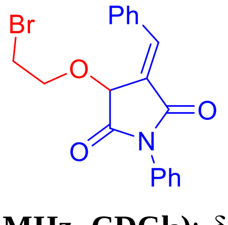

Prepared according to the general procedure **GP4** (Step 1) from diazo compound **1b** and 2-bromoethanol (scale – 0.5 mmol). Yield: 143 mg (74%). White solid, mp 148.5–149.3 °C. **^{1}H NMR (400 MHz, CDCl$_3$)**: δ 8.00 (d, *J* = 1.9 Hz, 1H), 7.88 – 7.77 (m, 2H), 7.61 – 7.49 (m, 5H), 7.48 – 7.35 (m, 3H), 5.12 (d, *J* = 1.9 Hz, 1H), 4.47 (dt, *J* = 10.8, 5.5 Hz, 1H), 4.09 (ddd, *J* = 10.5, 7.0, 5.6 Hz, 1H), 3.61 – 3.48 (m, 2H). **^{13}C{^{1}H} NMR (101 MHz, CDCl$_3$)**: δ 172.3, 168.3, 141.9, 132.9, 131.5, 131.44, 131.38, 129.19, 129.17, 128.8, 126.3, 123.2, 72.9, 69.1, 29.7. **HRMS (ESI-TOF)**, *m/z* calcd for $C_{19}H_{16}BrNNaO_3$ [M+Na]$^+$ 408.0206/410.0185 found 408.0203/410.0185.

(E)*-3-Benzylidene-4-(2-(2-bromoethoxy)ethoxy)-1-phenylpyrrolidine-2,5-dione (24)

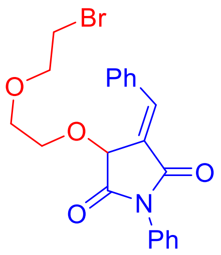

Prepared according to the general procedure **GP4** (Step 1) from diazo compound **1b** and 2-(2-bromoethoxy)ethan-1-ol (scale – 0.5 mmol).[4] Yield: 136 mg (63%). White amorphous solid. **^{1}H NMR (400 MHz, $CDCl_3$)**: δ 7.97 (d, *J* = 1.9 Hz, 1H), 7.82 (dd, *J* = 6.7, 3.0 Hz, 2H), 7.60 – 7.48 (m, 5H), 7.47 – 7.36 (m, 3H), 5.11 (d, *J* = 1.9 Hz, 1H), 4.26 (dt, *J* = 10.5, 3.9 Hz, 1H), 3.99 (dt, *J* = 10.4, 5.1 Hz, 1H), 3.85 – 3.69 (m, 4H), 3.40 (t, *J* = 6.3 Hz, 2H).

3-Benzyl-4-(2-(2-bromoethoxy)ethoxy)-1-phenyl-1H-pyrrole-2,5-dione (25)

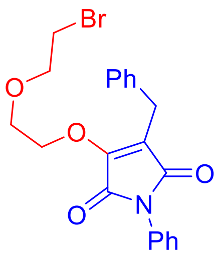

Prepared according to the general procedure **GP4** (Step 2) from compound **24** (scale – 0.2 mmol). Yield: 75 mg (87%). White amorphous solid. **^{1}H NMR (400 MHz, $CDCl_3$)**: δ 7.48 – 7.42 (m, 2H), 7.40 – 7.30 (m, 7H), 7.28 – 7.21 (m, 1H), 4.80 – 4.74 (m, 2H), 3.82 – 3.76 (m, 6H), 3.44 (t, *J* = 6.0 Hz, 2H). **^{13}C{^{1}H} NMR (101 MHz, $CDCl_3$)**: δ 169.8, 165.3, 152.4, 138.0, 131.3, 129.0, 128.8, 128.7, 127.5, 126.7, 126.0, 115.8, 71.2, 71.0, 70.1, 30.2, 27.5. **HRMS (ESI-TOF)**, *m/z* calcd for $C_{21}H_{20}BrNNaO_4$ $[M+Na]^+$ 452.0468/453.0447 found 452.0463/453.0445.

III. NMR spectra

Copies of ^{1}H (400.13 MHz, $CDCl_3$) and $^{13}C\{^{1}H\}$ (100.61 MHz, $CDCl_3$) spectra of **2a**

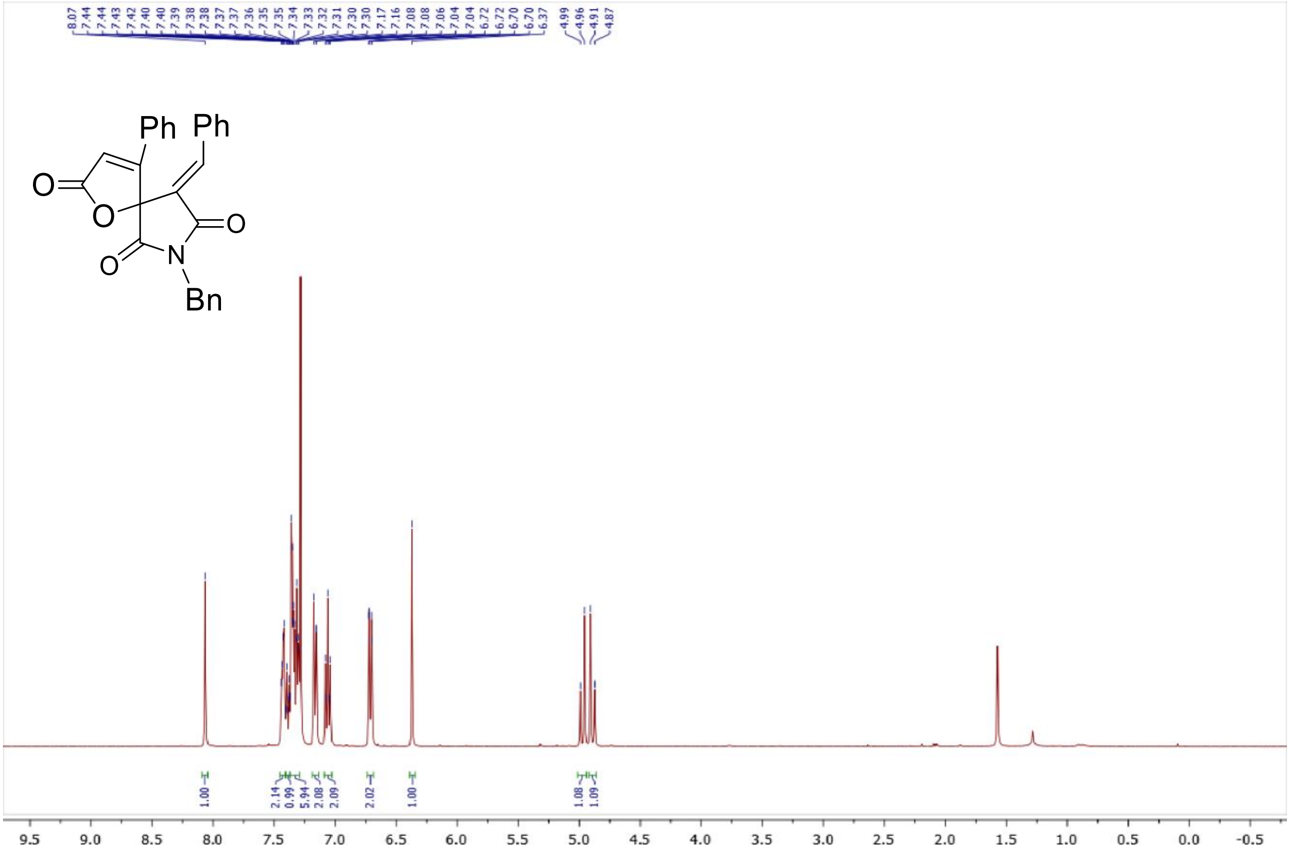

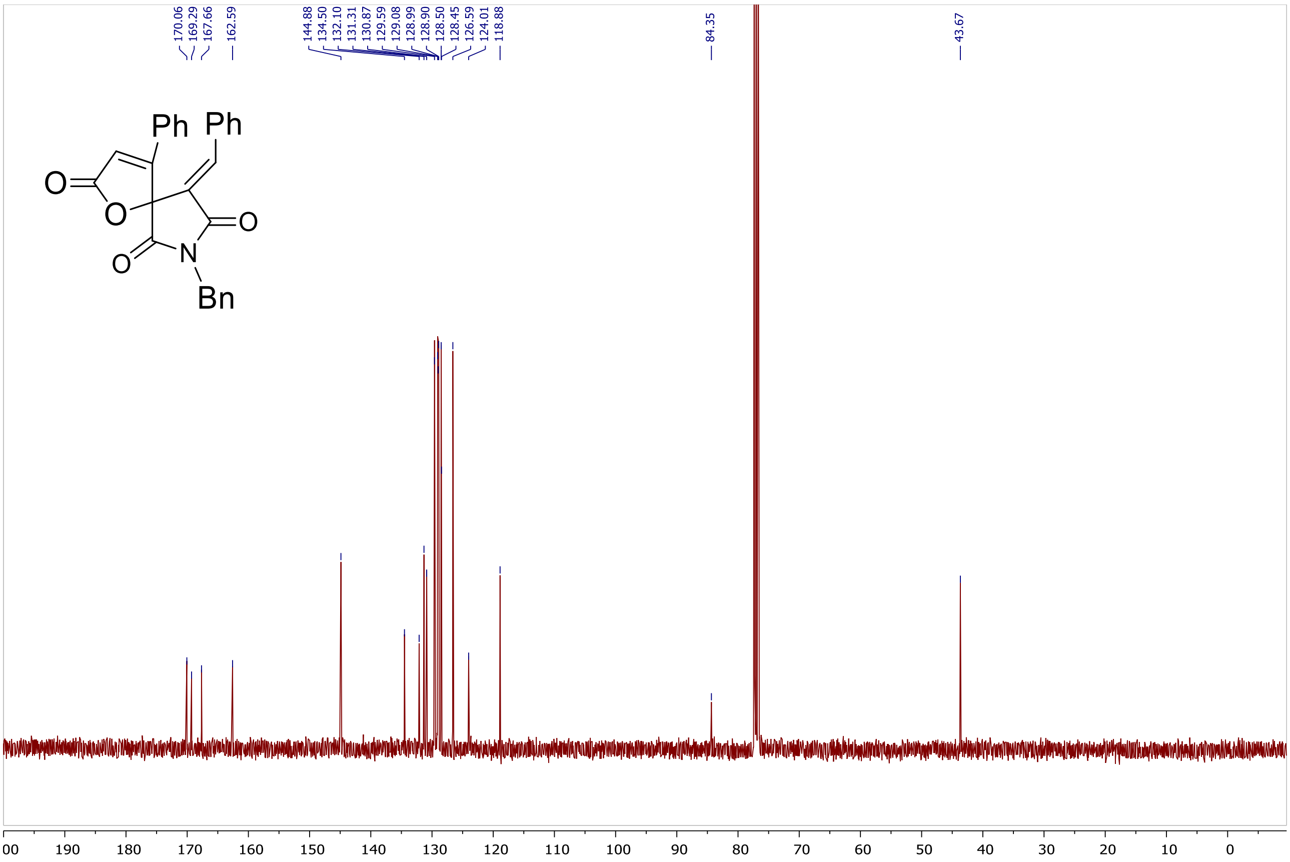

Copies of ^{1}H (400.13 MHz, $CDCl_3$) and $^{13}C\{^{1}H\}$ (100.61 MHz, $CDCl_3$) spectra of **2b**

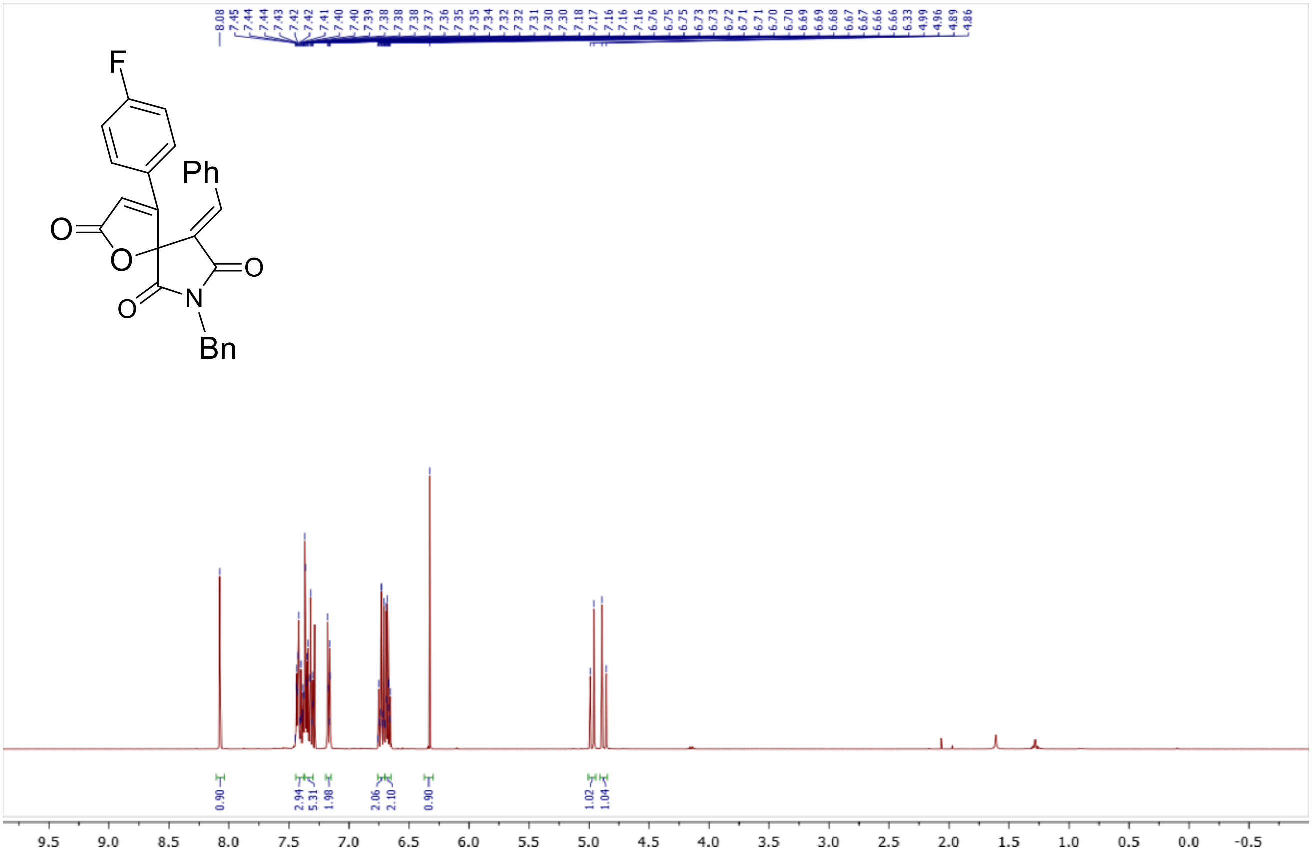

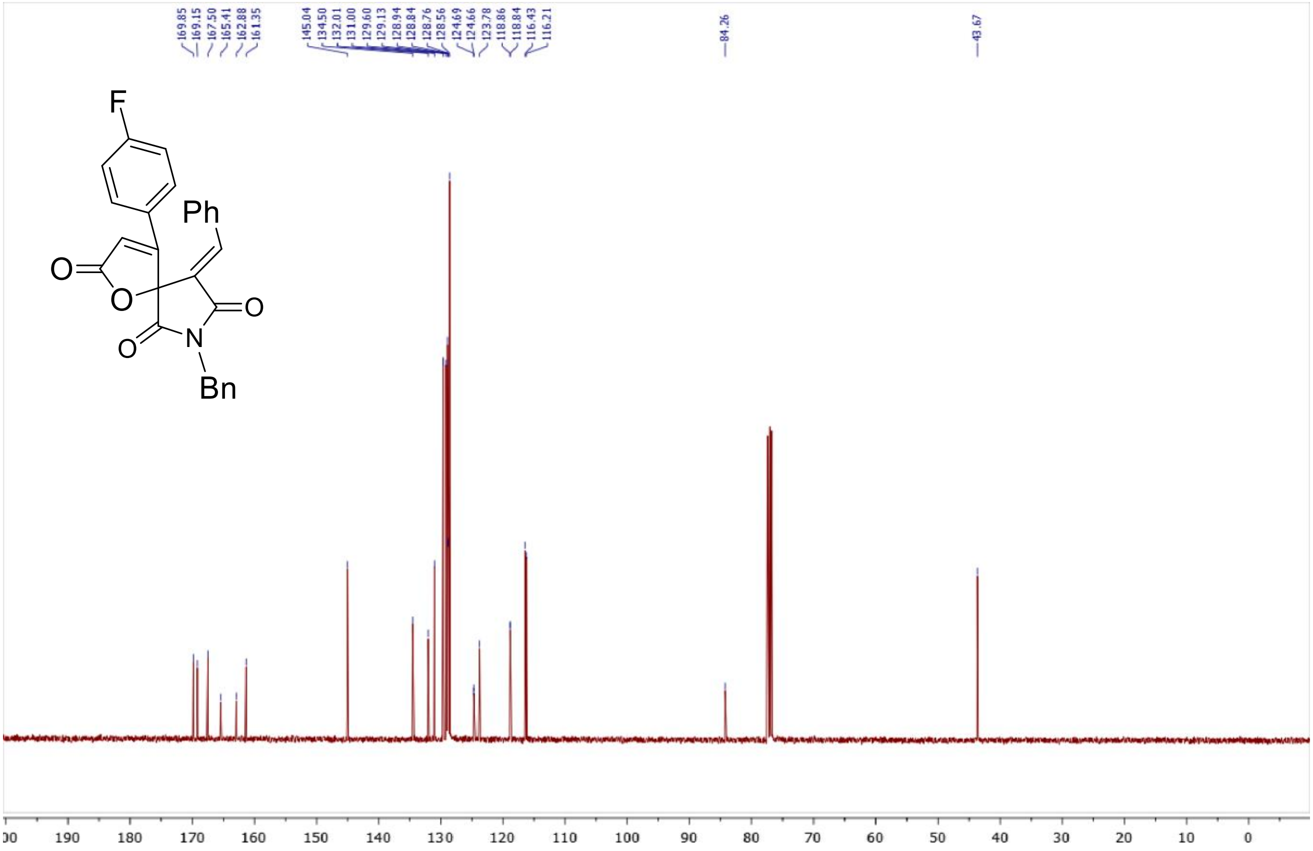

Copy of $^{19}F\{^1H\}$ (376.50 MHz, $CDCl_3$) spectrum of **2b**

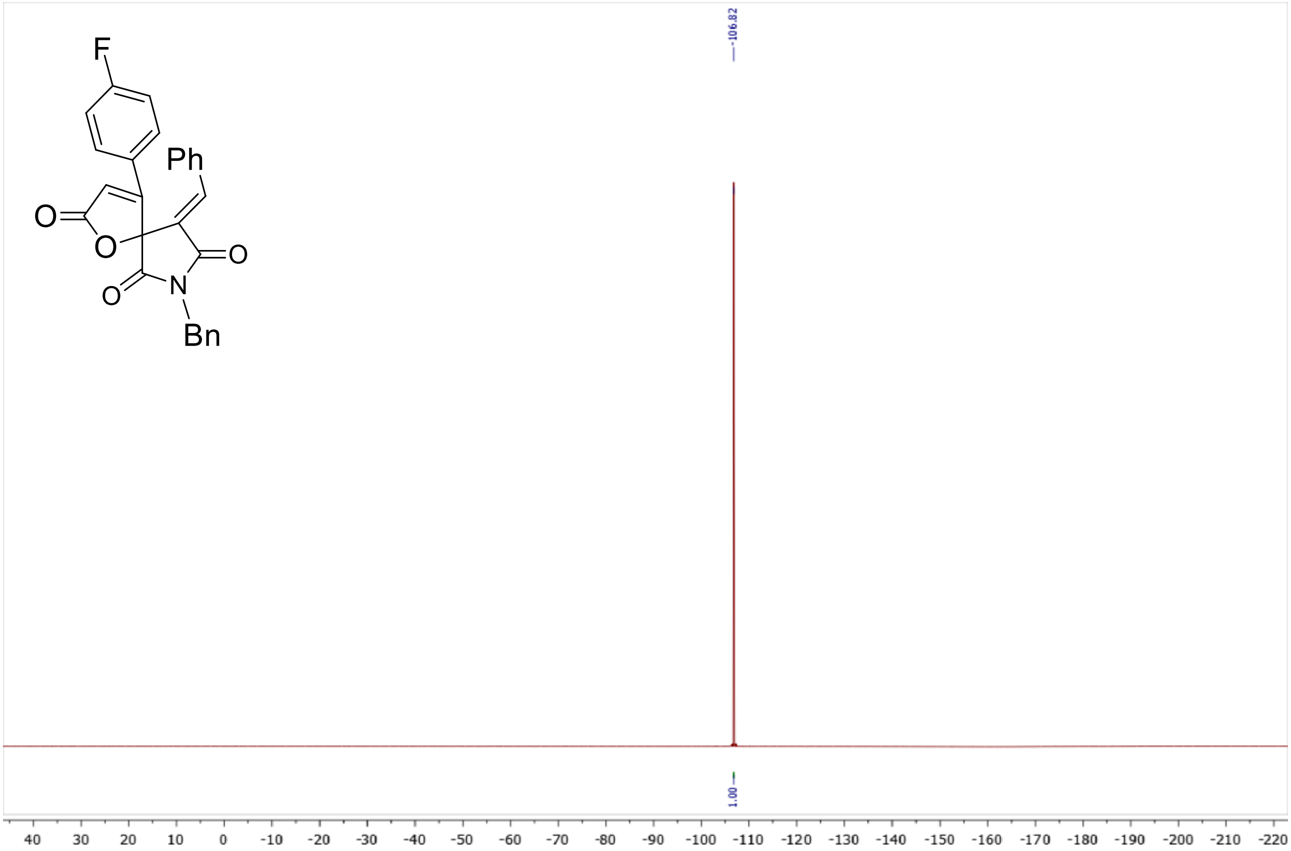

Copies of ^{1}H (400.13 MHz, $CDCl_3$) and $^{13}C\{^{1}H\}$ (100.61 MHz, $CDCl_3$) spectra of **2c**

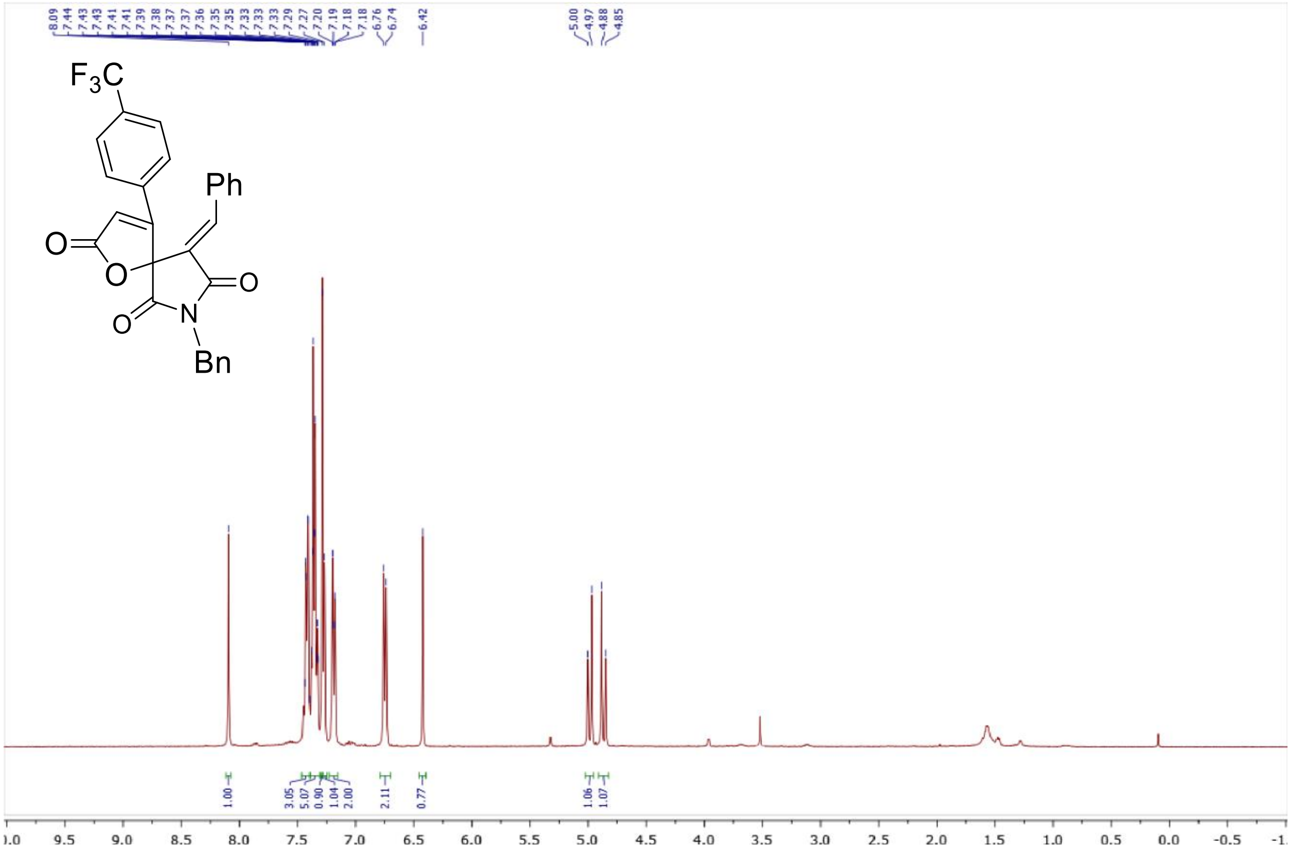

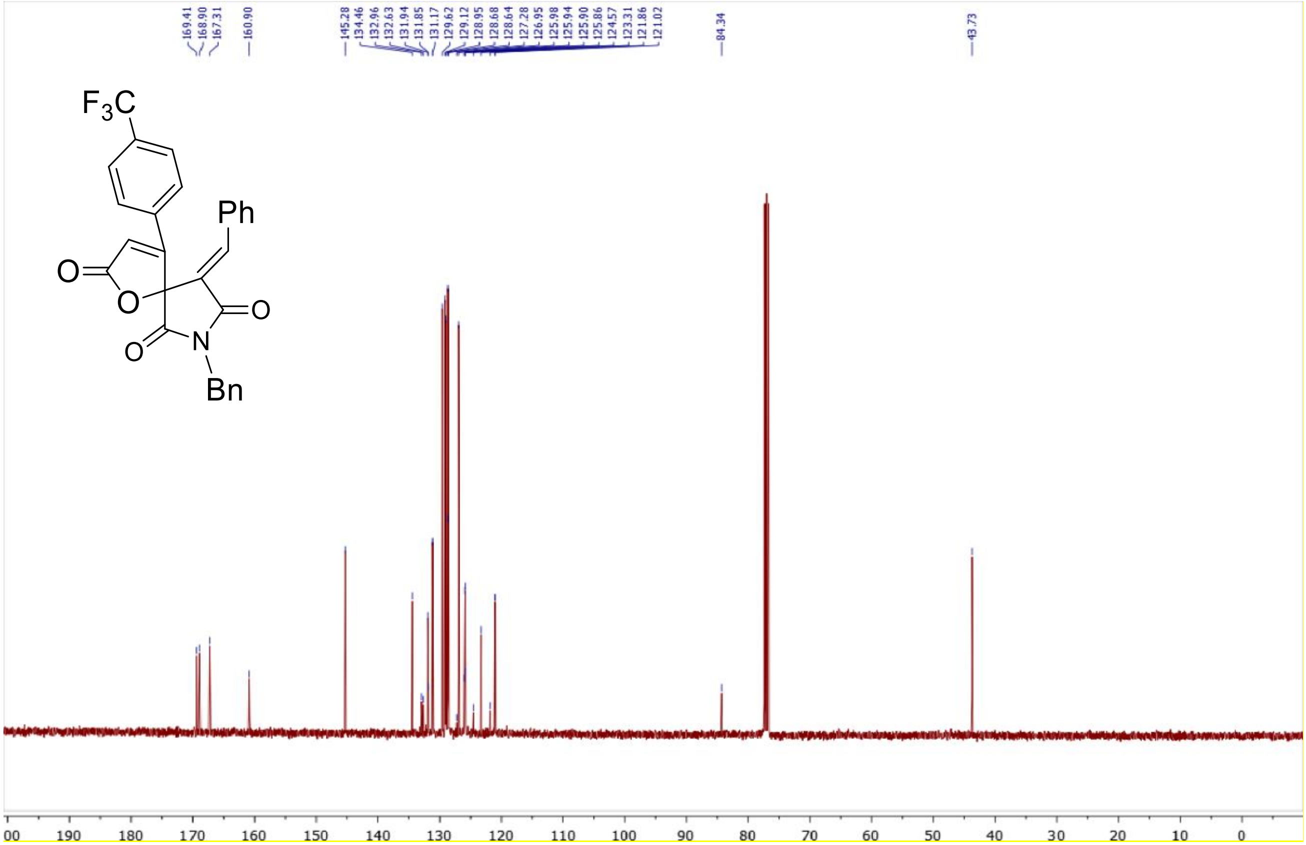

Copy of $^{19}F\{^{1}H\}$ (376.50 MHz, $CDCl_3$) spectrum of **2c**

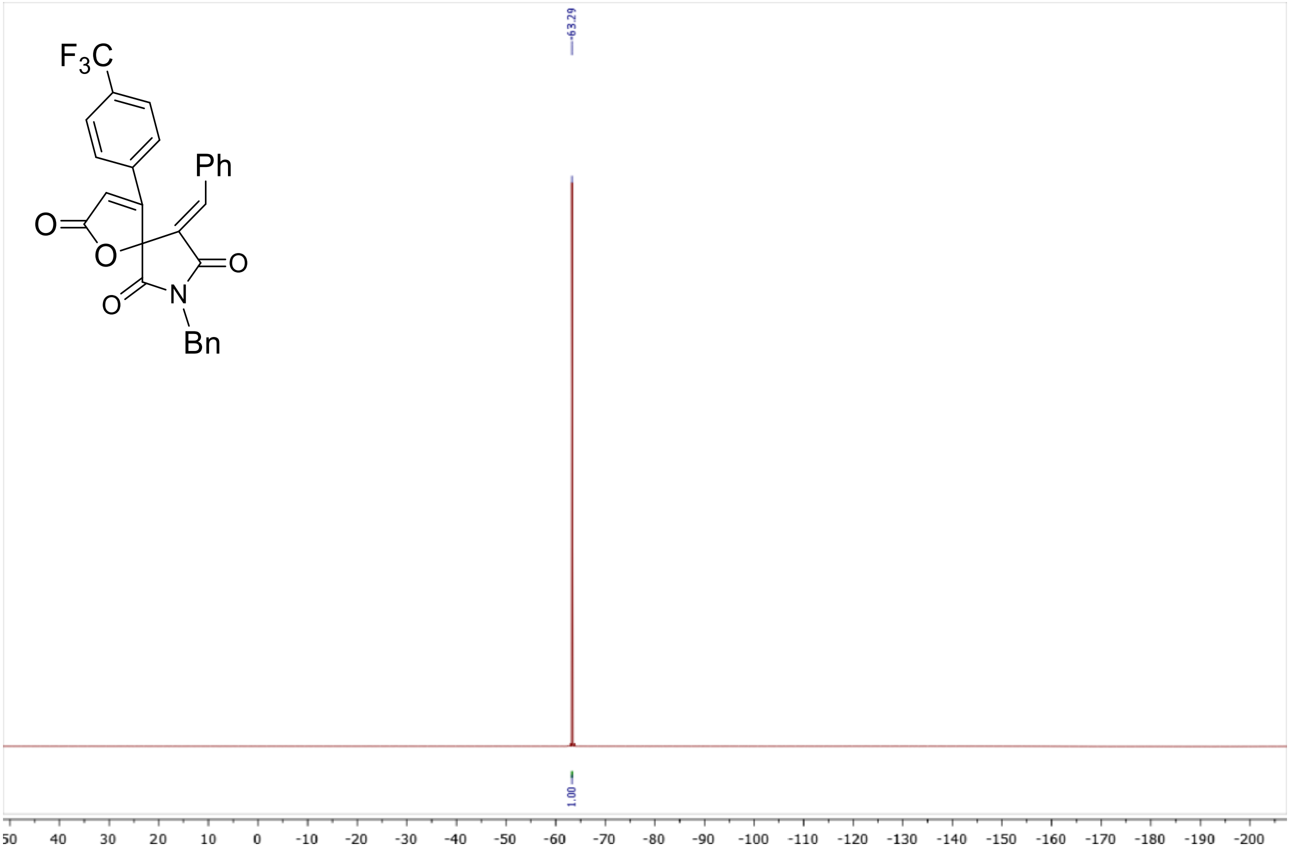

Copies of ^{1}H (400.13 MHz, $CDCl_3$) and ^{13}C{^{1}H} (100.61 MHz, $CDCl_3$) spectra of **2d**

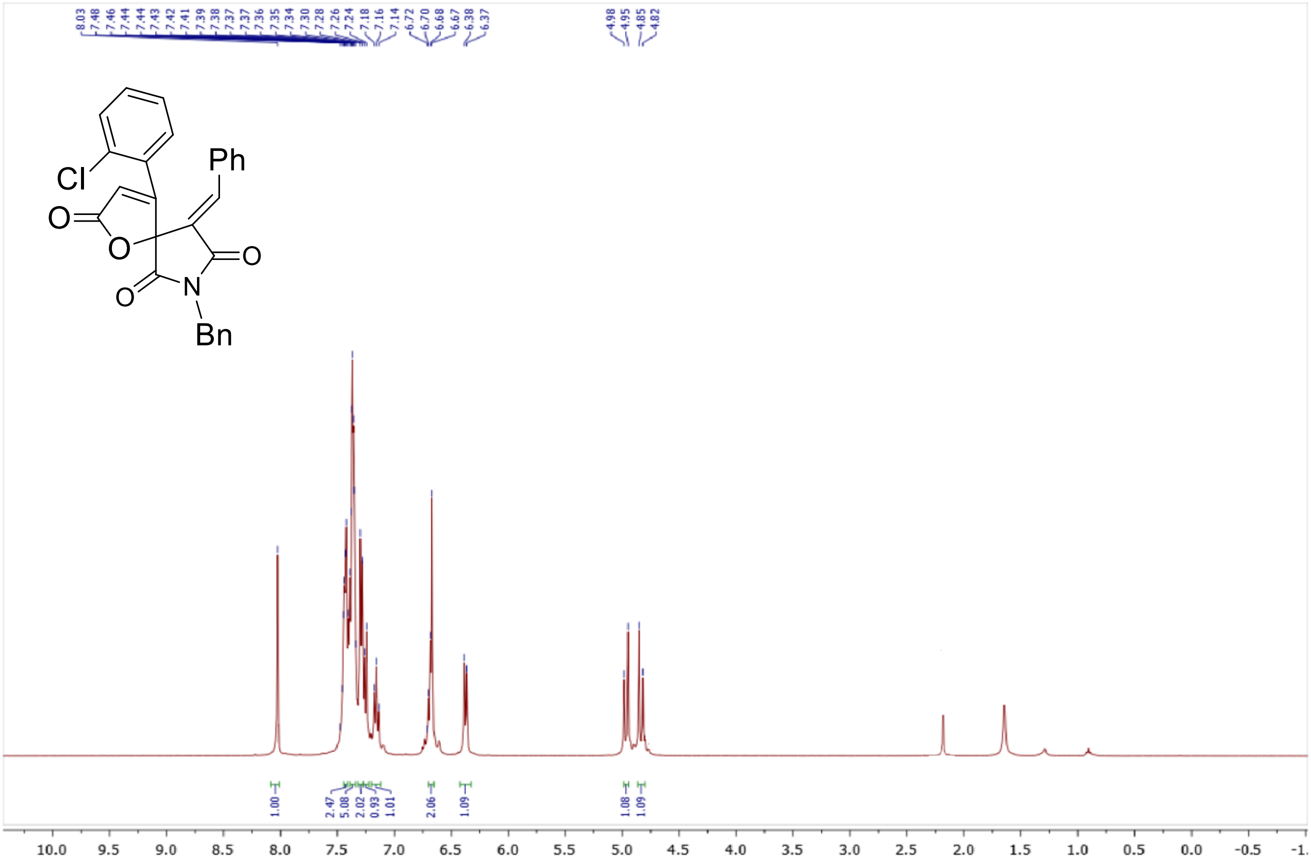

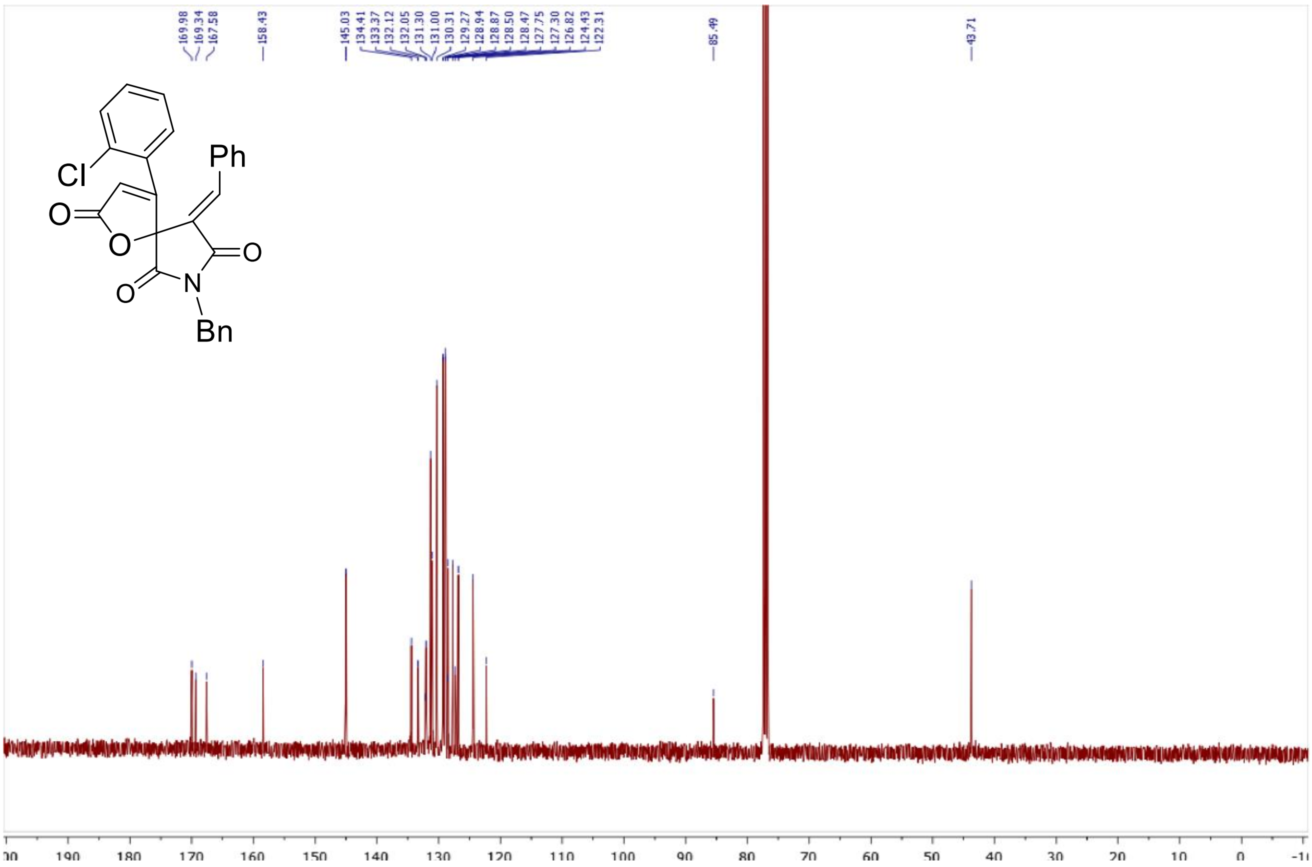

Copies of ^{1}H (400.13 MHz, $CDCl_3$) and $^{13}C\{^{1}H\}$ (100.61 MHz, $CDCl_3$) spectra of **2e**

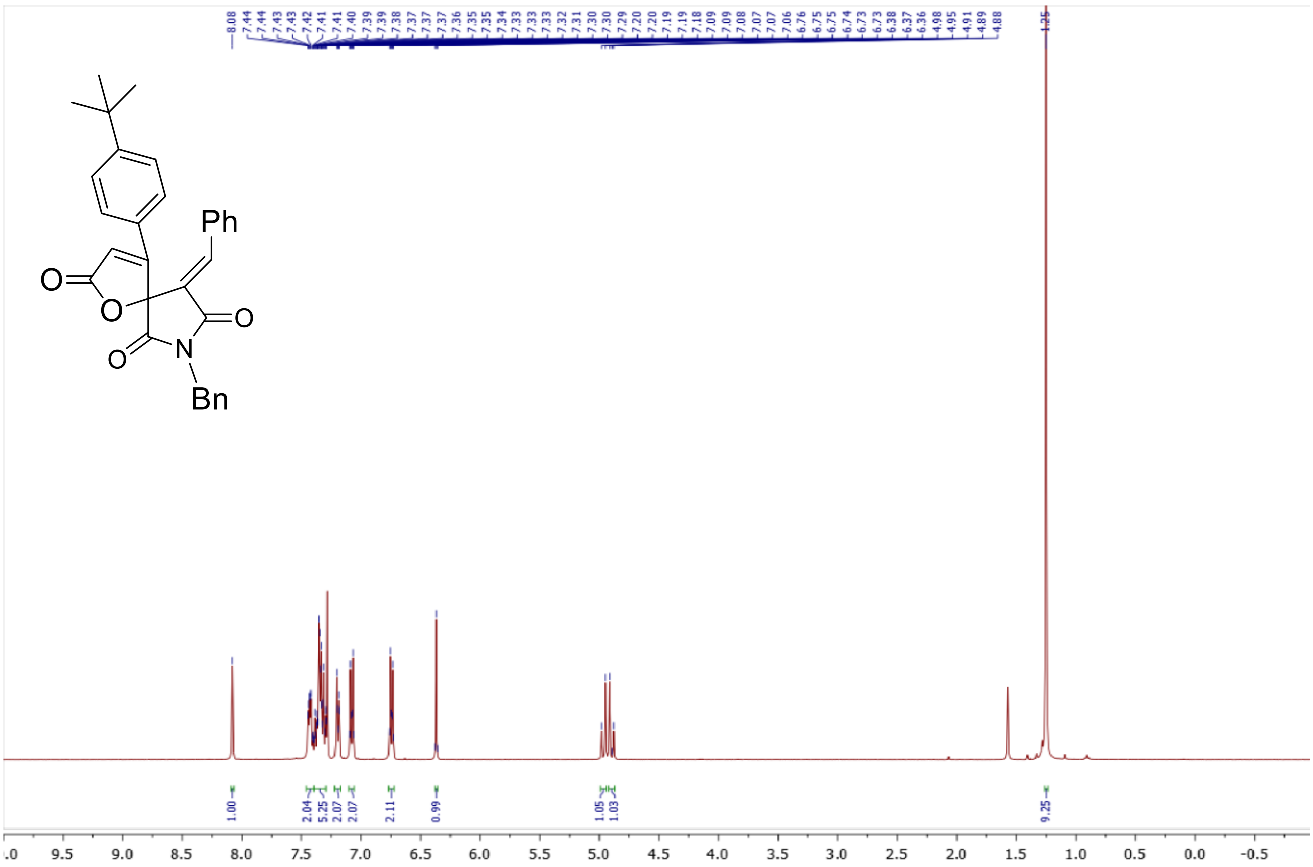

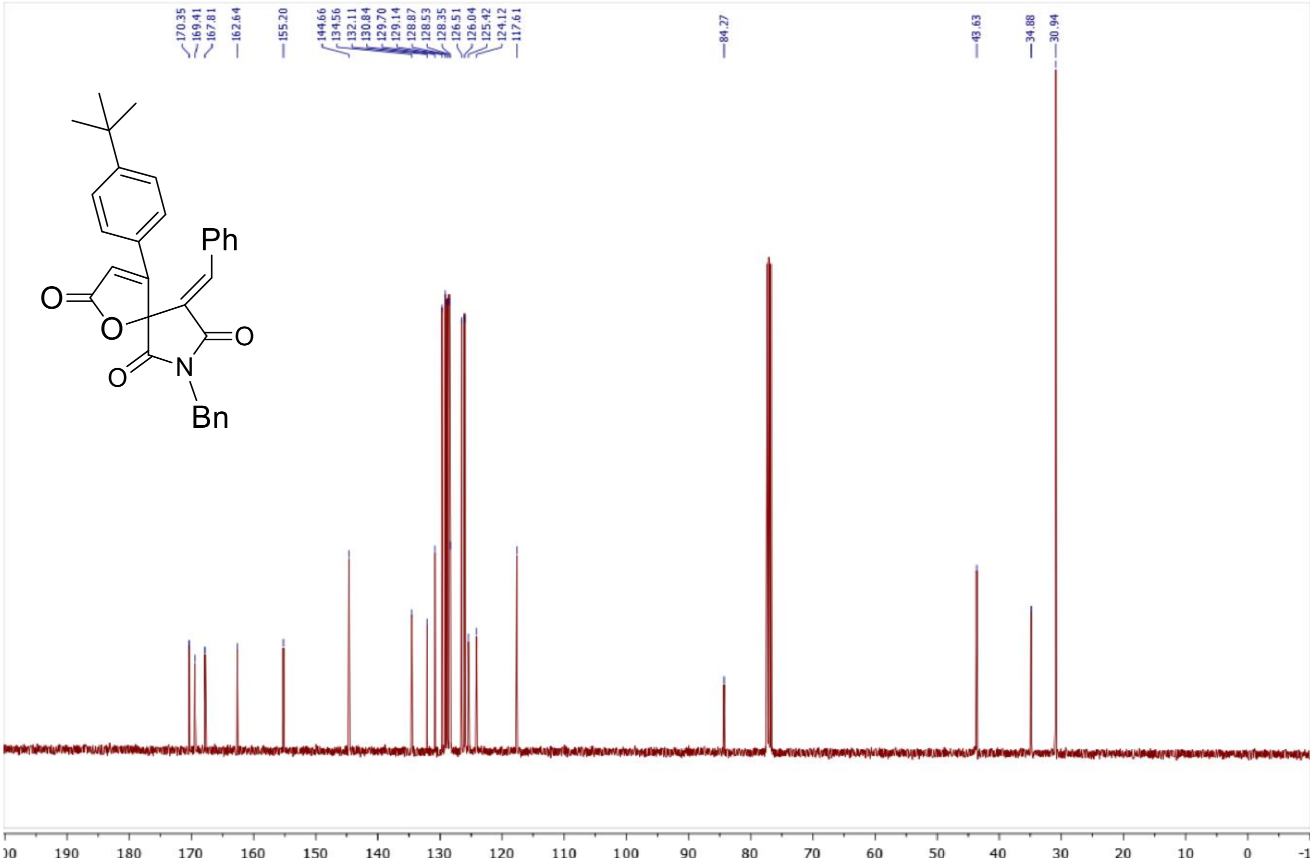

Copies of ^{1}H (400.13 MHz, $CDCl_3$) and $^{13}C\{^{1}H\}$ (100.61 MHz, $CDCl_3$) spectra of **2f**

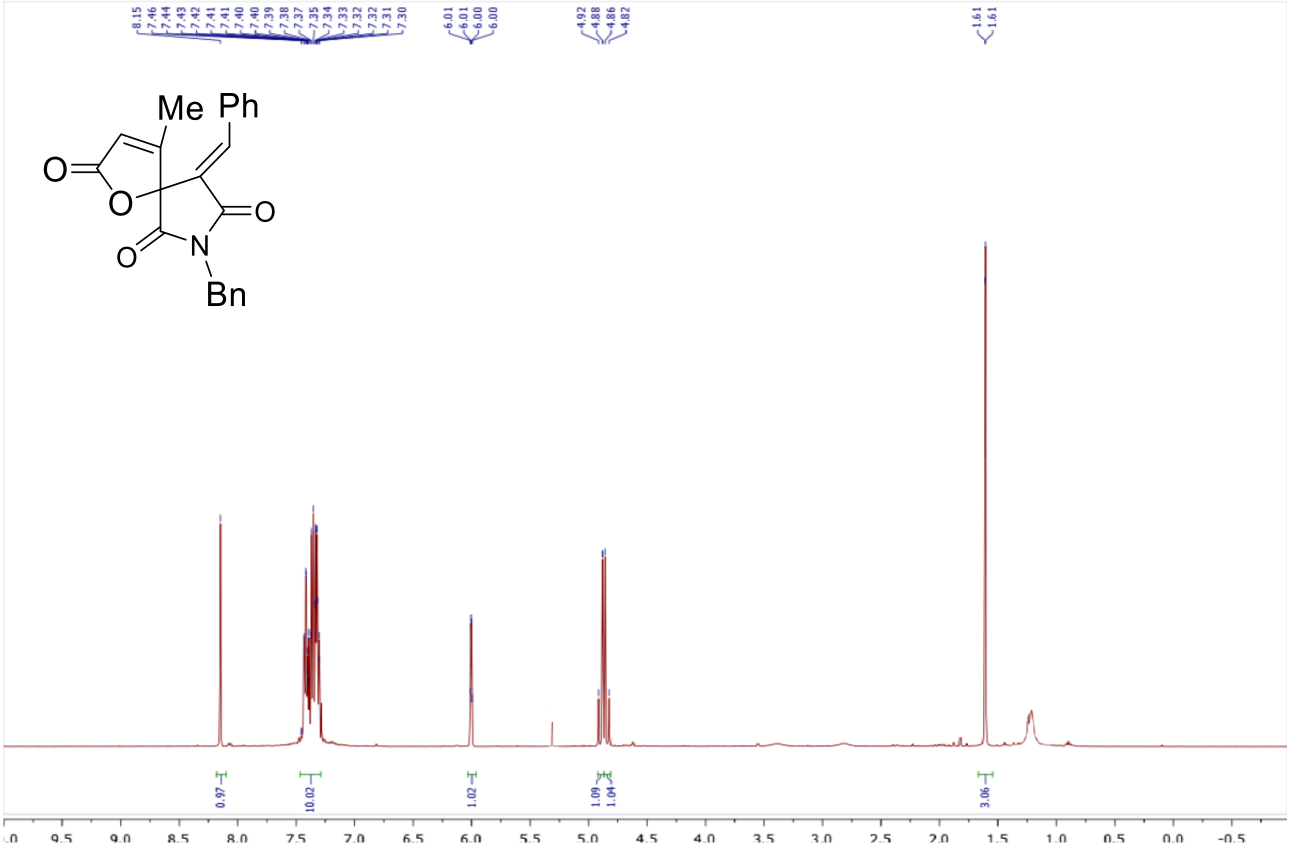

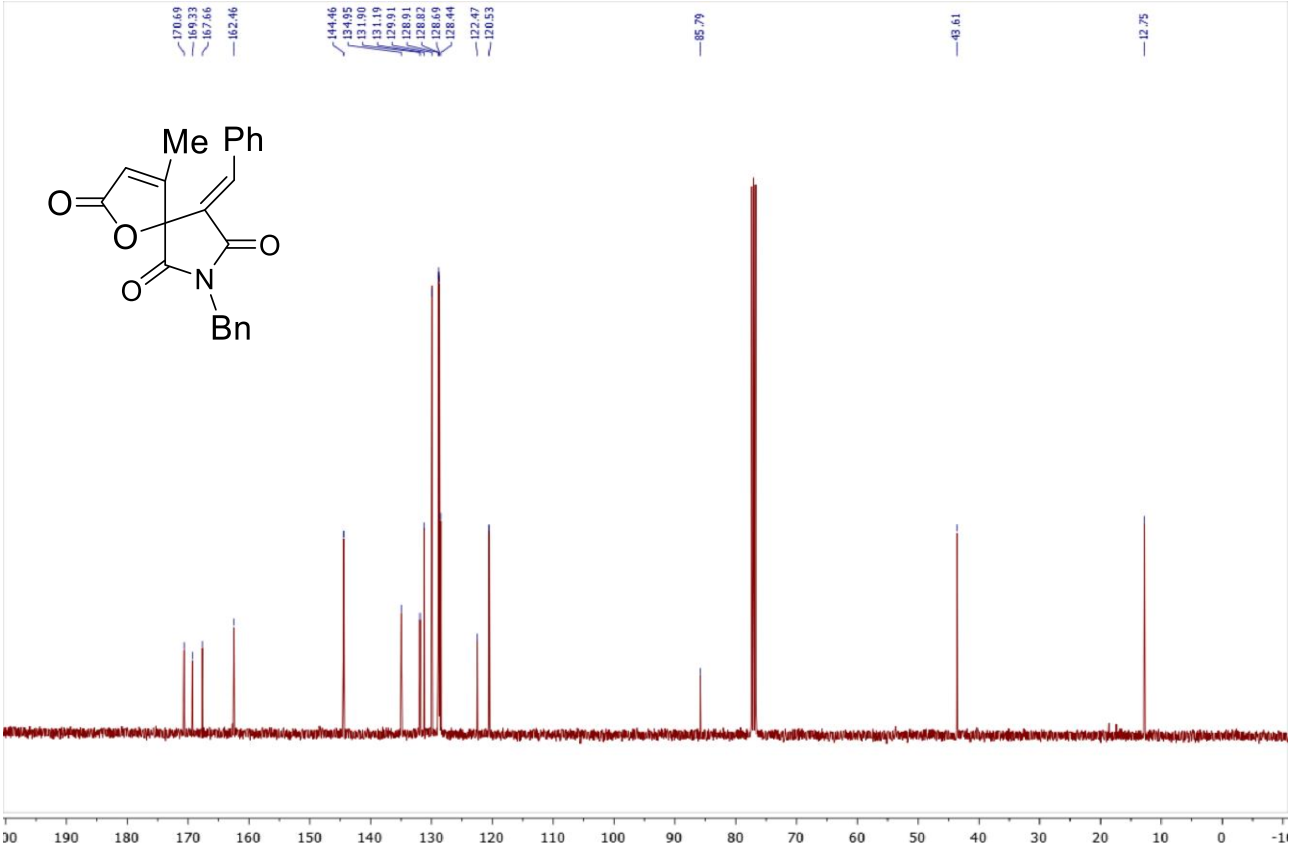

Copies of ^{1}H (400.13 MHz, $CDCl_3$) and $^{13}C\{^{1}H\}$ (100.61 MHz, $CDCl_3$) spectra of **2g**

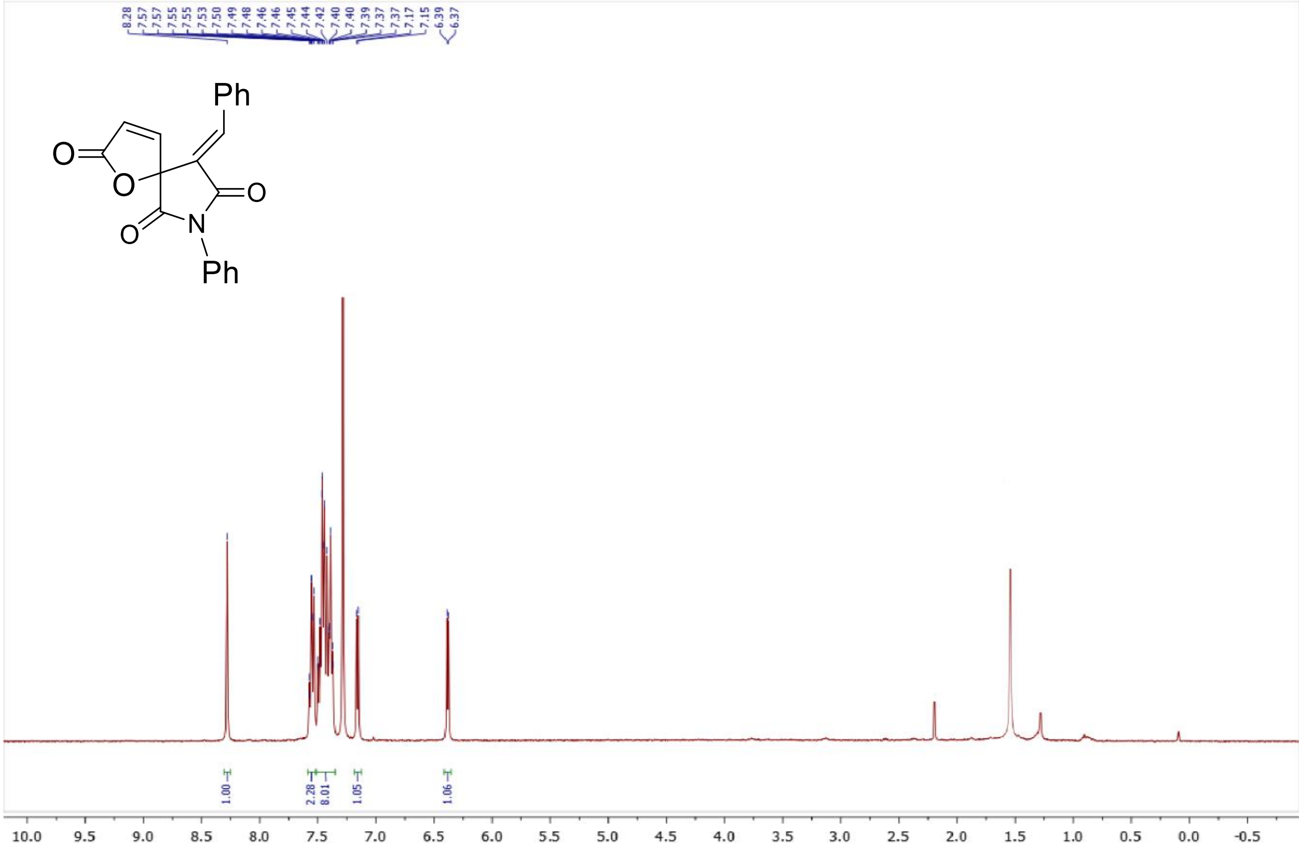

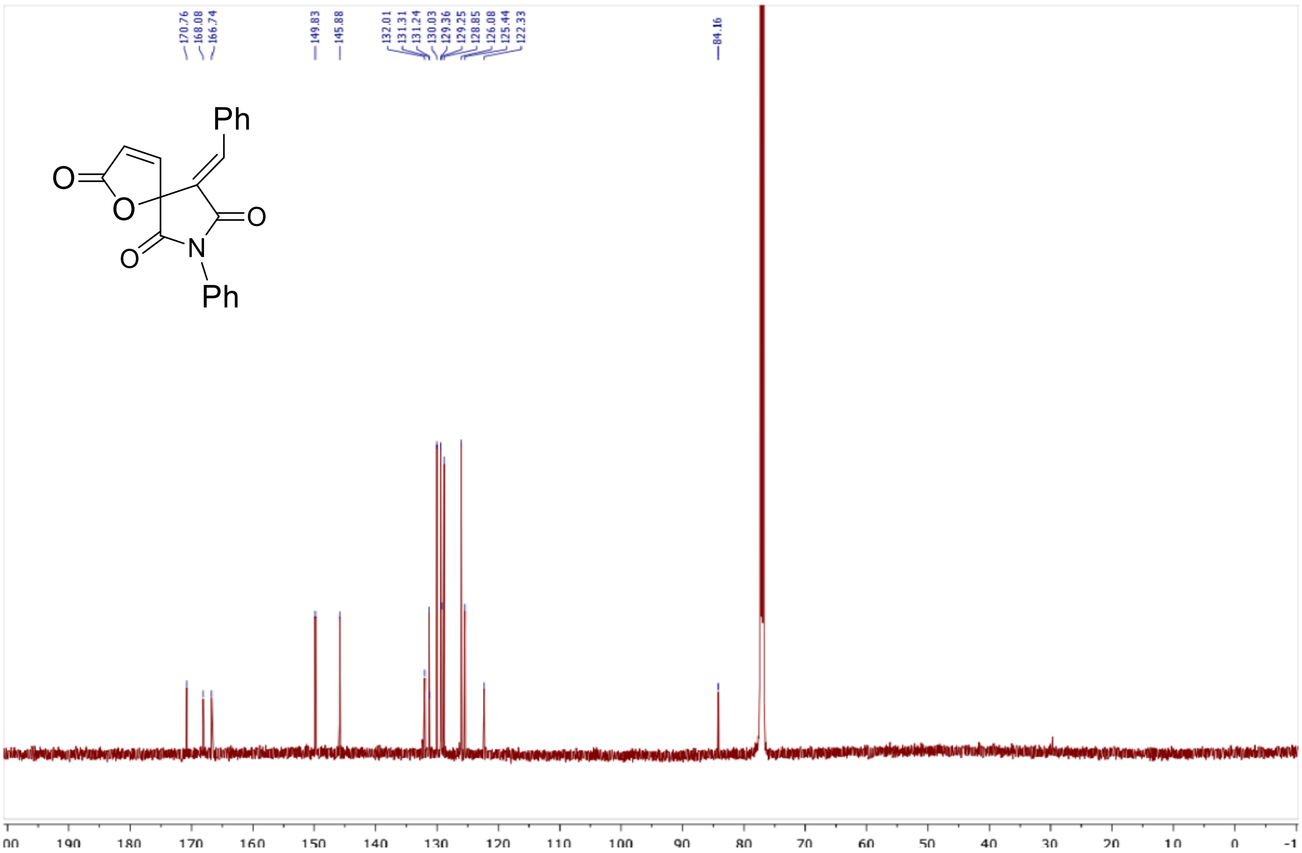

Copies of ^{1}H (400.13 MHz, $CDCl_3$) and $^{13}C\{^{1}H\}$ (100.61 MHz, $CDCl_3$) spectra of **2h**

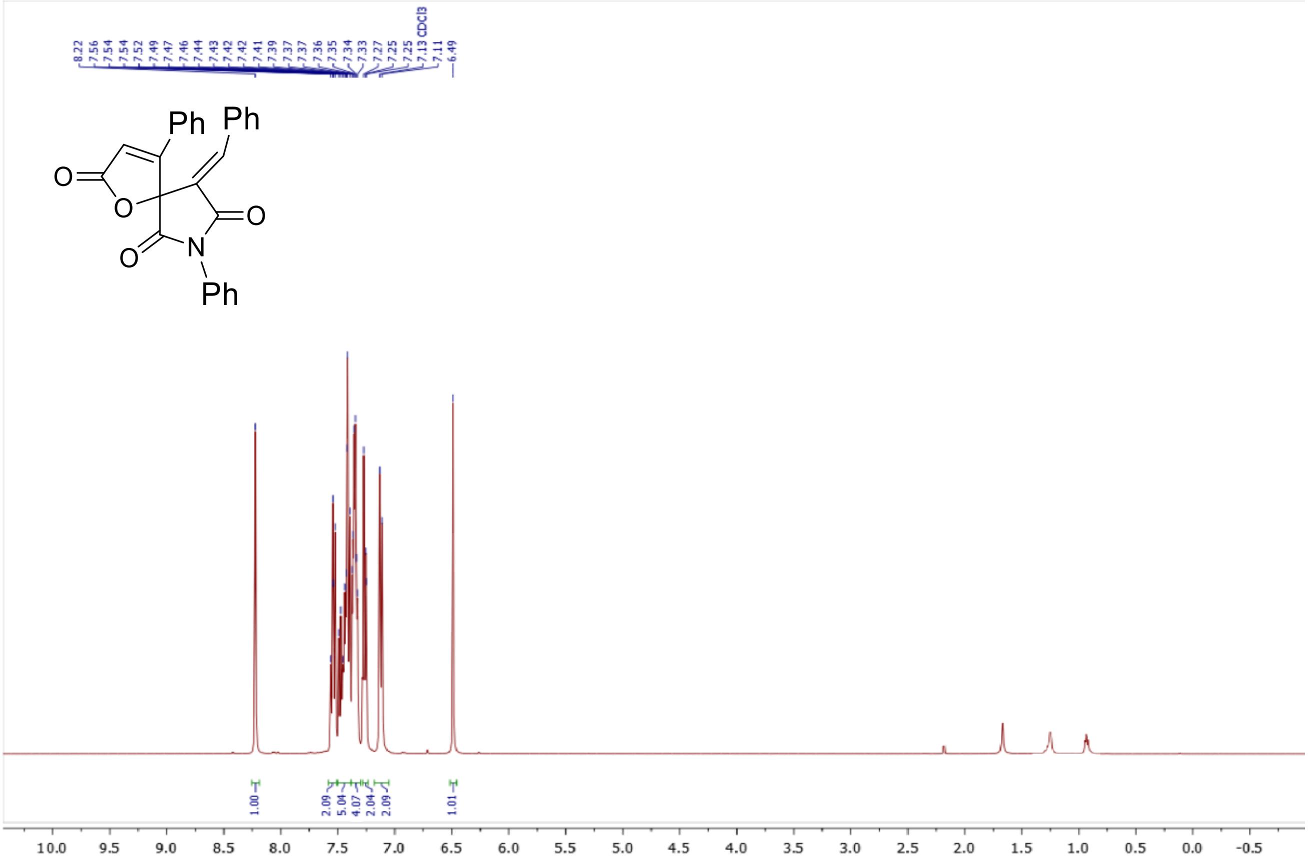

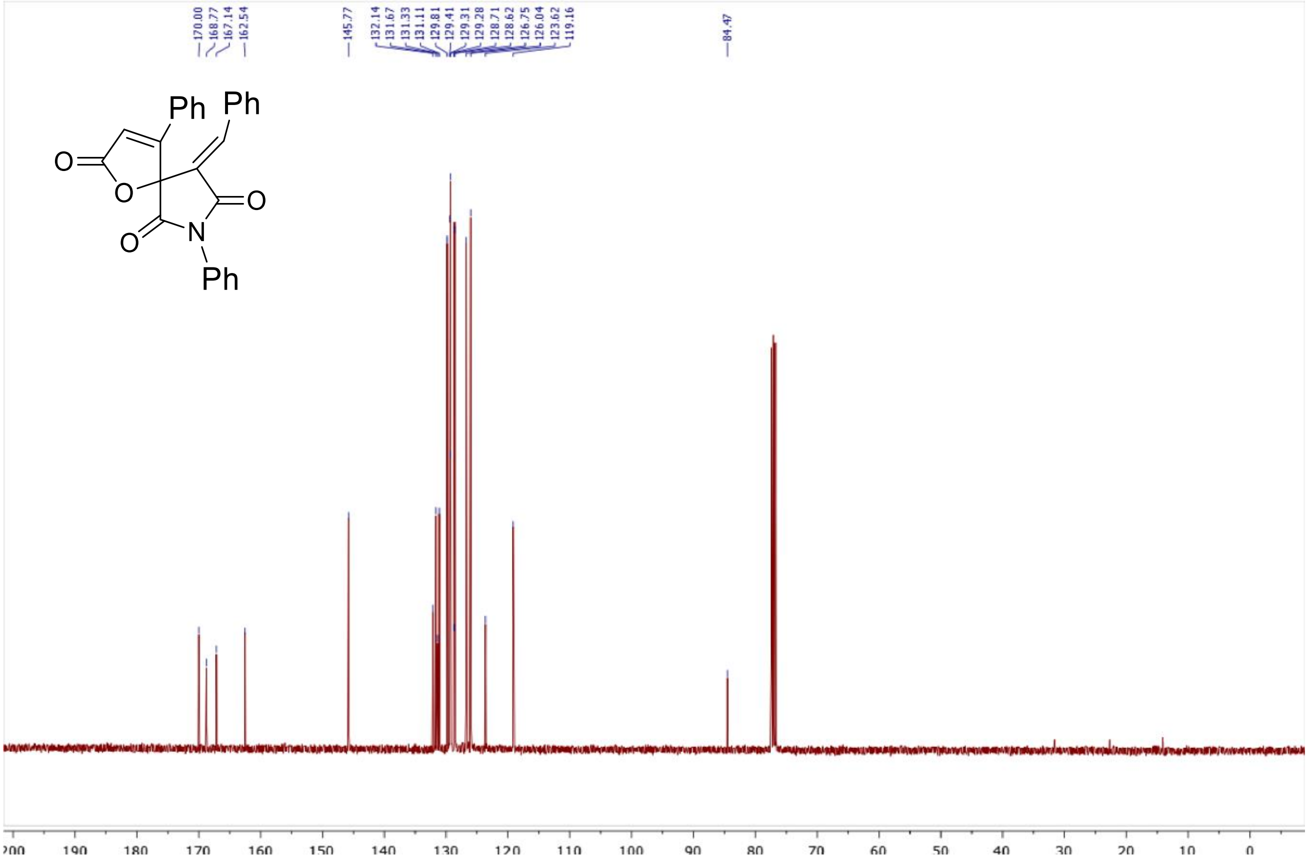

Copies of ^{1}H (400.13 MHz, $CDCl_3$) and $^{13}C\{^{1}H\}$ (100.61 MHz, $CDCl_3$) spectra of **2i**

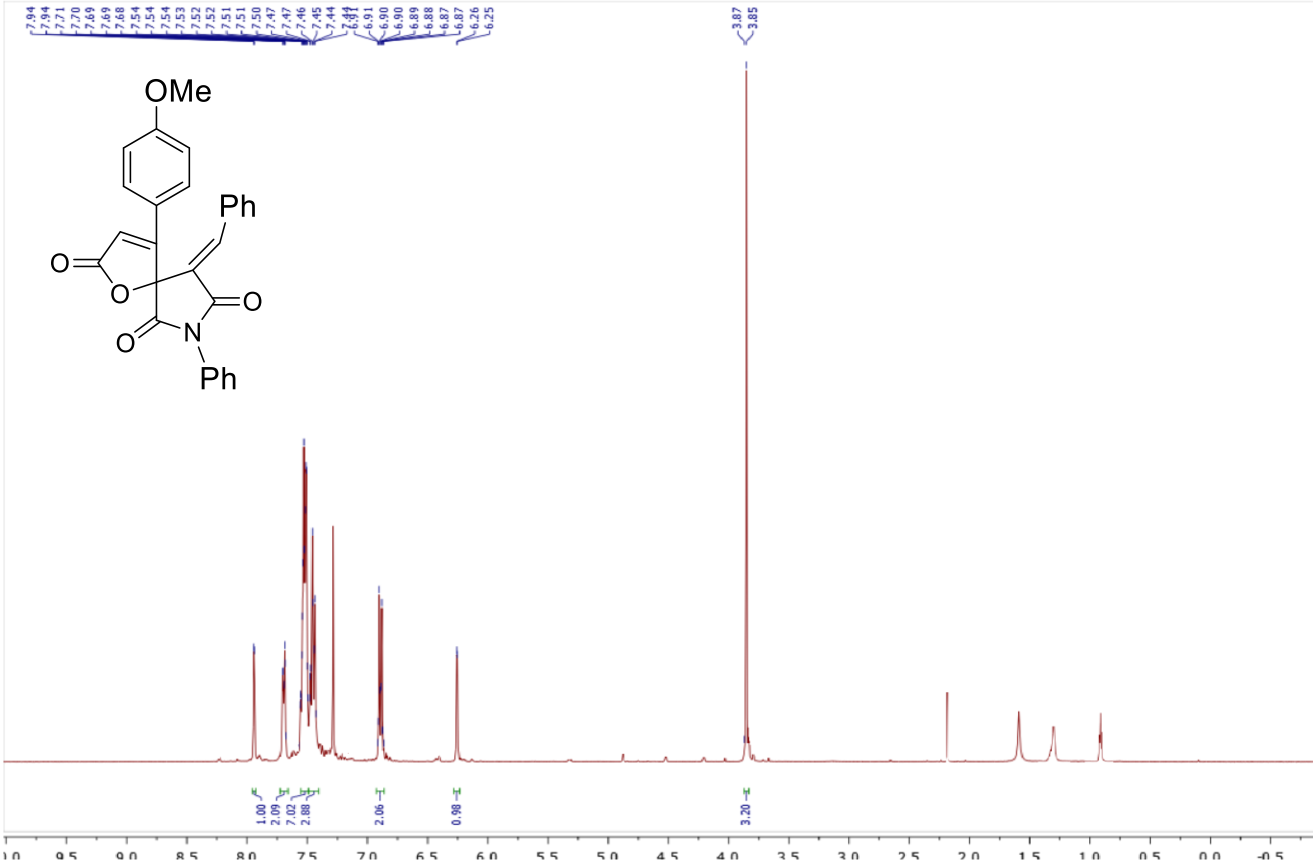

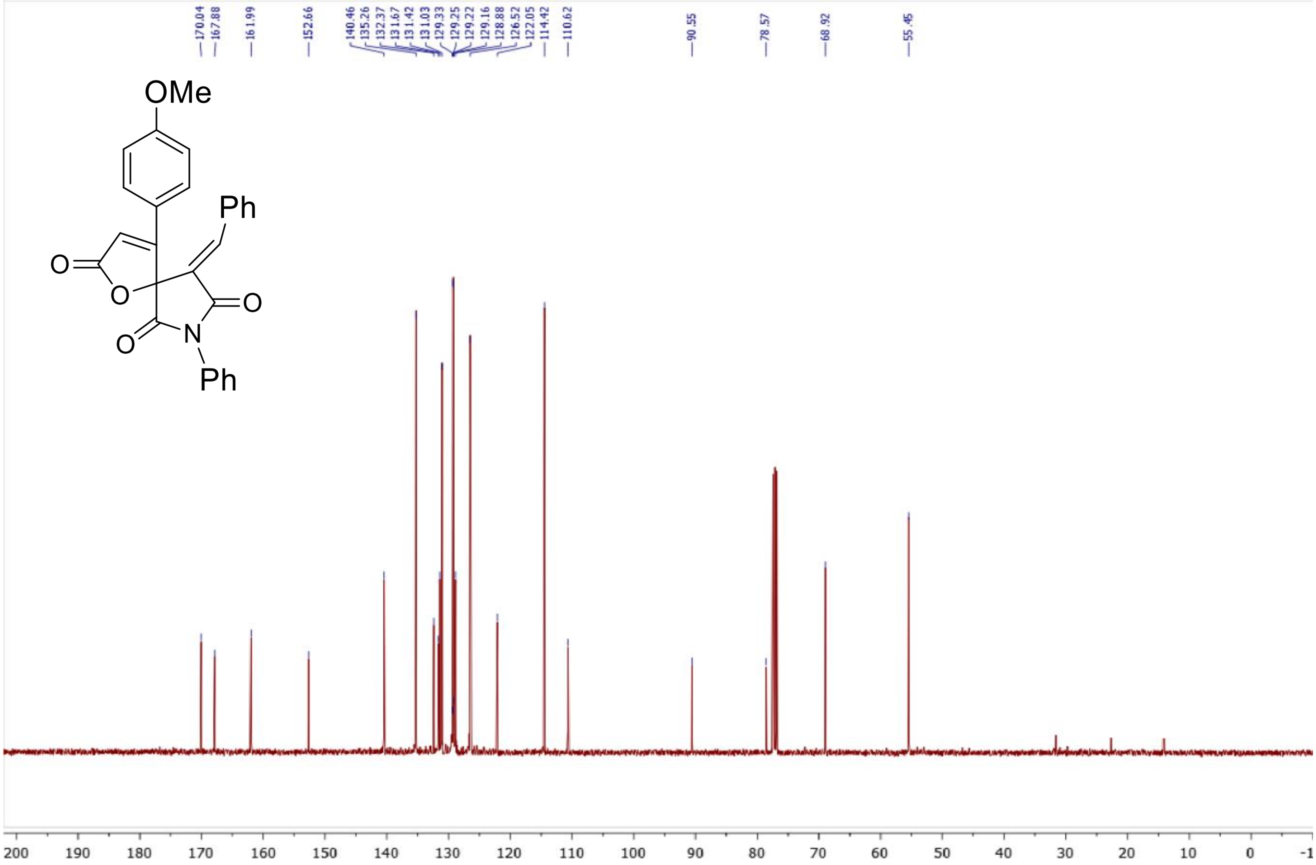

Copies of ^{1}H (400.13 MHz, $CDCl_3$) and ^{13}C{^{1}H} (100.61 MHz, $CDCl_3$) spectra of **2j**

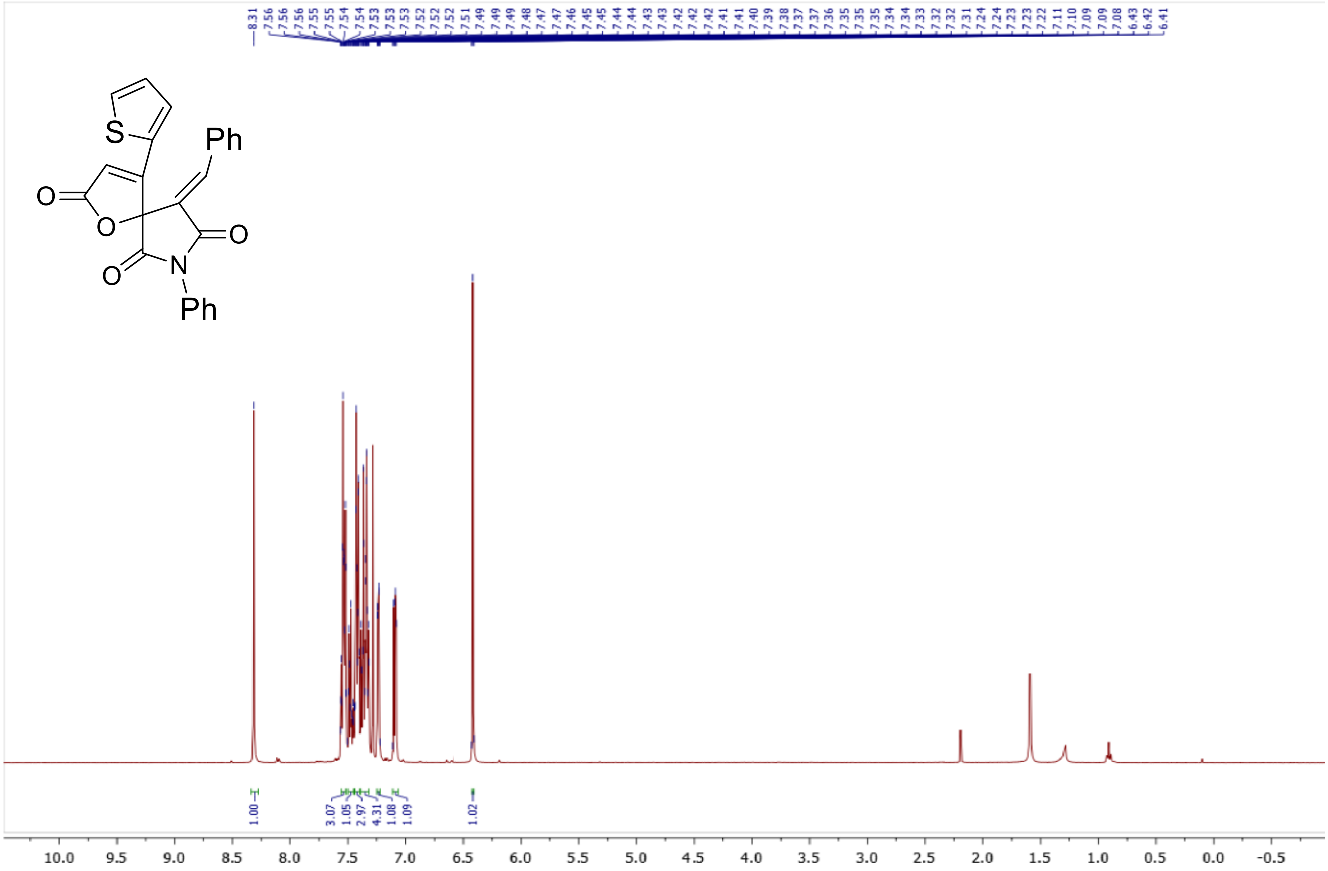

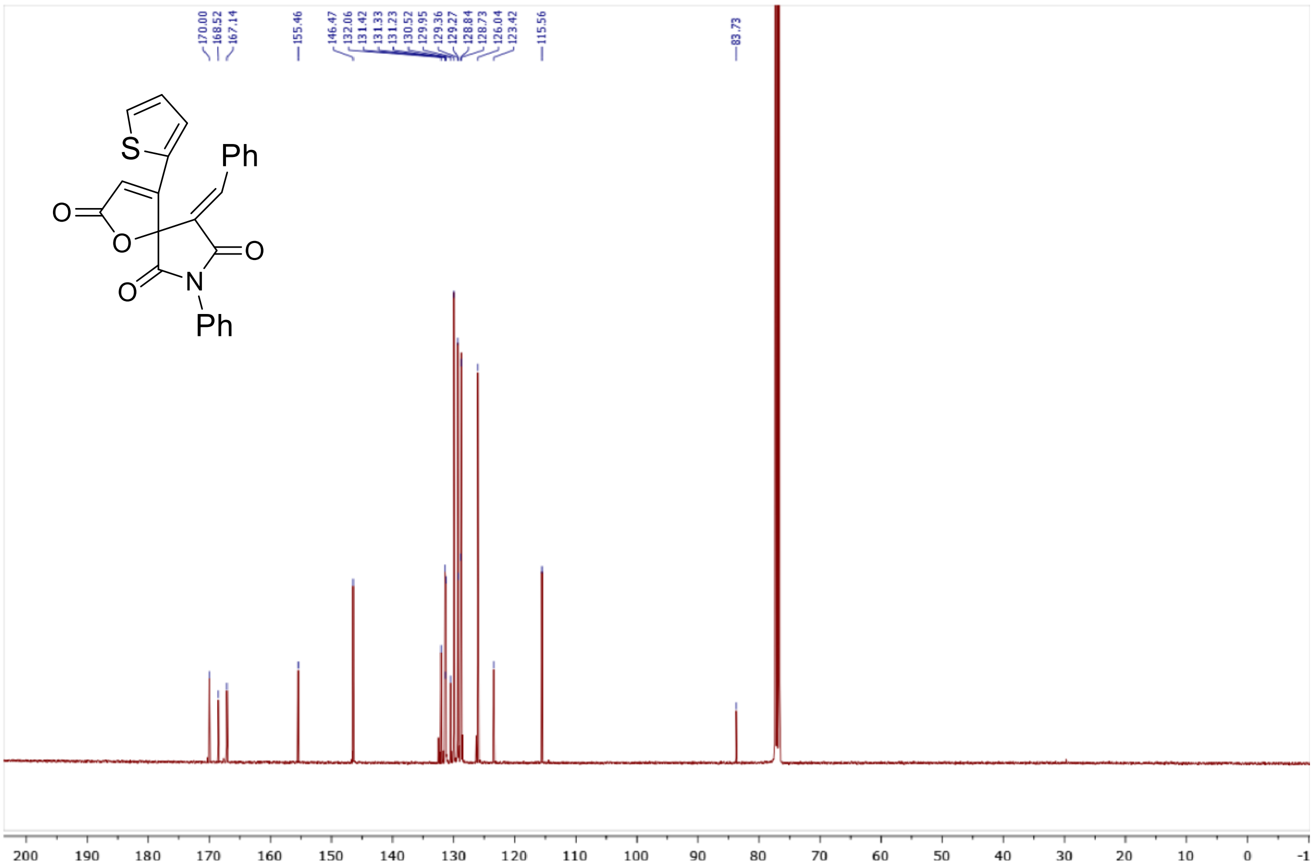

Copies of ^{1}H (400.13 MHz, CDCl$_3$) and ^{13}C{^{1}H} (100.61 MHz, CDCl$_3$) spectra of **2k**

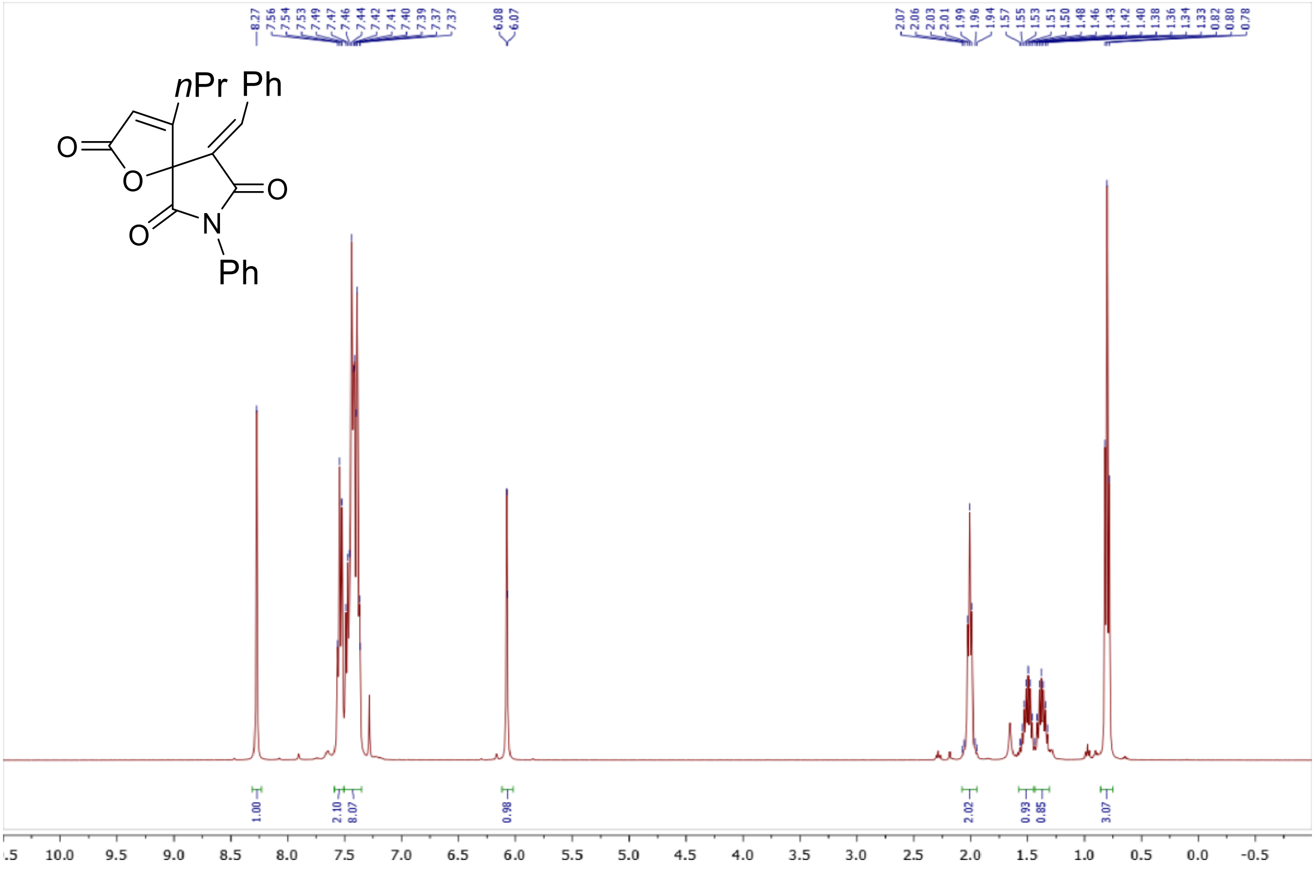

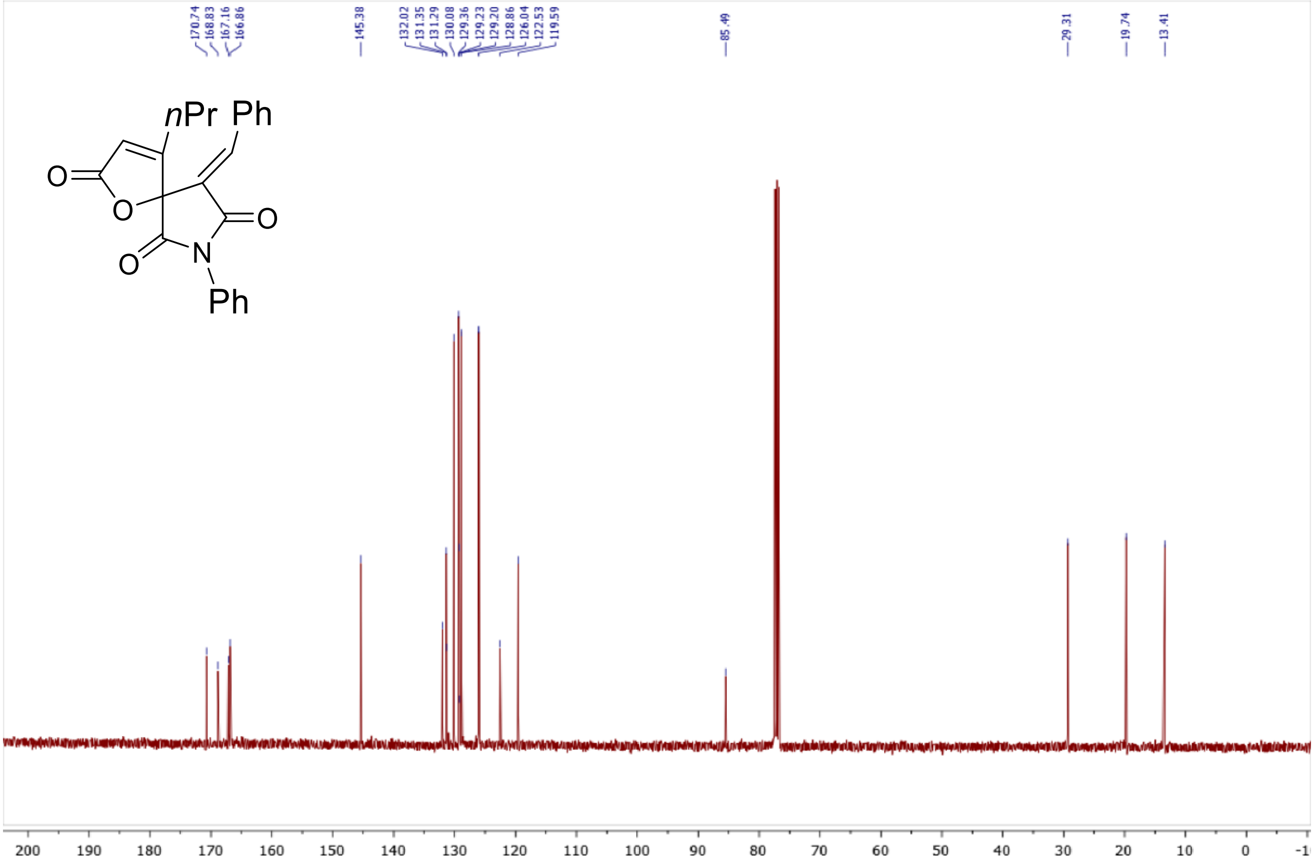

Copies of ^{1}H (400.13 MHz, $CDCl_3$) and $^{13}C\{^{1}H\}$ (100.61 MHz, $CDCl_3$) spectra of **3a**

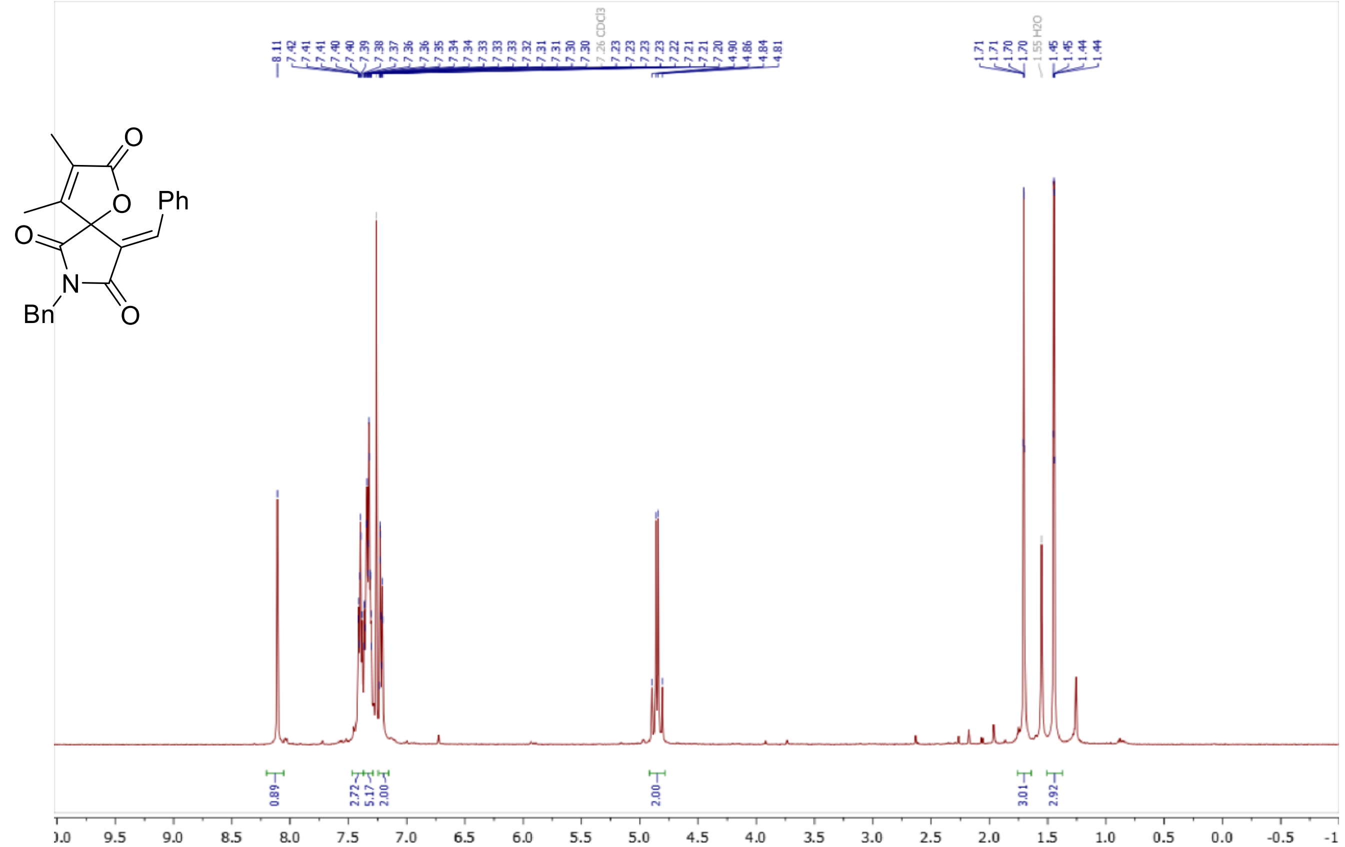

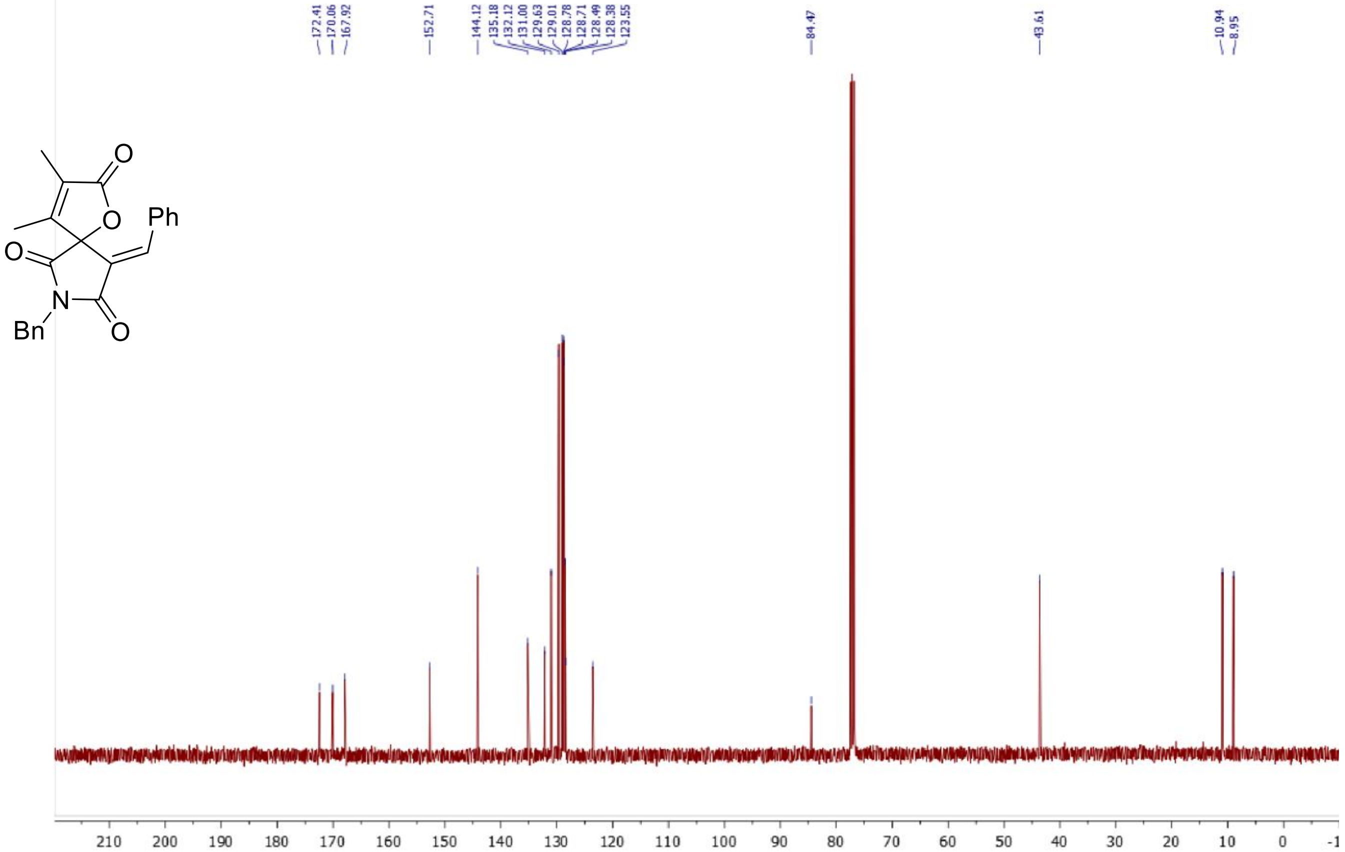

Copies of ^{1}H (400.13 MHz, $CDCl_3$) and $^{13}C\{^{1}H\}$ (100.61 MHz, $CDCl_3$) spectra of **3b**

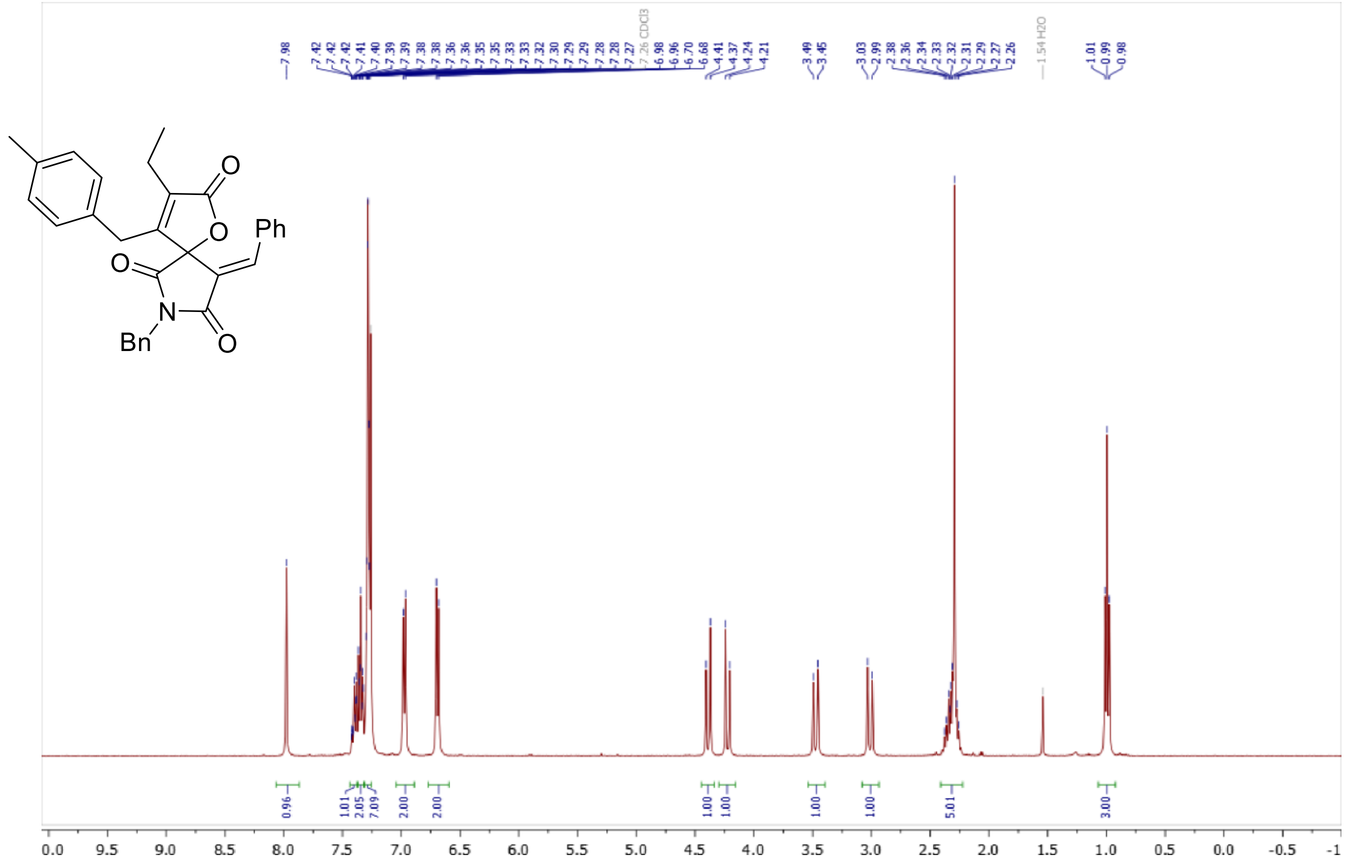

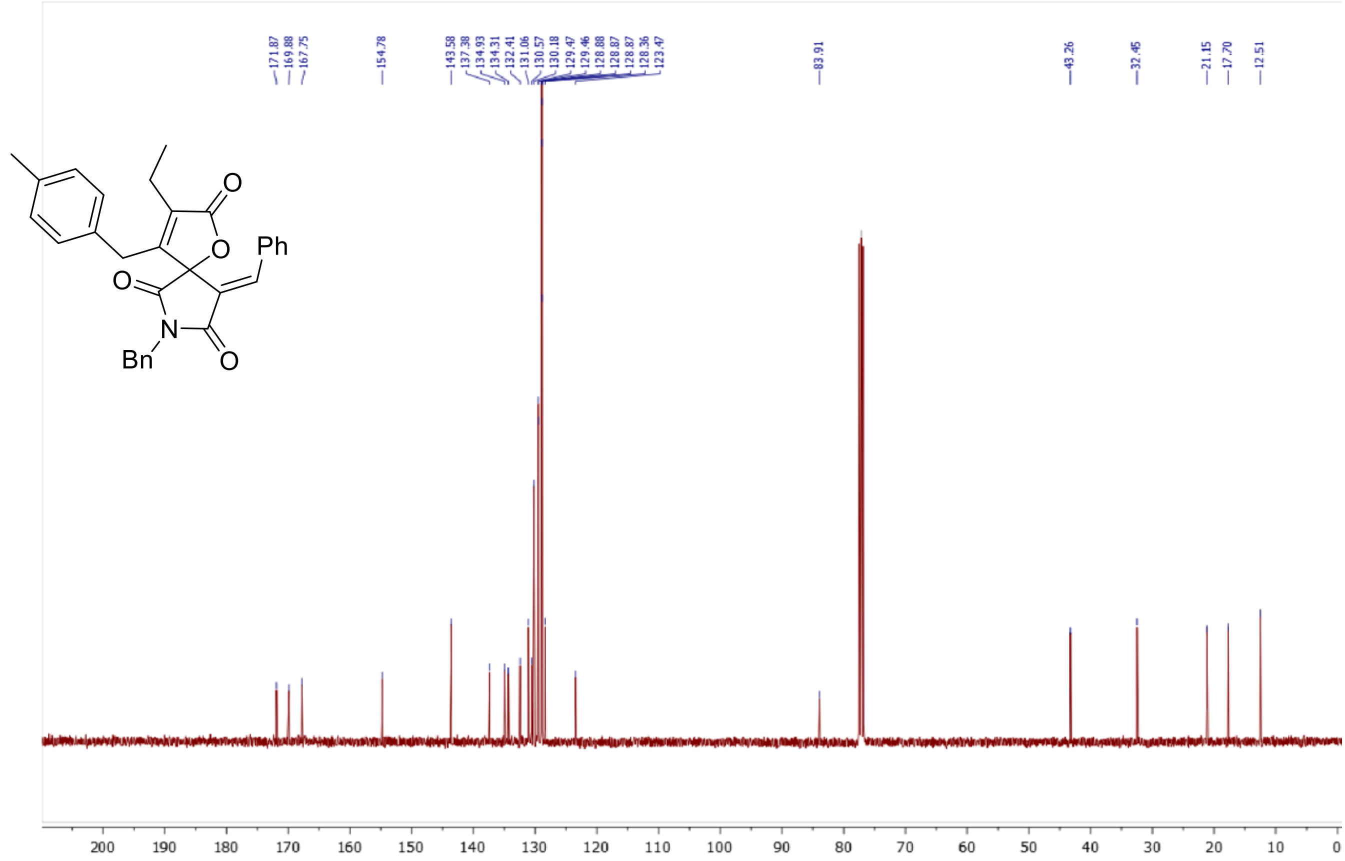

Copies of ^{1}H (400.13 MHz, $CDCl_3$) and $^{13}C\{^{1}H\}$ (100.61 MHz, $CDCl_3$) spectra of **3c**

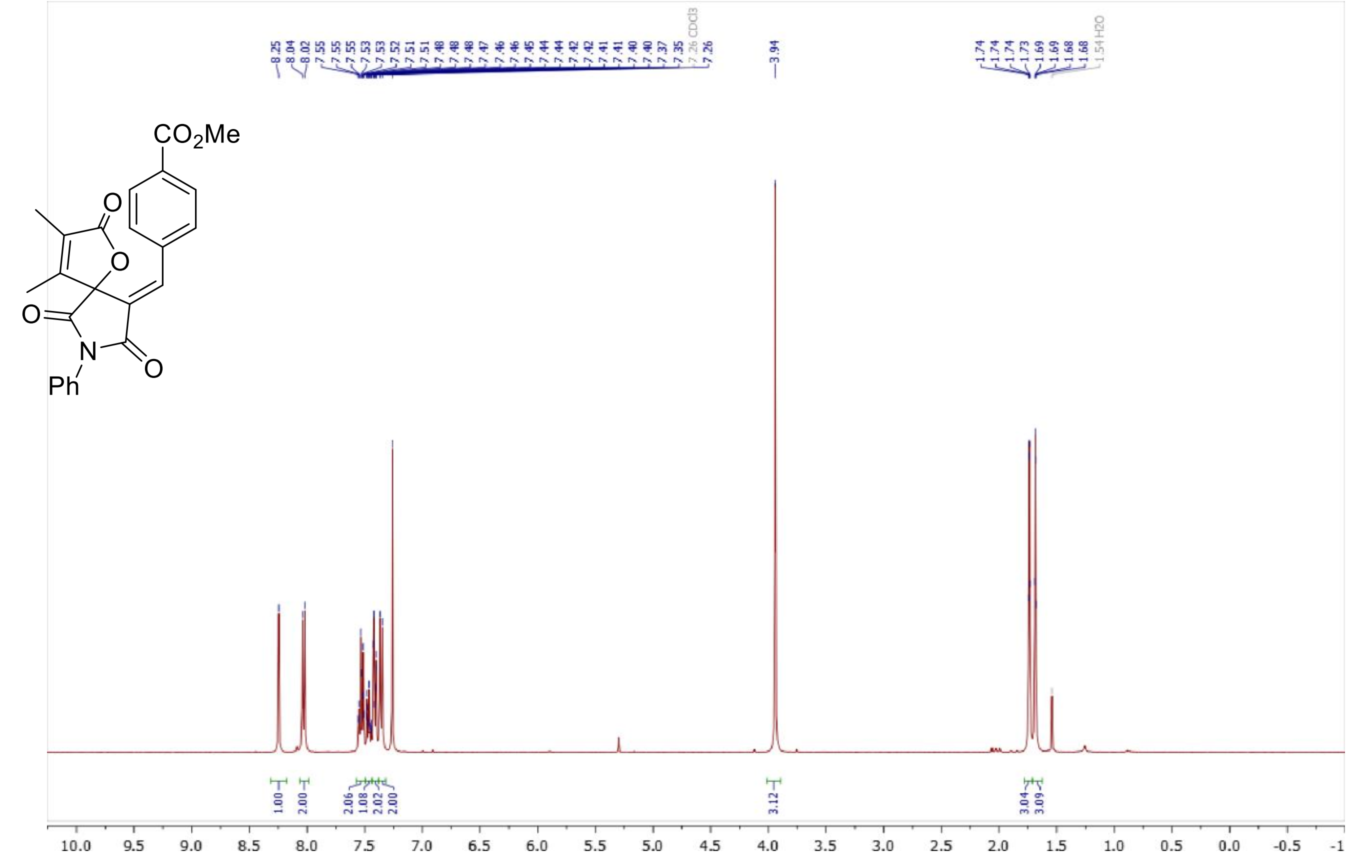

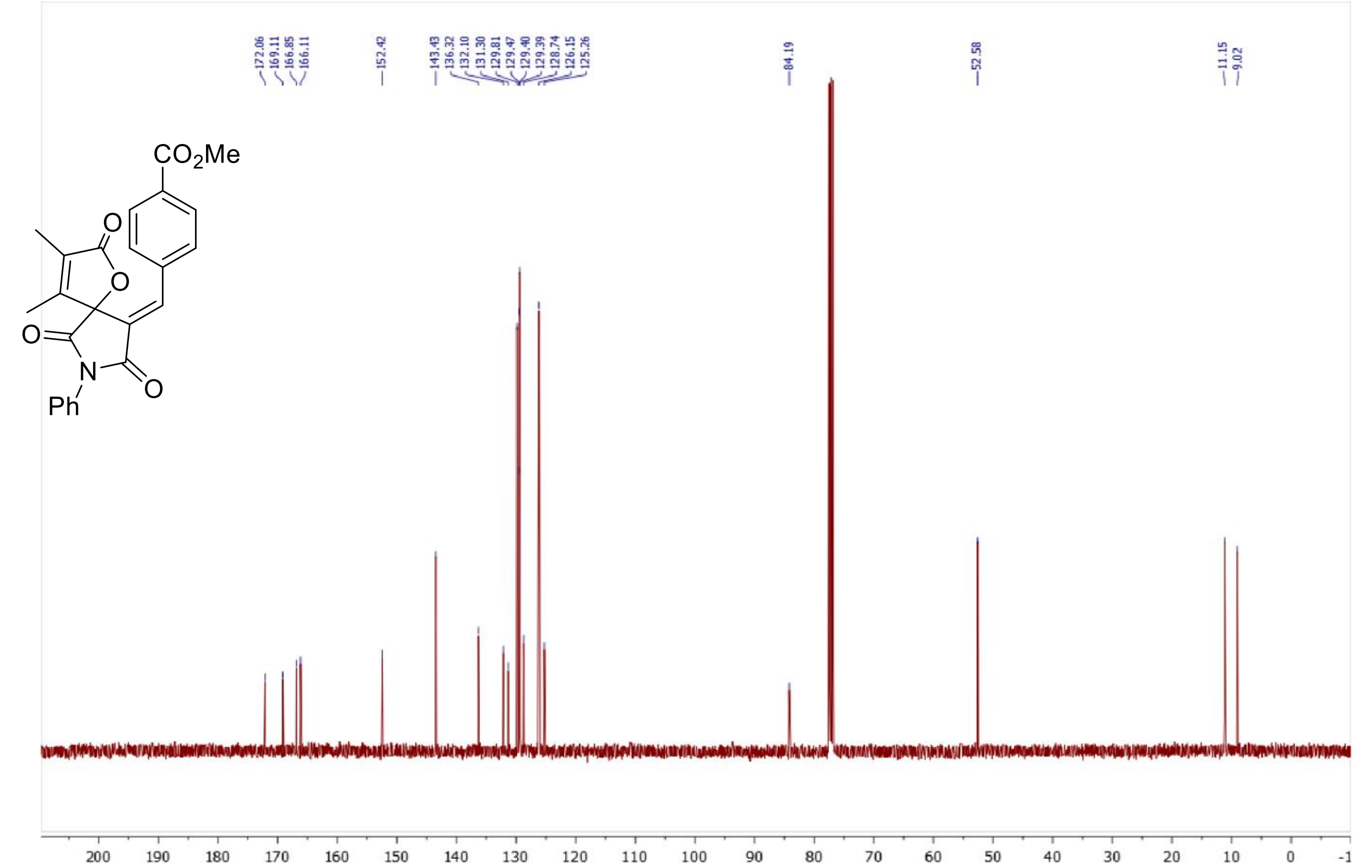

Copies of ^{1}H (400.13 MHz, $CDCl_3$) and $^{13}C\{^{1}H\}$ (100.61 MHz, $CDCl_3$) spectra of **3d**

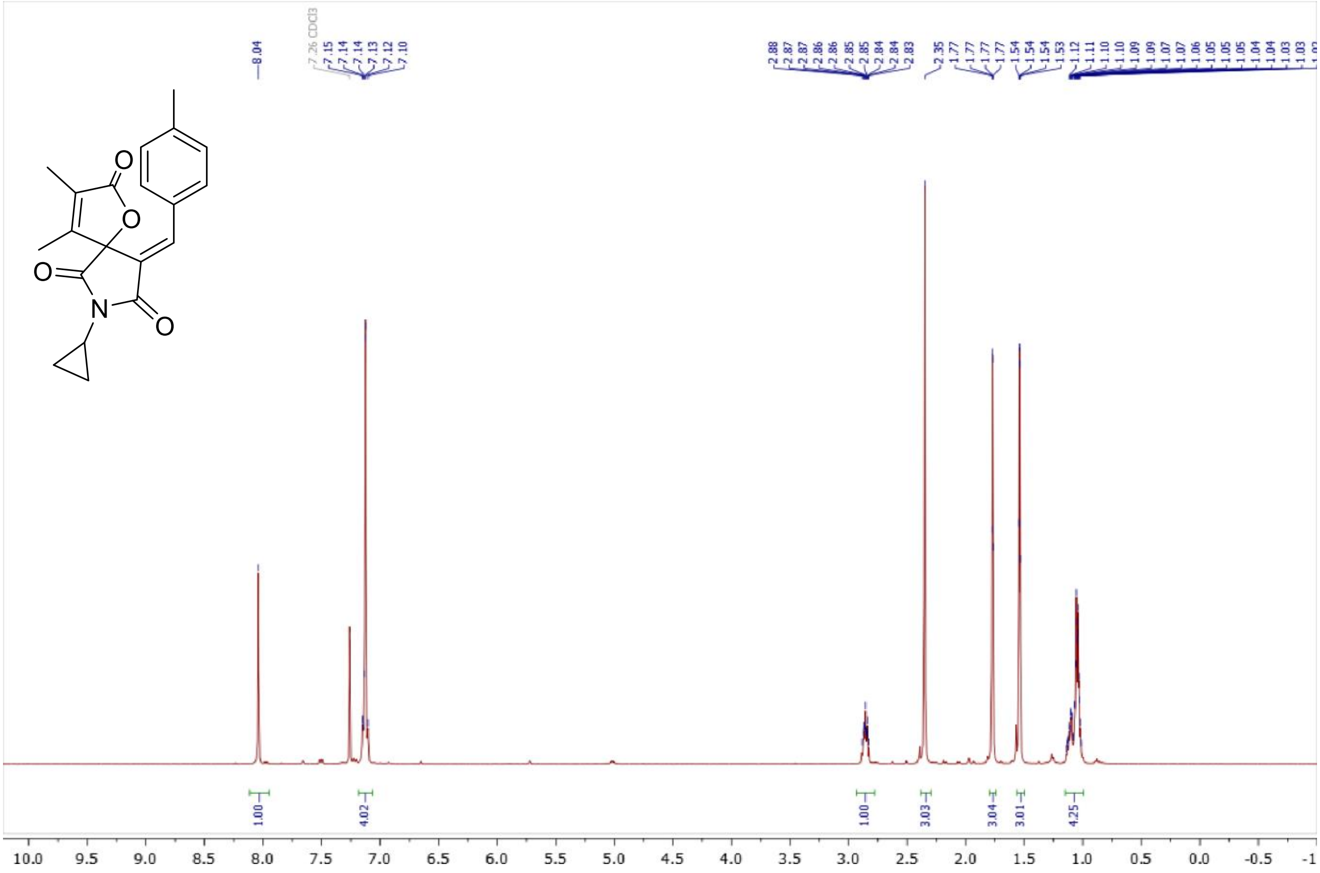

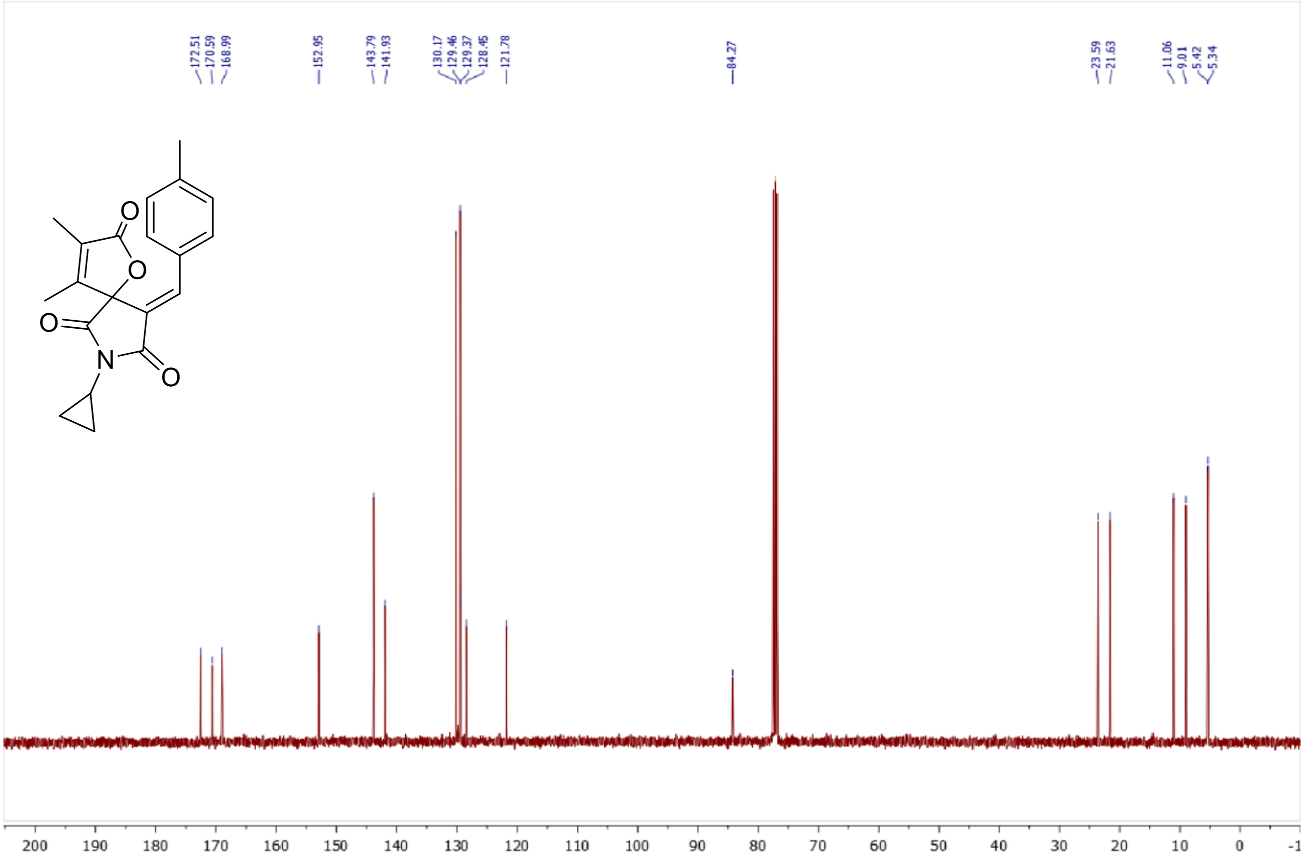

Copies of ^{1}H (400.13 MHz, $CDCl_3$) and ^{13}C{^{1}H} (100.61 MHz, $CDCl_3$) spectra of **4a**

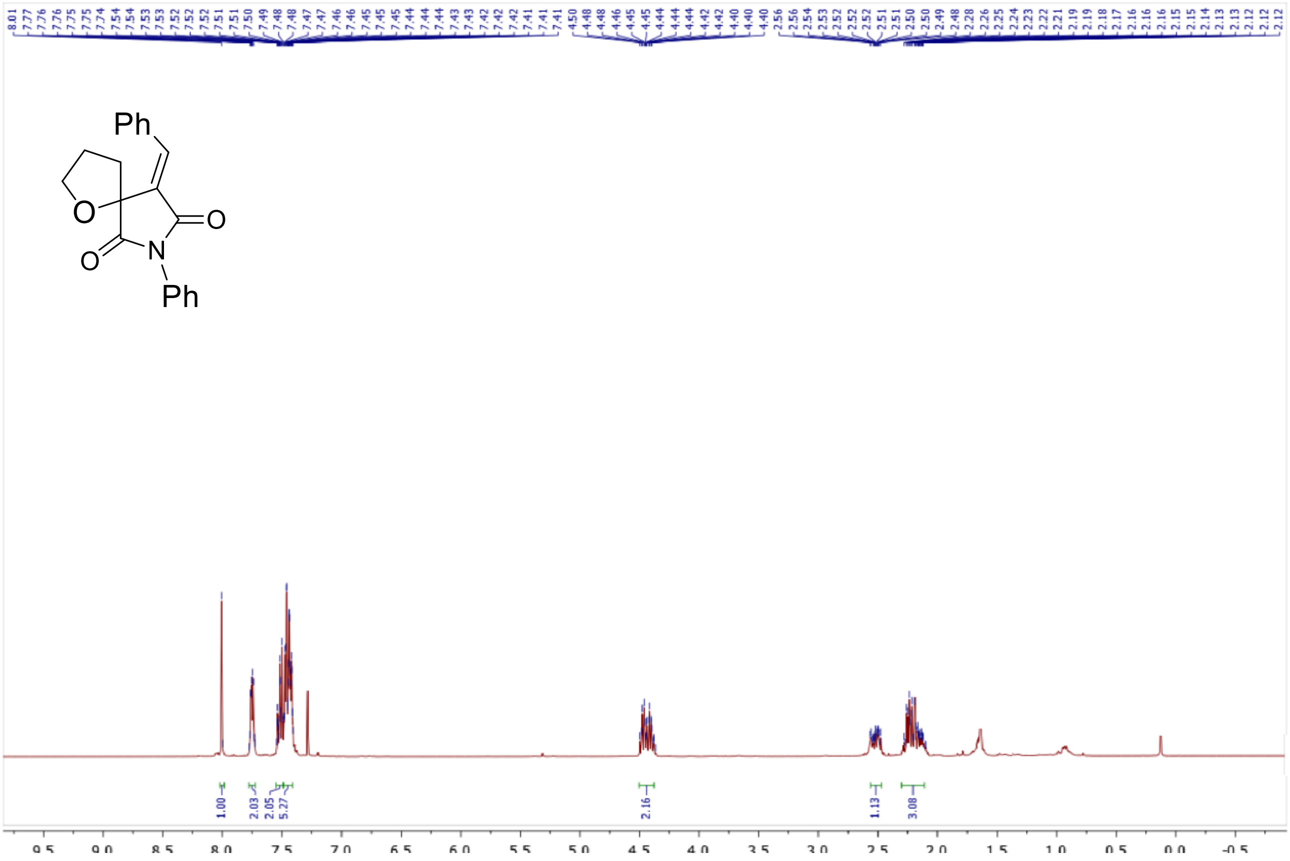

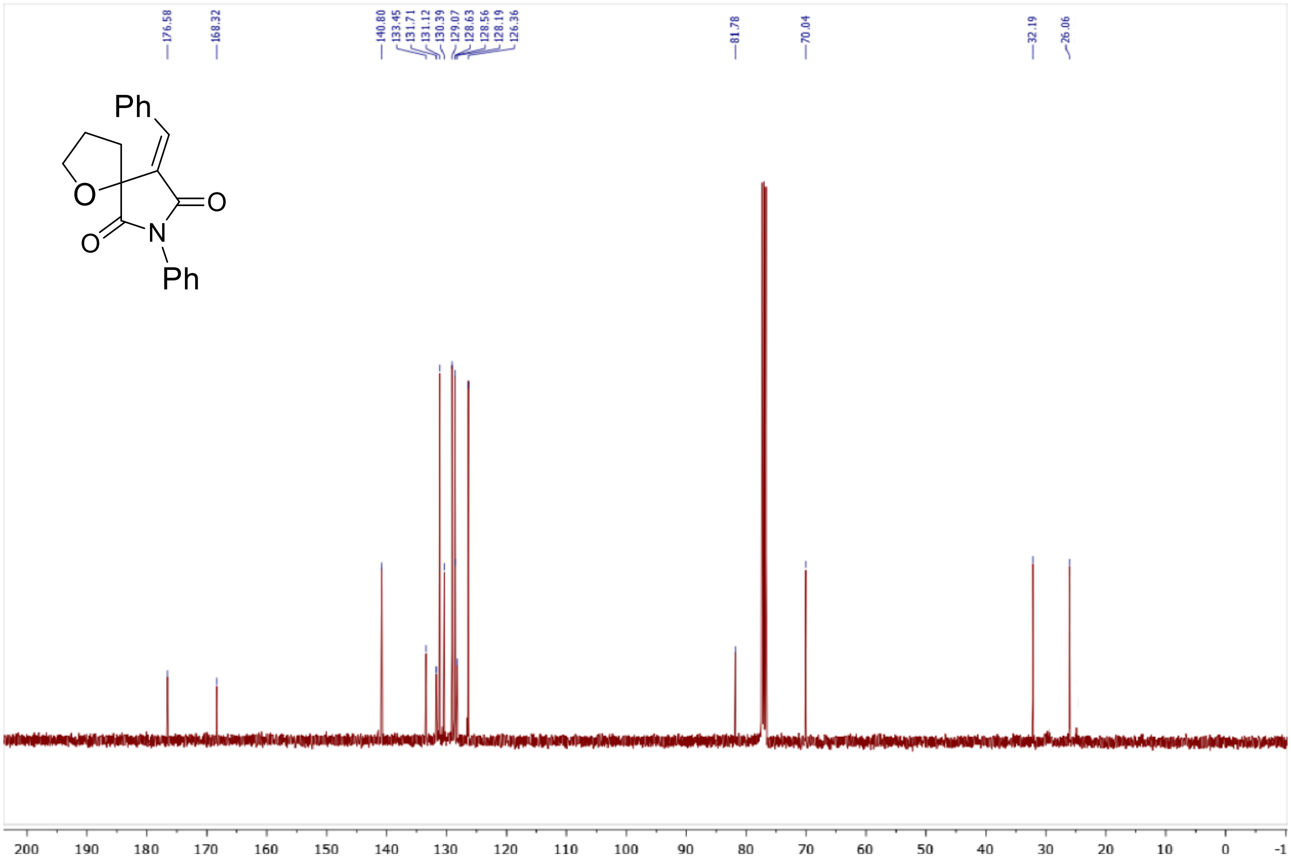

Copies of ^{1}H (400.13 MHz, CDCl$_3$) and ^{13}C{^{1}H} (100.61 MHz, CDCl$_3$) spectra of **4b**

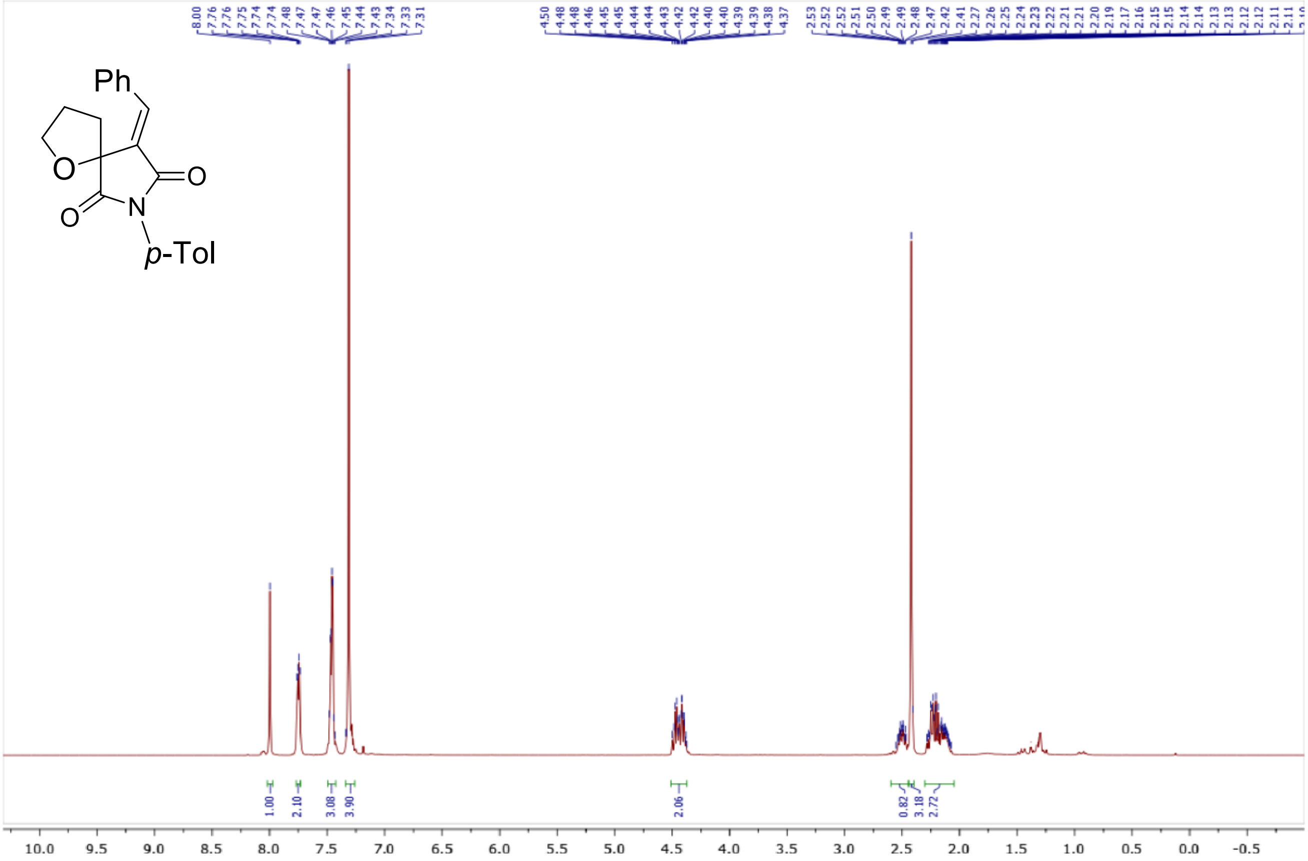

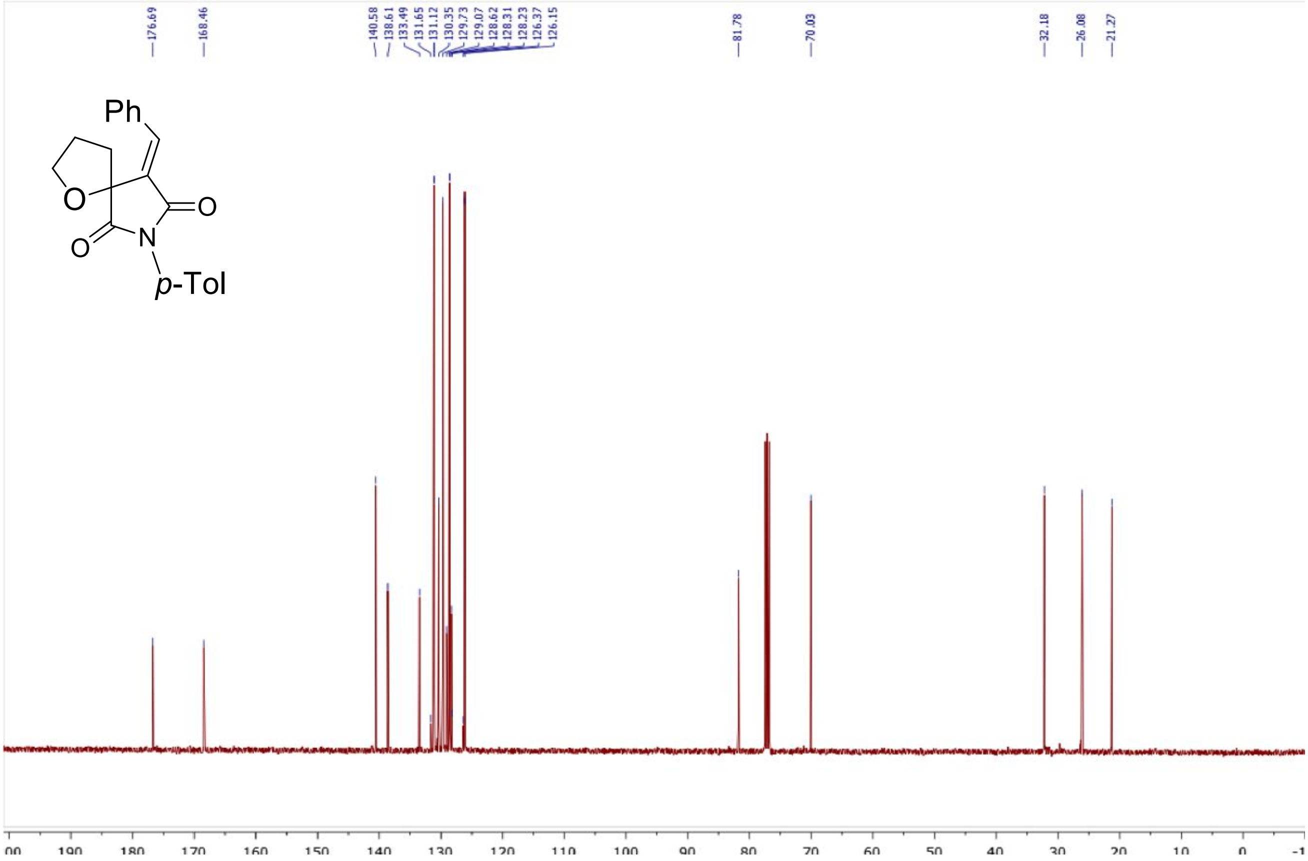

Copies of ^{1}H (400.13 MHz, $CDCl_3$) and $^{13}C\{^1H\}$ (100.61 MHz, $CDCl_3$) spectra of **4c**

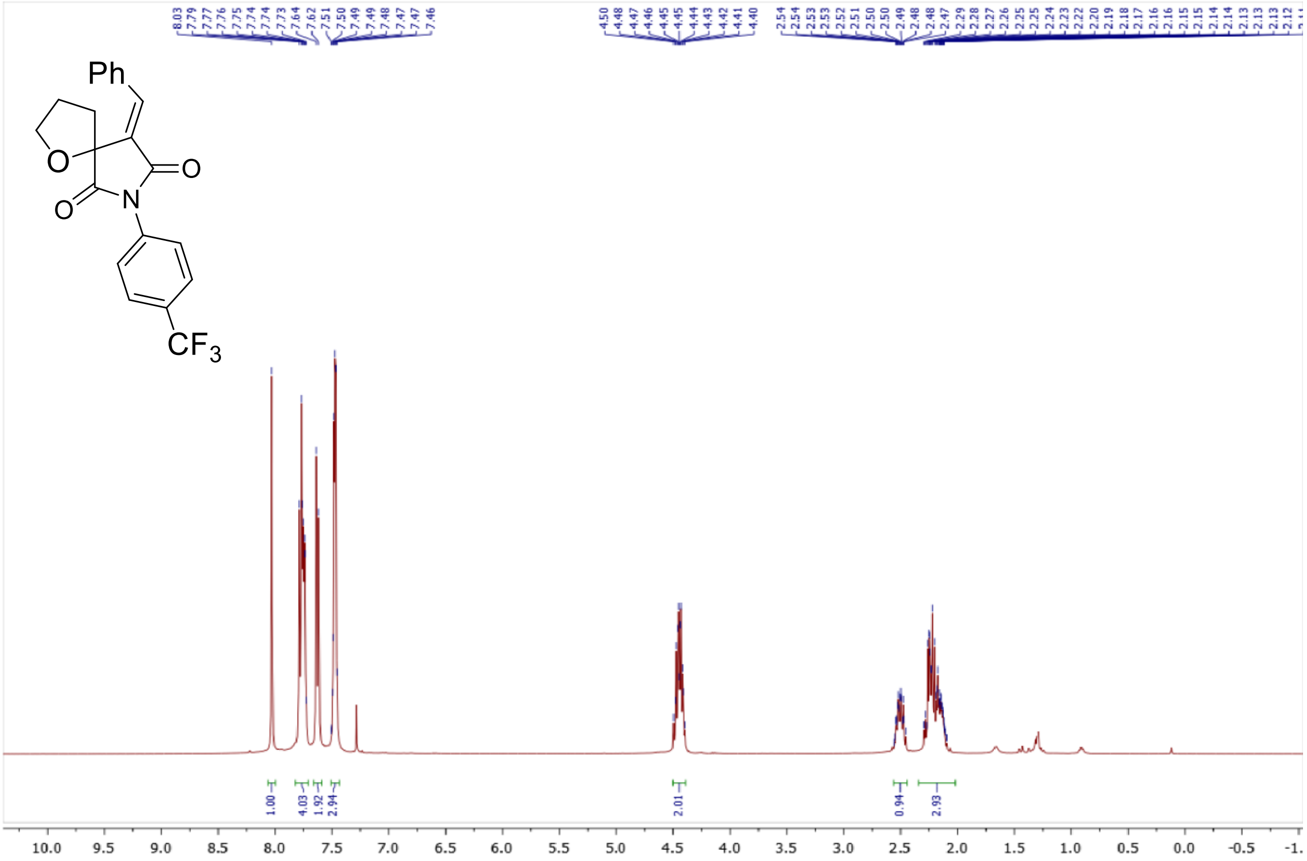

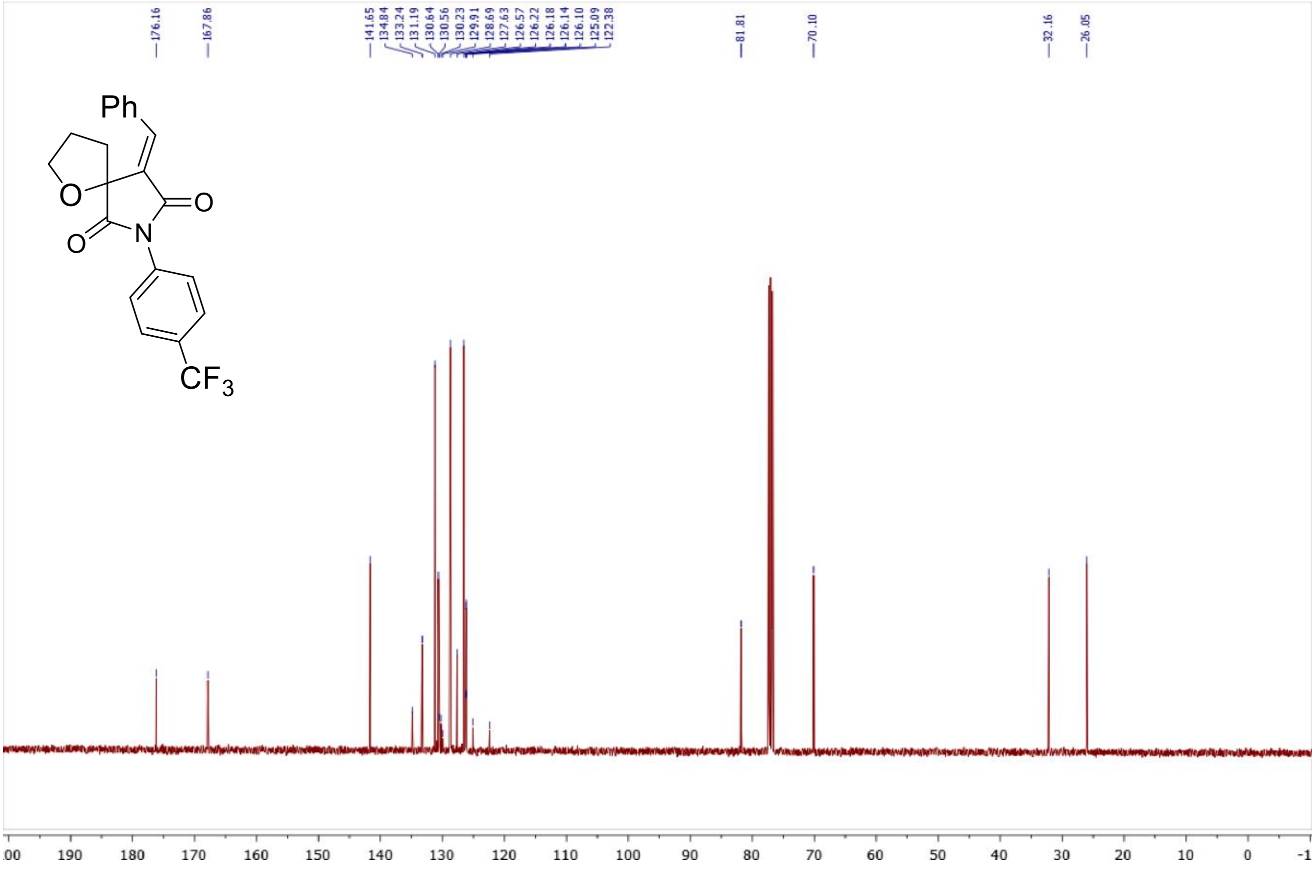

Copy of $^{19}F\{^1H\}$ (376.50 MHz, $CDCl_3$) spectrum of **4c**

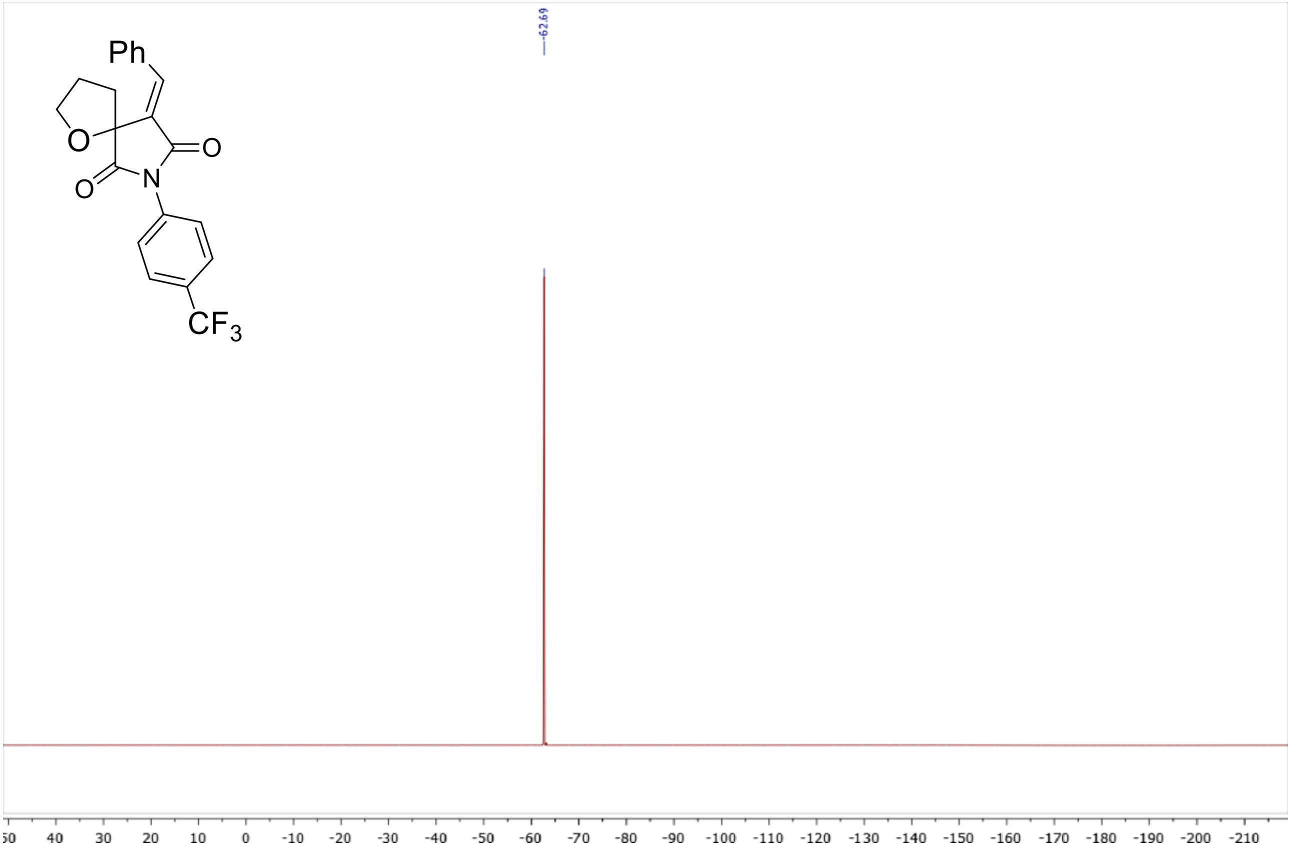

Copies of ^{1}H (400.13 MHz, $CDCl_3$) and $^{13}C\{^{1}H\}$ (100.61 MHz, $CDCl_3$) spectra of **4d**

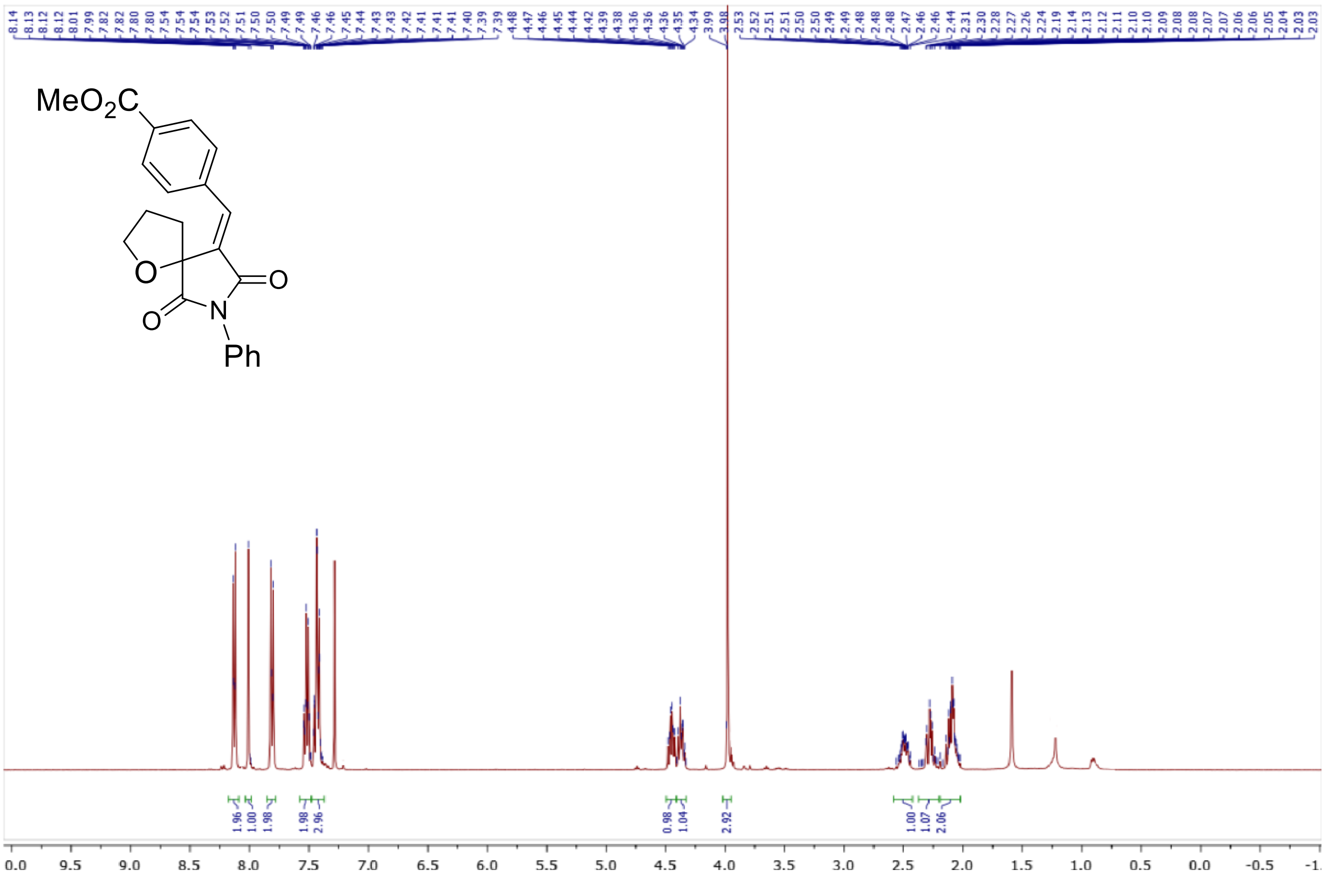

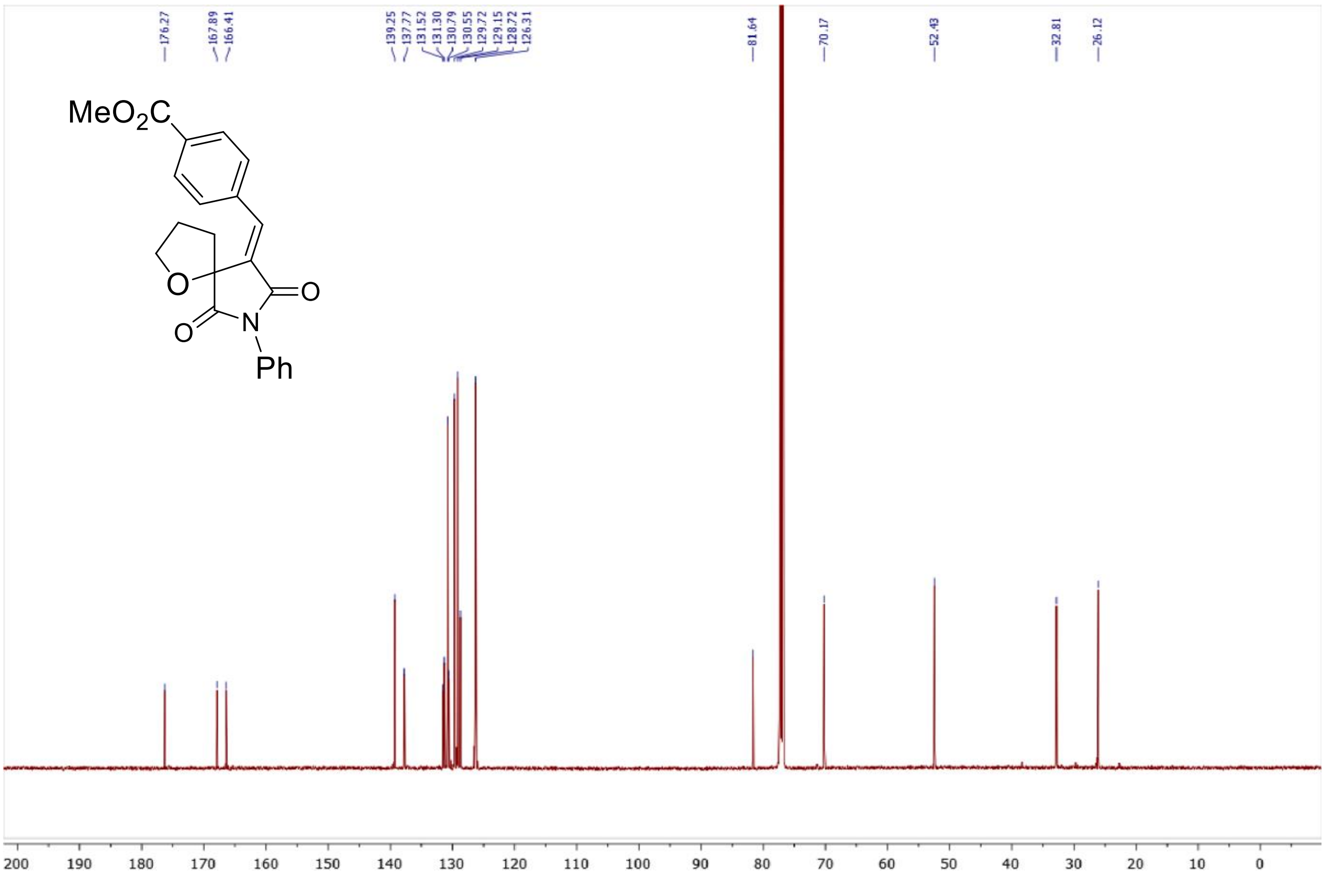

Copies of ^{1}H (400.13 MHz, $CDCl_3$) and $^{13}C\{^{1}H\}$ (100.61 MHz, $CDCl_3$) spectra of **5a**

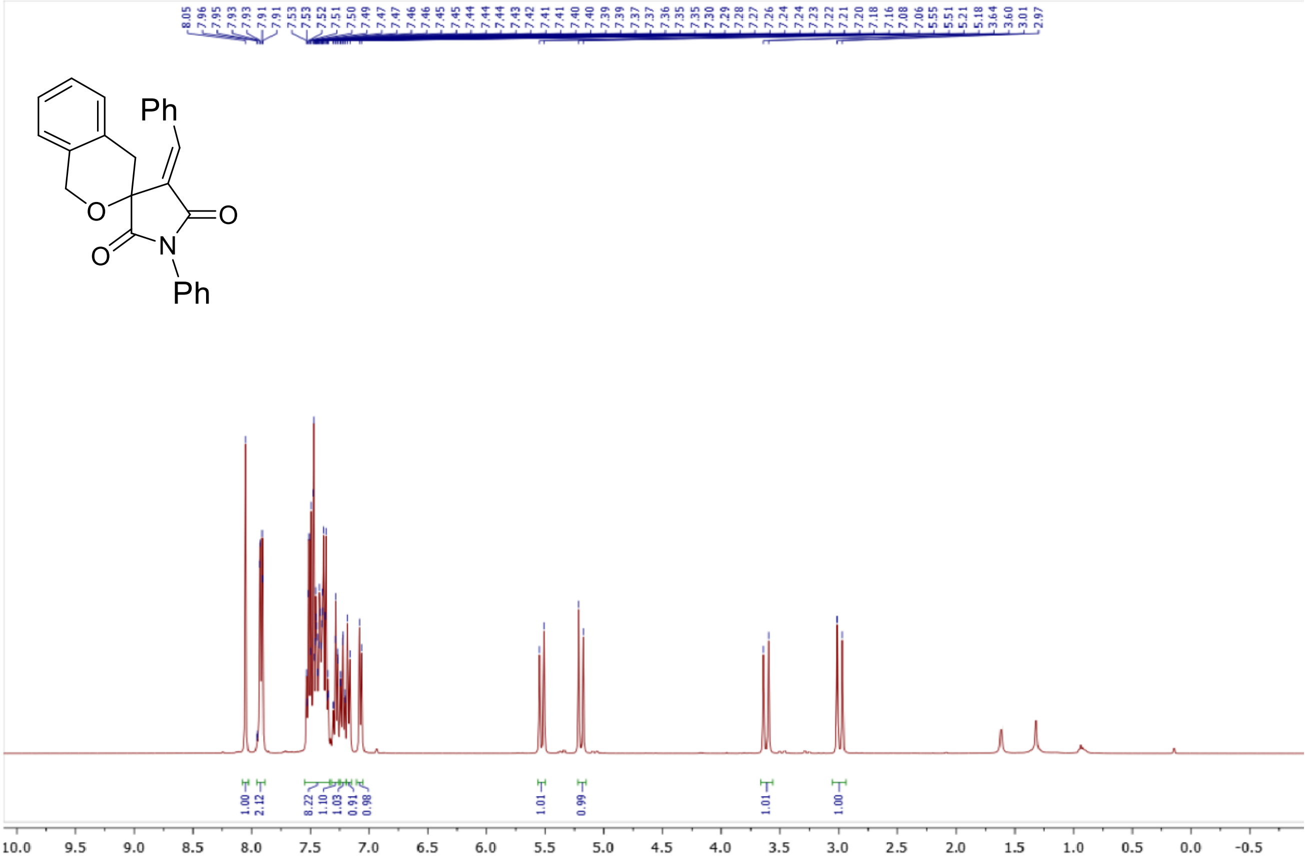

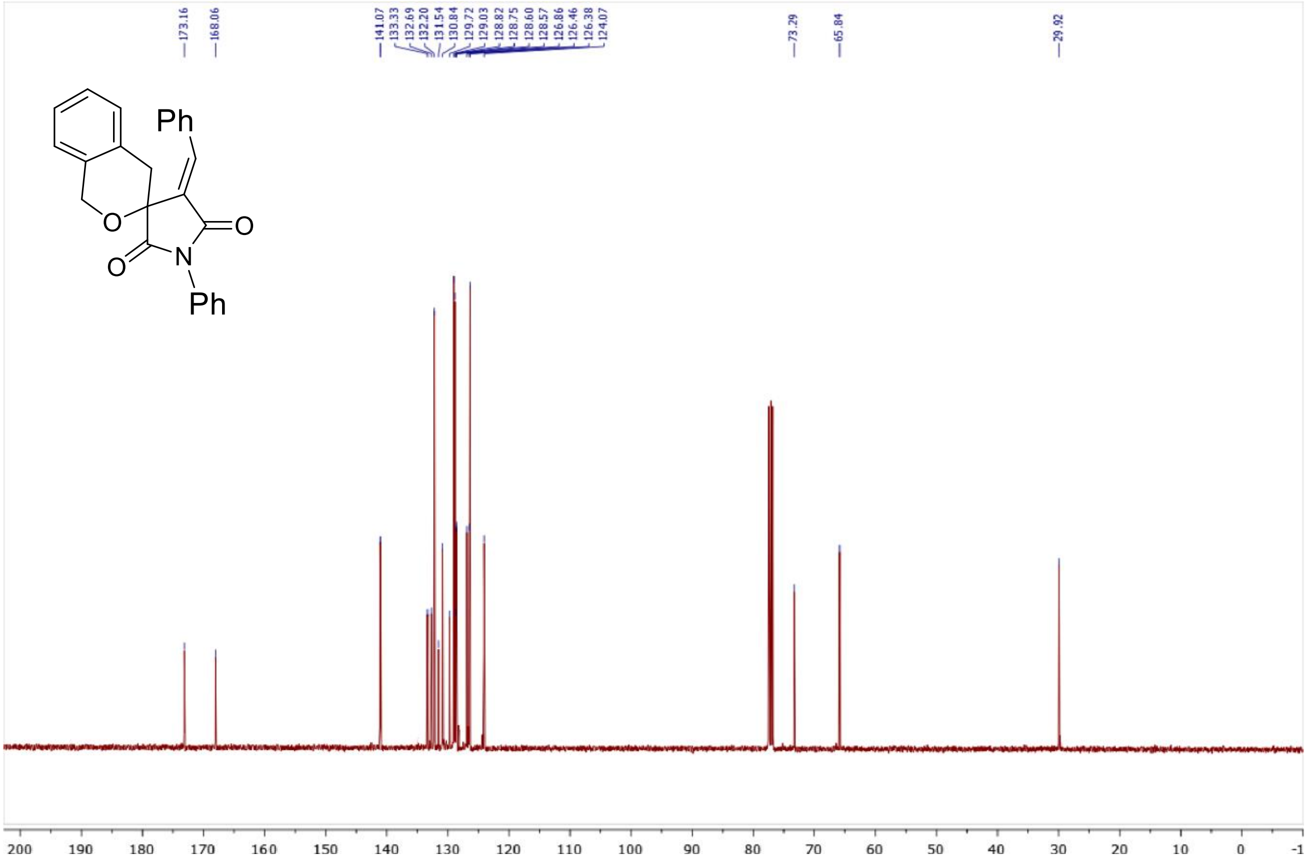

Copies of ^{1}H (400.13 MHz, $CDCl_3$) and $^{13}C\{^{1}H\}$ (100.61 MHz, $CDCl_3$) spectra of **5b**

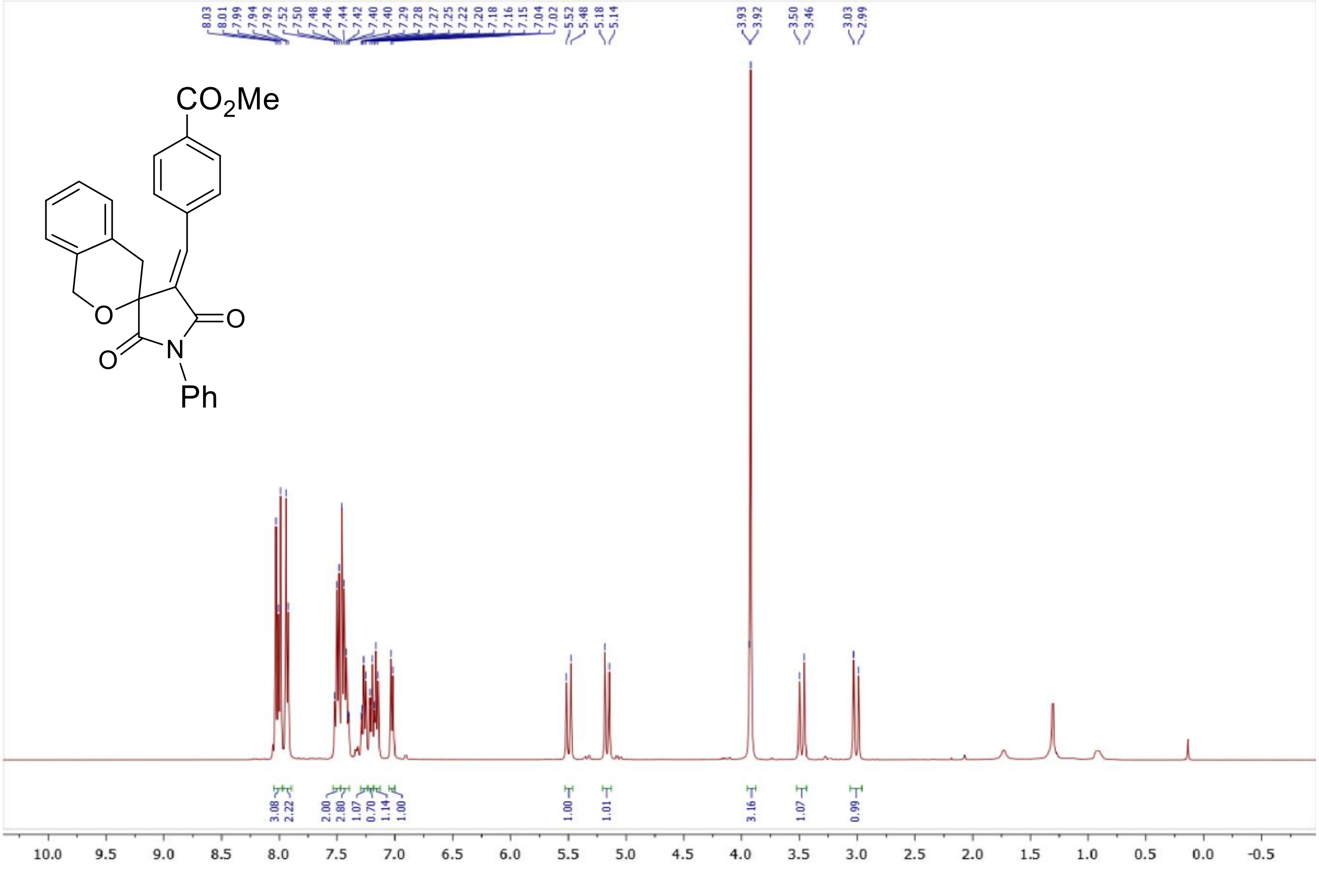

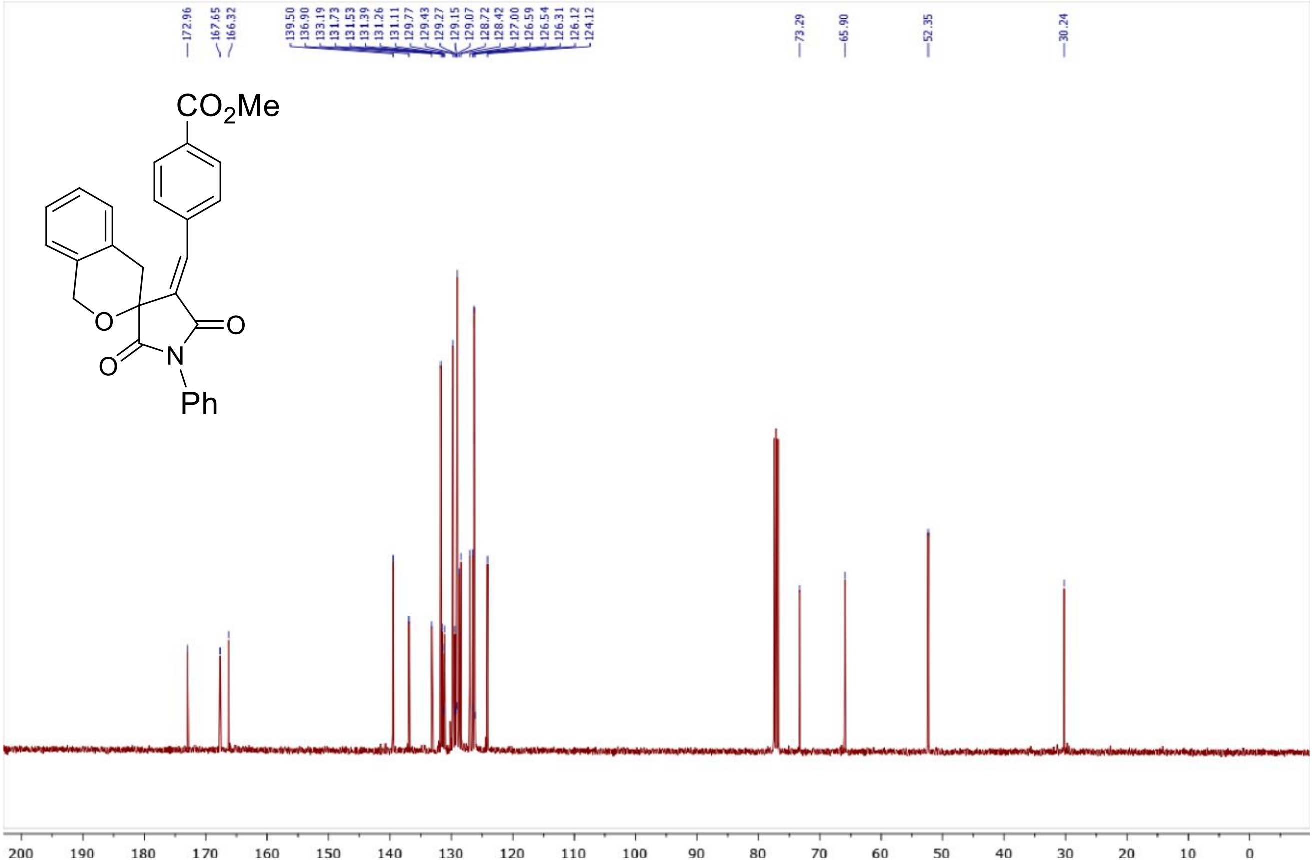

Copies of ^{1}H (400.13 MHz, $CDCl_3$) and $^{13}C\{^{1}H\}$ (100.61 MHz, $CDCl_3$) spectra of **5c**

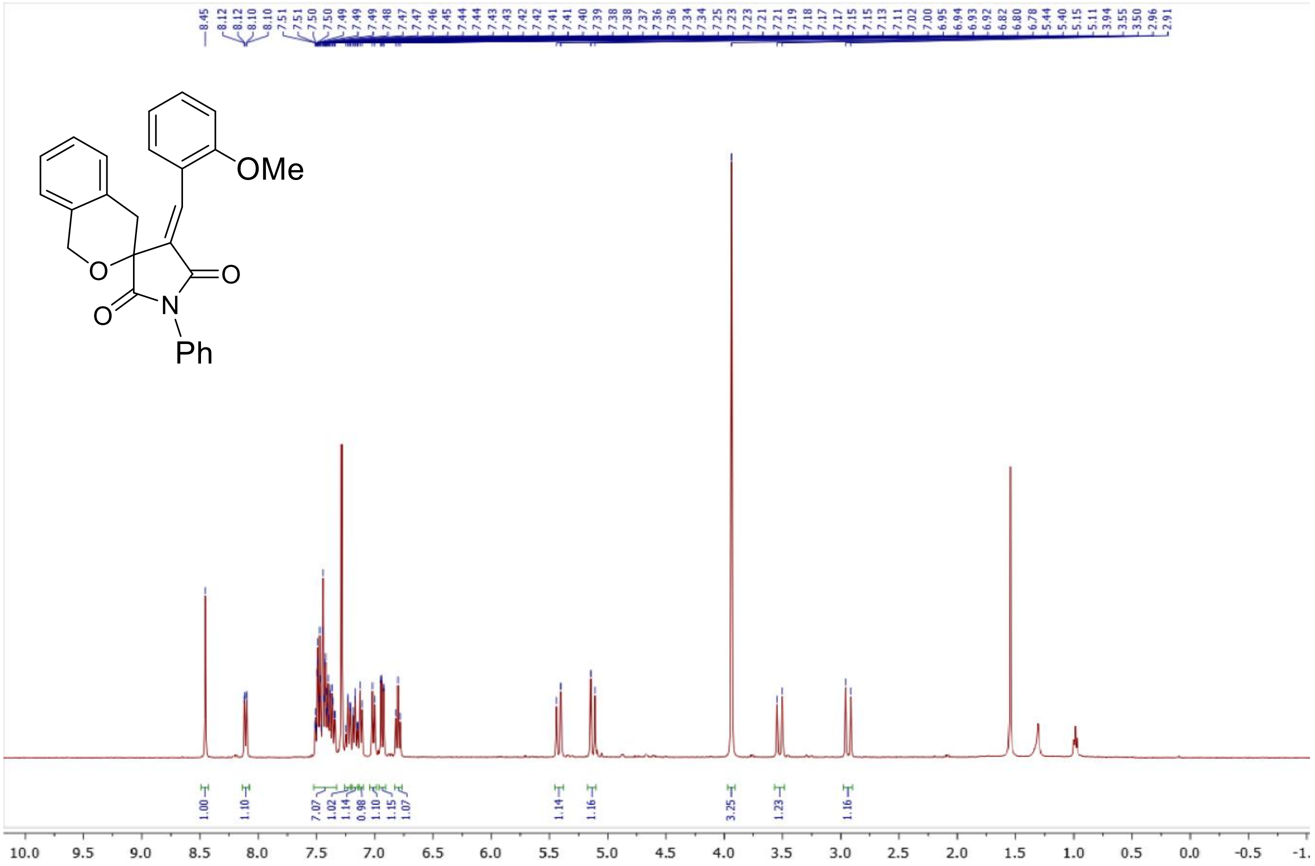

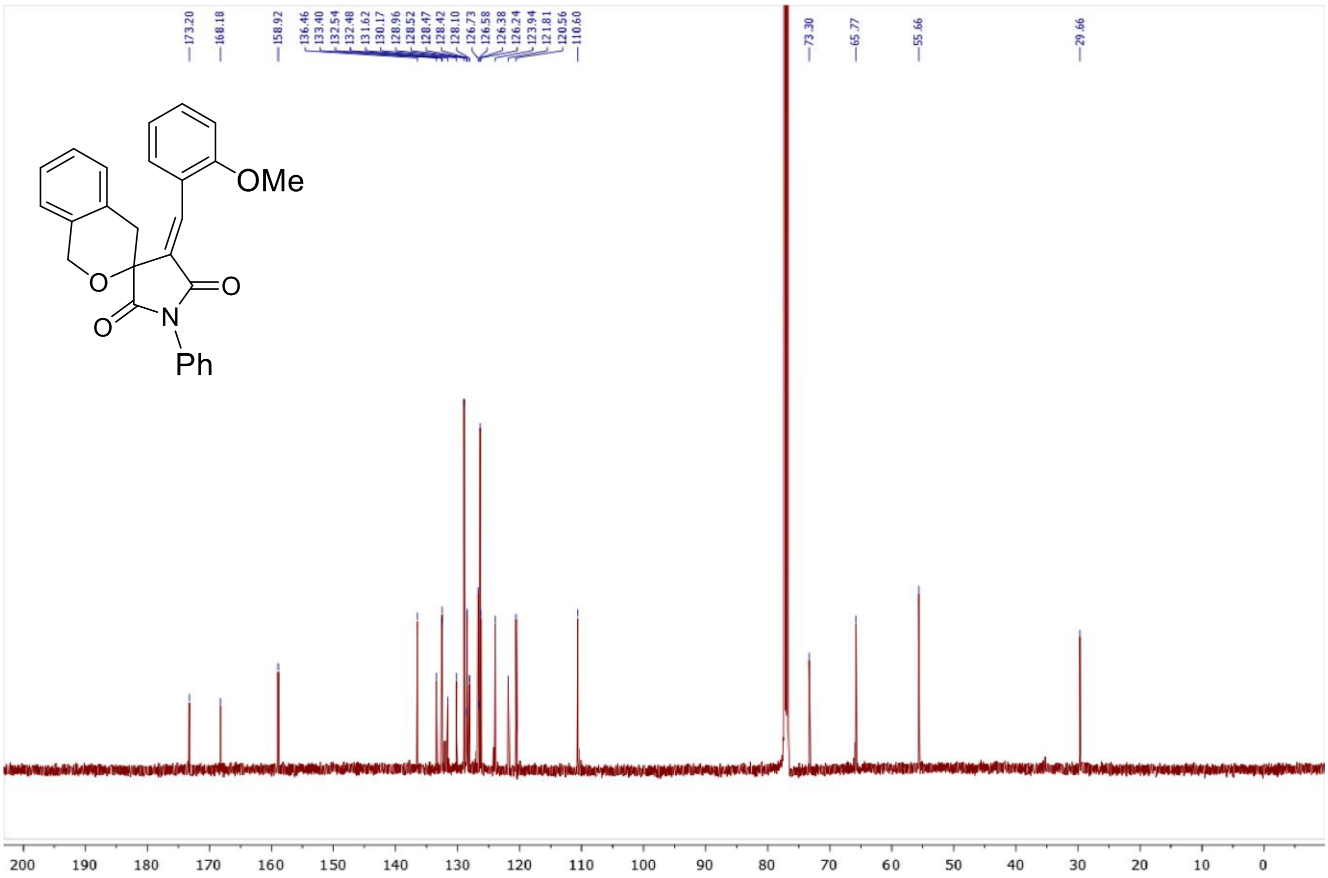

Copies of ^{1}H (400.13 MHz, $CDCl_3$) and $^{13}C\{^{1}H\}$ (100.61 MHz, $CDCl_3$) spectra of **18**

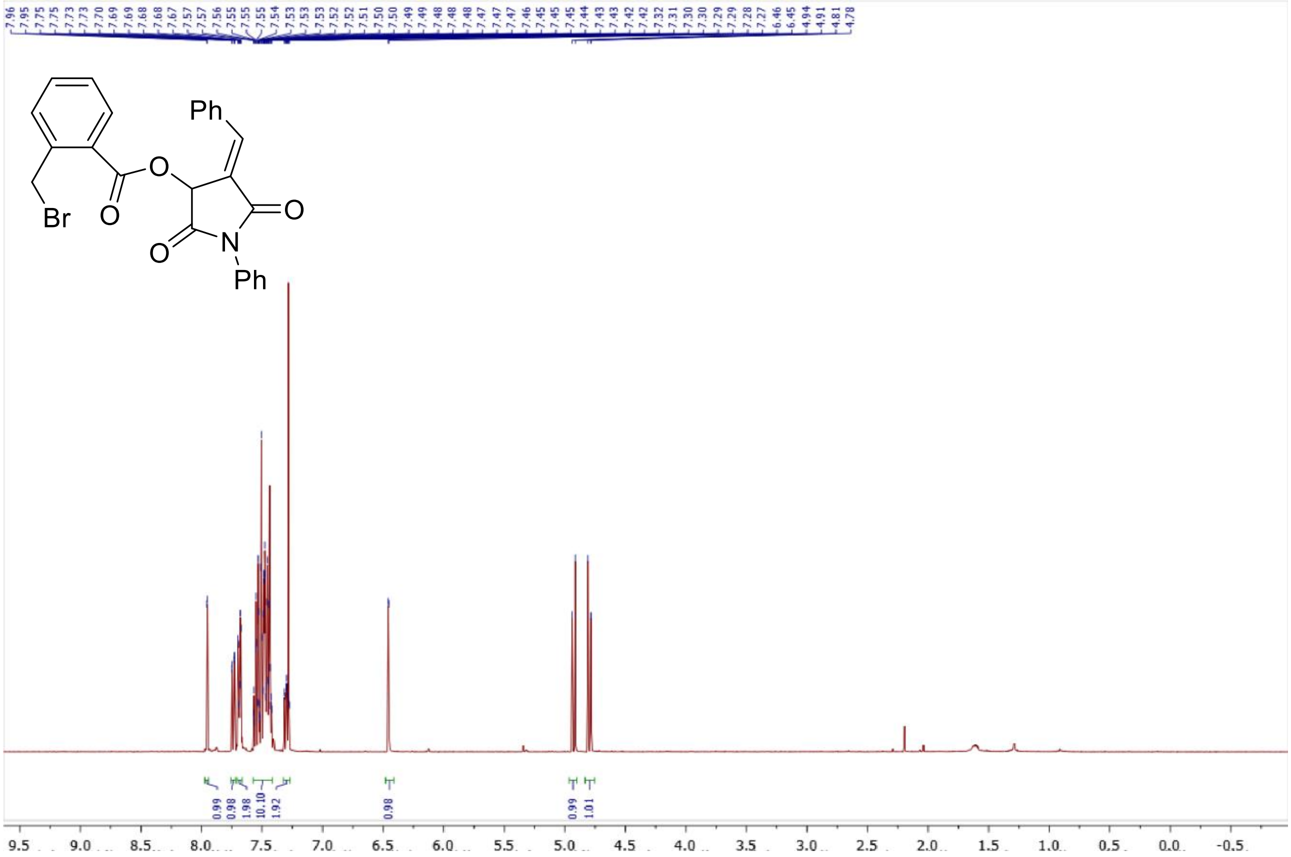

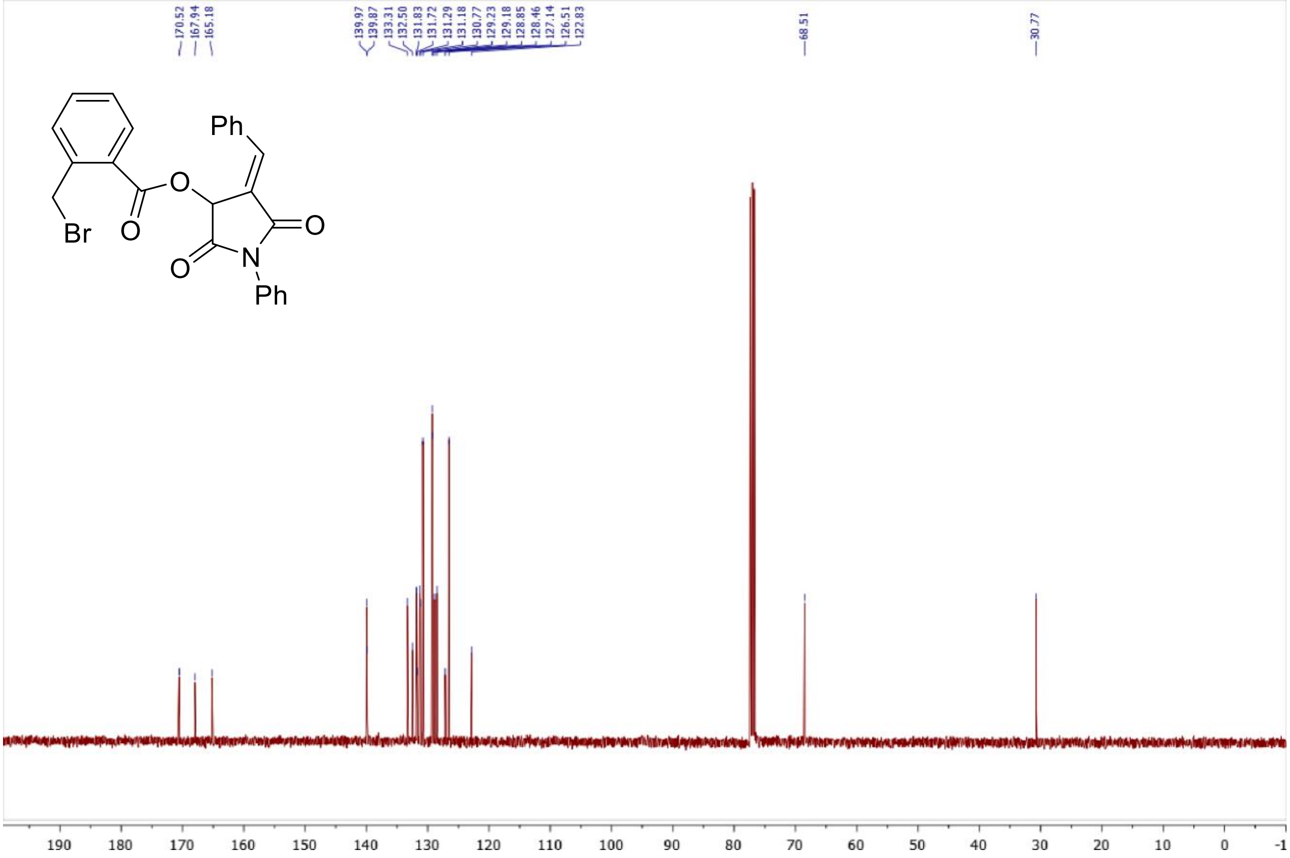

Copies of ^{1}H (400.13 MHz, $CDCl_3$) and $^{13}C\{^{1}H\}$ (100.61 MHz, $CDCl_3$) spectra of **21**

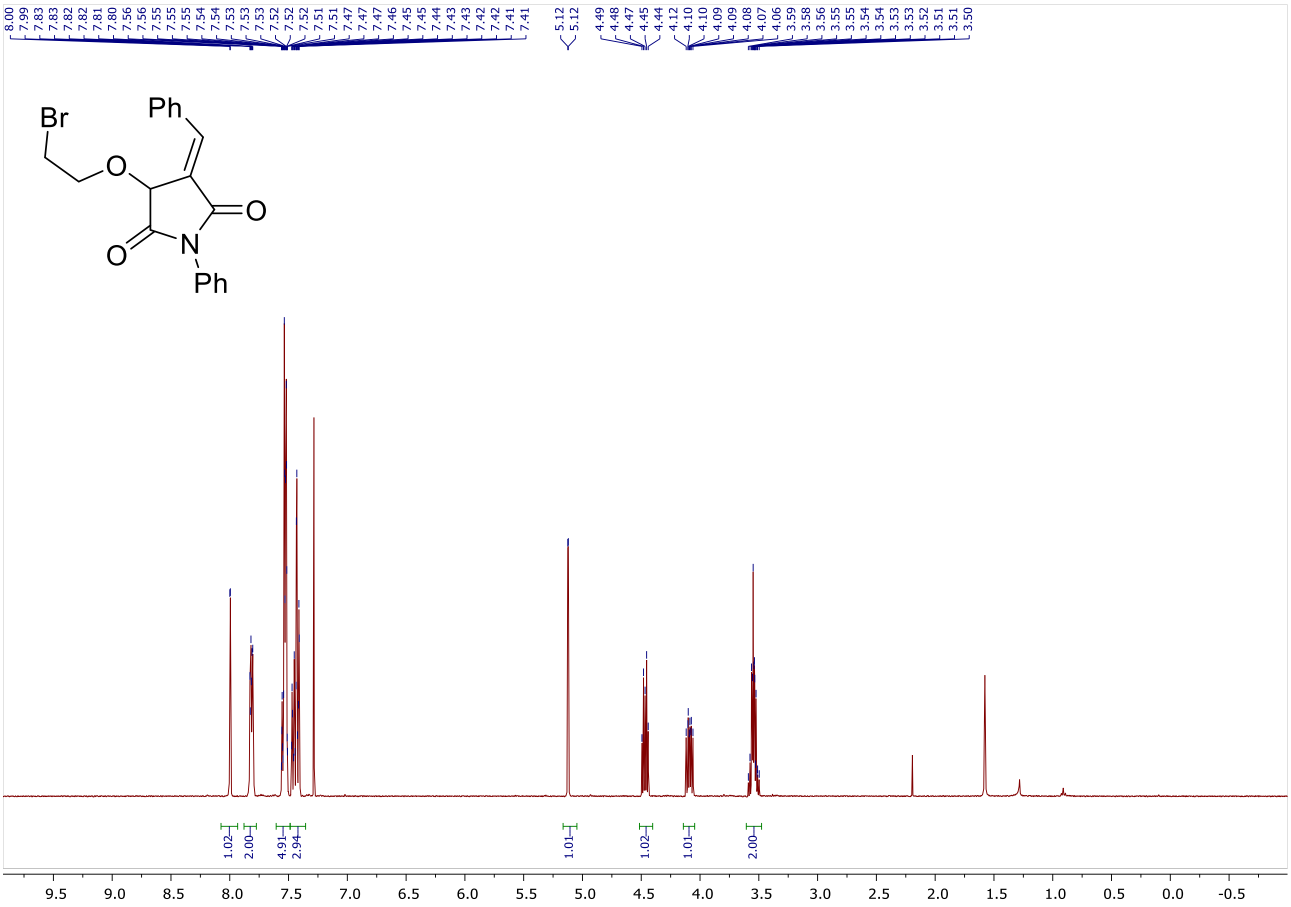

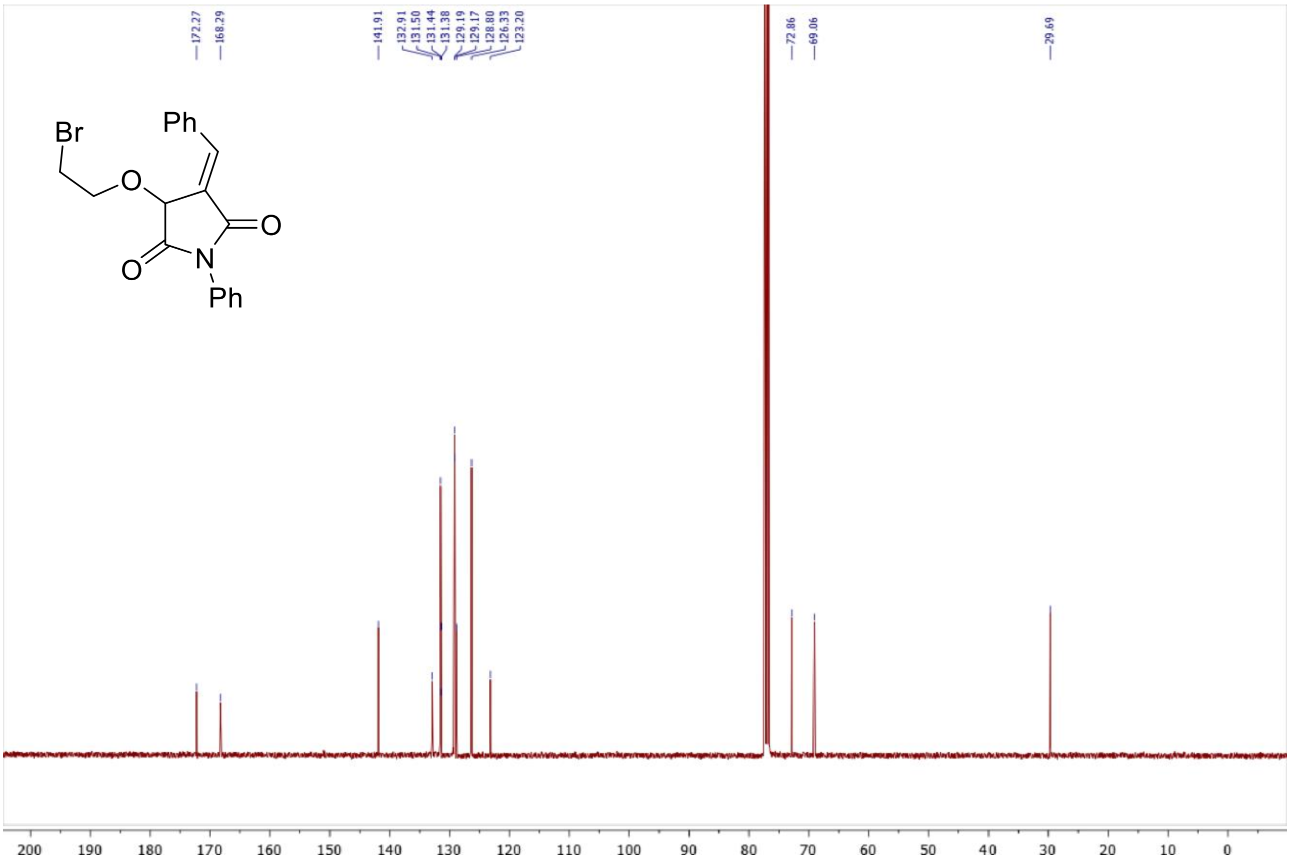

Copies of ^{1}H (400.13 MHz, $CDCl_3$) and $^{13}C\{^{1}H\}$ (100.61 MHz, $CDCl_3$) spectra of **25**

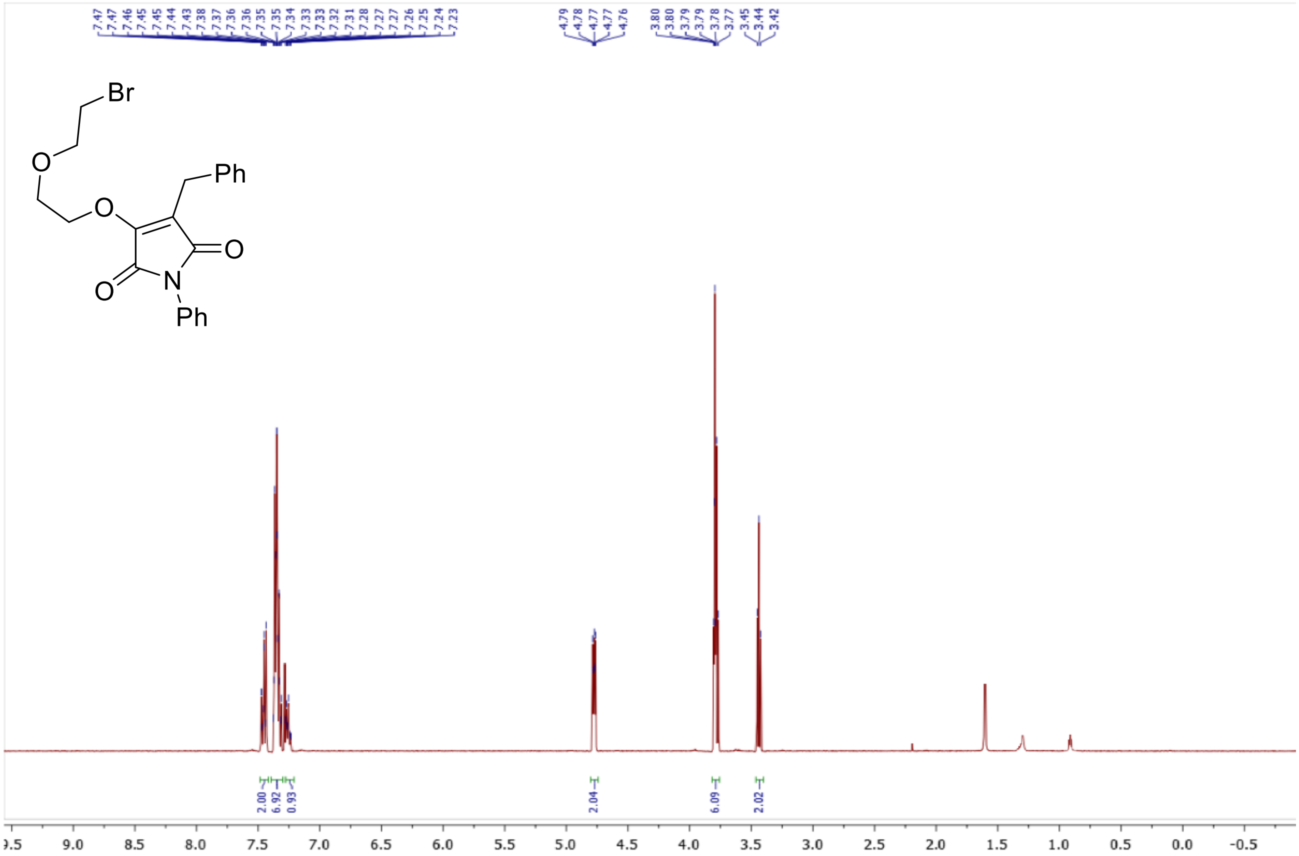

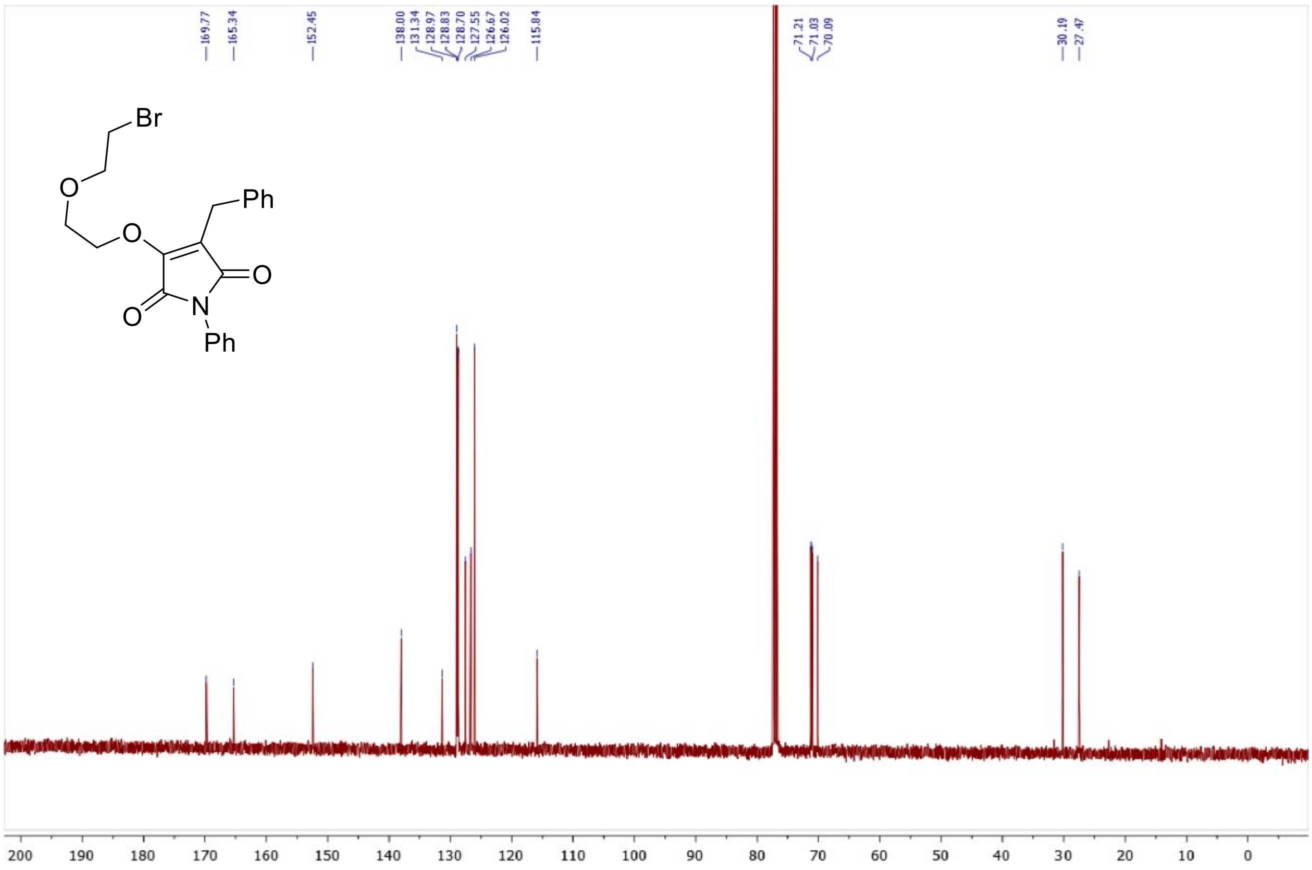

IV. X-ray crystallographic data

Crystallographic data for compounds **2a**, **3b**, and **4b**. Single crystal X-Ray analysis was performed on a SuperNova, Single source at offset/far, HyPix3000 diffractometer (**2a** and **3b**) and on a SuperNova, Dual, Cu at home/near, Atlas diffractometer (**4b**). Crystals were kept at 100(2) K during data collection. Using Olex2[5], the structures were solved with the SHELXT[6] structure solution program using Intrinsic Phasing and refined with the SHELXL[7] refinement package using Least Squares minimization. All hydrogen atoms were placed in accordance with neutron diffraction statistical data.[8]

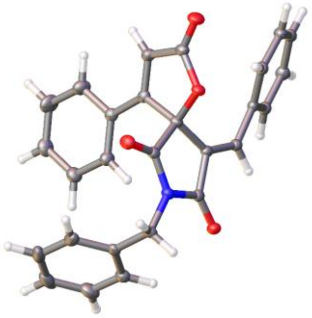

Figure S1. ORTEP representation of compound **2a** (thermal ellipsoids are shown at 50% probability)

Table S1. Crystal data and structure refinement for **2a**.

**CCDC**	**2295111**
Empirical formula	$C_{27}H_{19}NO_4$
Formula weight	421.43
Temperature/K	100(2)
Crystal system	triclinic
Space group	P-1
a/Å	9.6130(3)
b/Å	9.7625(2)
c/Å	12.5285(3)
α/°	110.885(2)
β/°	95.713(2)
γ/°	106.391(2)
Volume/Å^3	1027.26(5)
Z	2
ρ_{calc}g/cm^3	1.362
μ/mm^{-1}	0.745
F(000)	440.0
Crystal size/mm^3	0.2 × 0.18 × 0.12
Radiation	Cu Kα (λ = 1.54184)
2Θ range for data collection/°	7.748 to 159.994
Index ranges	-12 ≤ h ≤ 12, -12 ≤ k ≤ 10, -15 ≤ l ≤ 15
Reflections collected	12939
Independent reflections	4299 [R_{int} = 0.0346, R_{sigma} = 0.0340]
Data/restraints/parameters	4299/0/365
Goodness-of-fit on F^2	1.050
Final R indexes [I>=2σ (I)]	R_1 = 0.0399, wR_2 = 0.1052
Final R indexes [all data]	R_1 = 0.0426, wR_2 = 0.1086
Largest diff. peak/hole / e Å^{-3}	0.25/-0.26

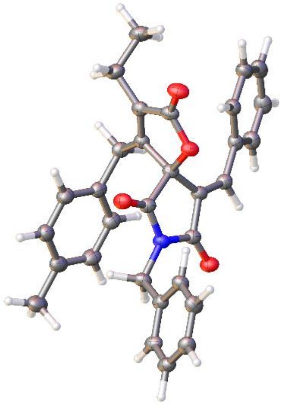

Figure S2. ORTEP representation of compound **3b** (thermal ellipsoids are shown at 50% probability).

**Table S2.** Crystal data and structure refinement for **3b**.	
**CCDC**	**2308315**
Empirical formula	$C_{31}H_{27}NO_4$
Formula weight	477.53
Temperature/K	100(2)
Crystal system	triclinic
Space group	P-1
a/Å	9.5933(4)
b/Å	10.4009(3)
c/Å	13.1112(3)
α/°	98.571(2)
β/°	100.754(3)
γ/°	101.537(3)
Volume/Å^3	1235.45(7)
Z	2
ρ_{calc}g/cm^3	1.284
μ/mm^{-1}	0.679
F(000)	504.0
Crystal size/mm^3	0.12 × 0.08 × 0.04
Radiation	Cu Kα (λ = 1.54184)
2Θ range for data collection/°	6.994 to 134.992
Index ranges	-11 ≤ h ≤ 11, -12 ≤ k ≤ 12, -14 ≤ l ≤ 15
Reflections collected	10664
Independent reflections	4453 [R_{int} = 0.0332, R_{sigma} = 0.0418]
Data/restraints/parameters	4453/0/327
Goodness-of-fit on F^2	1.086
Final R indexes [I>=2σ (I)]	R_1 = 0.0540, wR_2 = 0.1578
Final R indexes [all data]	R_1 = 0.0596, wR_2 = 0.1633
Largest diff. peak/hole / e Å^{-3}	0.35/-0.27

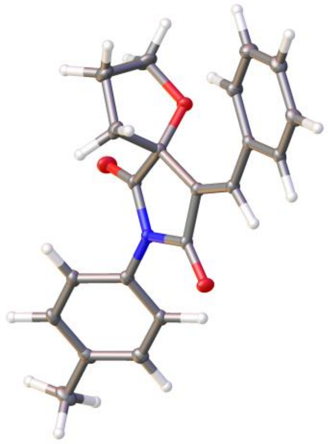

Figure S3. ORTEP representation of compound **4b** (thermal ellipsoids are shown at 50% probability).

Table S3. Crystal data and structure refinement for **4b**.

**CCDC**	**2305370**
Empirical formula	$C_{21}H_{19}NO_3$
Formula weight	333.37
Temperature/K	100(2)
Crystal system	monoclinic
Space group	$P2_1/c$
a/Å	6.8605(2)
b/Å	19.2835(4)
c/Å	12.6869(3)
α/°	90
β/°	98.215(2)
γ/°	90
Volume/Å^3	1661.18(7)
Z	4
ρ_{calc}g/cm^3	1.333
μ/mm^{-1}	0.719
F(000)	704.0
Crystal size/mm^3	0.7 × 0.19 × 0.13
Radiation	Cu Kα (λ = 1.54184)
2Θ range for data collection/°	8.402 to 152.286
Index ranges	$-8 \leq h \leq 8$, $-24 \leq k \leq 23$, $-13 \leq l \leq 15$
Reflections collected	11323
Independent reflections	3419 [R_{int} = 0.0538, R_{sigma} = 0.0430]
Data/restraints/parameters	3419/0/227
Goodness-of-fit on F^2	1.046
Final R indexes [I>=2σ (I)]	R_1 = 0.0470, wR_2 = 0.1240
Final R indexes [all data]	R_1 = 0.0547, wR_2 = 0.1318
Largest diff. peak/hole / e Å^{-3}	0.37/-0.33

V. References

1. Chupakhin, E. G.; Kantin, G. P.; Dar'in, D. V.; Krasavin, M. *Mendeleev Communications* **2021,** *31*, 36-38.
2. Lindsell, W. E.; Palmer, D. D.; Preston, P. N.; Rosair, G. M.; Jones, R. V. H.; Whitton, A. J. *Organometallics* **2005,** *24*, 1119-1133.
3. McNulty, J.; Keskar, K. *Organic & Biomolecular Chemistry* **2013,** *11*, 2404-2407.
4. Koley, D.; Krishna, Y.; Srinivas, K.; Khan, A. A.; Kant, R. *Angewandte Chemie International Edition* **2014,** *53*, 13196-13200.
5. Dolomanov, O. V.; Bourhis, L. J.; Gildea, R. J.; Howard, J. A. K.; Puschmann, H. *J. Appl. Crystallogr.* **2009,** *42*, 339-341.
6. Sheldrick, G. *Acta Crystallogr. A* **2015,** *71*, 3-8.
7. Sheldrick, G. *Acta Crystallogr. C* **2015,** *71*, 3-8.
8. Allen, F. H.; Bruno, I. J. *Acta Crystallogr. B* **2010,** *66*, 380-386.
